# Incompatible Translation Drives a Convergent Evolution and Viral Attenuation During the Development of Live Attenuated Vaccine

**DOI:** 10.3389/fcimb.2018.00249

**Published:** 2018-07-18

**Authors:** Xumin Ou, Mingshu Wang, Sai Mao, Jingyu Cao, Anchun Cheng, Dekang Zhu, Shun Chen, Renyong Jia, Mafeng Liu, Qiao Yang, Ying Wu, Xinxin Zhao, Shaqiu Zhang, Yunya Liu, Yanling Yu, Ling Zhang, Xiaoyue Chen, Maikel P. Peppelenbosch, Qiuwei Pan

**Affiliations:** ^1^Institute of Preventive Veterinary Medicine, Sichuan Agricultural University, Chengdu, China; ^2^Key Laboratory of Animal Disease and Human Health of Sichuan Province, Sichuan Agricultural University, Chengdu, China; ^3^Department of Gastroenterology and Hepatology, Erasmus MC-University Medical Center, Rotterdam, Netherlands; ^4^Avian Disease Research Center, College of Veterinary Medicine, Sichuan Agricultural University, Chengdu, China

**Keywords:** *Duck hepatitis A virus*, attenuation, RSCU, tRNA, translational selection, vaccine

## Abstract

Live attenuated vaccines are widely used to protect humans or animals from pathogen infections. We have previously developed a chicken embryo-attenuated Duck Hepatitis A Virus genotype 1 (DHAV-1) vaccine (CH60 strain). This study aims to understand the mechanisms that drive a virulent strain to an attenuated virus. Here, we systematically compared five DHAV-1 chicken embryo attenuated strains and 68 virulent strains. Phylogenetic analysis indicated that duck virulent strains isolated from different geographic regions of China undergo a convergent evolution in the chicken embryos. Comparative analysis indicated that the codon usage bias of the attenuated strains were shaped by chicken codons usage bias, which essentially contributed to viral adaption in the unsuitable host driven by incompatible translation. Of note, the missense mutations in coding region and mutations in untranslated regions may also contribute to viral attenuation of DHAV-1 to some extent. Importantly, we have experimentally confirmed that the expression levels of four viral proteins (${{\text{2A}}_{3}}^{\text{pro}}$, ${{\text{2A}}_{3}}^{\text{pro}}$, 3C^pro^, and 3D^pro^) in the liver and kidney of ducks infected with an attenuated strain are significantly lower than that infected with a virulent strain, despite with similar virus load. Thus, the key mechanisms of viral attenuation revealed by this study may lead to innovative and easy approaches in designing live attenuated vaccines.

## Introduction

Attributing to the effective immunogenicity and protection, live attenuated vaccines are widely used to protect humans or animals from certain pathogen infections since the recent half century (Bhamarapravati and Sutee, 2000; Belshe et al., 2007; Hviid et al., 2008; Song et al., 2013; Minor, 2016). One of the successful examples is the development of attenuated strains of Duck Hepatitis A Virus genotype 1 (DHAV-1) through serial passaging in chick embryos, which can provide perfect protection in immune ducklings (Cheng et al., 1993; Ou et al., 2017a). DHAV-1, a member of the family *Picornaviridae*, was recently classified into the unique genus of *Avihepatovirus* (http://www.ictvonline.org/virusTaxInfo.asp). It is highly pathogenic to ducklings less than 1 week old (morbidity and mortality, 100 and 95%, respectively; Salmon, 2013). We have previously established methods for virological and immunological detection of this virus and characterized the functions of several viral proteins (Anchun et al., 2009; Cao et al., 2012, 2016; Wen et al., 2014; Shen et al., 2015; Hu et al., 2016; Mao et al., 2016; Ou et al., 2016, 2017b; Yang et al., 2017; Zhang et al., 2017). However, the mechanisms of viral attenuation for the successful development of the DHAV vaccine remain largely elusive.

DHAV-1 is a single-stranded positive RNA virus with similar genomic organization of Picornavirus, including 5′ untranslated regions (UTR) covalently linked by a VPg, a large open reading frame (ORF), 3′UTR polyadenyliced by the PolyA tail (Kim et al., 2006; Racaniello, 2013; Sun et al., 2016). Once the viral RNA released into the host cells, it serves as a template for both translation and replication to assemble a huge number of progeny viruses (Tuthill et al., 2010; Racaniello, 2013; Wen et al., 2015). However, the expression of viral proteins largely relies on the host translation system. During passaging in chicken embryos, DHAV-1 triggers diverse pathological changes from slight to serious upon serial passaging (Hwang and Dougherty, 1962; Salmon, 2013). During this process, synonymous and non-synonymous mutations will emerge to make up the pressures from translation or function of viral proteins (Ran et al., 2014). The subsequent selections in the viral genome are the consequences of multiple host factors, including codon autocorrelation, clustering of rare codons, mRNA secondary structure, ribosomal density, relative abundance of wobble base pairs and modified tRNA (Novoa and De Pouplana, 2012).

The missense mutations change certain amino acid and may lead to structural or functional alterations of the protein. These may contribute to fitness or defection of the virus (Nilsson et al., 2003; Appel et al., 2008; Voitenleitner et al., 2012). It has been reported that missense mutations in nonstructural protein of hepatitis A virus (2B protein and 2C protein) and hepatitis C virus (NS5A and NS5B) will increase viral adaptation (Emerson et al., 1992; Lohmann et al., 2001; Voitenleitner et al., 2012). In contrast, synonymous mutations do not change the protein sequence. But they are not necessary to be neutral, because prefect codon is translated more efficiently, resulting in high levels of protein expression. Thus, we hypothesize that synonymous and missense mutations occurred during the passage in chick embryo are likely due to incompatibility of the host translation system. Because different types of hosts vary considerably in respect to the diversity of tRNA gene numbers, Relative Synonymous Codon Usage (RSCU) and tRNA modification enzymes (Novoa et al., 2012; Ran et al., 2014; Powell and Dion, 2015).

To address whether the incompatible translation system of the host causes viral attenuation, we first performed phylogenetic analysis of virulent and attenuated DHAV-1 strains to identify whether those attenuated strains undergo a similar evolution in chicken embryos. We then utilized the abundance of tRNA and RSCU in Gallus gallus and Anas Platyrhynchos genomes to understand the occurrence of those synonymous mutations upon serial passaging in chick embryos. Next, tertiary structural variations in structural and non-structural proteins of the virulent and attenuated strains were analyzed by homology modeling. Finally, we experimentally demonstrated the lower expression level of viral nonstructural proteins in the duck liver and kidney infected with the attenuated strains.

## Materials and methods

### Sequences of virus and host

The DHAV-1 CH60 attenuated strain is a commercial vaccine approved by Ministry of Agriculture (PRC). The attenuated strain, which was derived from the DHAV-1 CH strain in the allantoic cavities of 9-day-old specific pathogen free (SPF) chicken embryos after 60 passages, is a commercial vaccine developed by our laboratory. Other genomes of 72 Duck hepatitis A virus species were retrieved from GenBank (http://www.ncbi.nlm.nih.gov/genbank), including 4 strains of chicken embryo attenuated viruses, 68 strains of virulent viruses (Supplementary Table 1). The abundance of tRNA and RSCU of *Gallus gallus* and *Anas Platyrhynchos* were obtained from Genbank and Condon Usage Bias Databases, respectively (http://www.kazusa.or.jp/codon/).

### Phylogenetic analysis

To elucidate the phylogenetic relationship between chick embryo attenuated strains and virulent strains, 68 virulent strains and 5 chick embryos attenuated strains were analyzed by Neighbor joining method using Mega 6.0 (Tamura et al., 2013).

### Codon usage indices

Nucleotides frequencies at the third position of whole genome was calculated by bio-python software. The RSCU, Uracil at the 3rd codon position (U3s), A3s, G3s, C3s and the third synonymous codon position GC content (GC3s) were calculated by CodonW (http://www.molbiol.ox.ac.uk/cu, version 1.4.2) using Saccharomyces cerevisiae as reference. RSCU used by virulent and attenuated strains were visualized by Hemi software with hierarchical clustering analysis (http://hemi.biocuckoo.org/).

### Correlation analysis of RSCU and tRNA copies in chicken and duck genomes

Those significant changed codons in chick embryo attenuated strains were correlated with RSCU and tRNA copies in chicken and duck, respectively. The corresponding codons in virulent strains were also analyzed by the above methods. Linear regression and significant correlations were calculated using IBM SPSS Statistic 20. The RSCU and tRNA copies used in chicken and duck genome were plotted by Graphpad Prim software. The NNA, NNT, NNG, and NNC used in chicken and duck were compared by Wilcoxon matched-pairs signed-ranks test. tRNA^TNN^, tRNA^ANN^, tRNA^CNN^, and tRNA^GNN^ were also compared. ^*^P < 0.05, ^**^P < 0.01, or ^***^P < 0.001.

### Multiple sequences alignment

Conforming nucleotide substitutions were identified by Mega 6.0 using Cluster W method (Tamura et al., 2013). The details of synonymous and non-synonymous mutations were listed in Supplementary Table 2. The frequencies of nucleotides in ORF (68 virulent strain and 5 attenuated strain) were calculated by biopython analysis. The nucleotide with the highest frequencies at each site was used to construct two conserved ORF sequence (virulent strain and attenuated strain).

### Secondary structural prediction of 5′UTR and 3′UTR

The 5′UTR and 3′UTR of DHAV-1 virulent (ATCC, DRL-62 strain) and chick embryo attenuated virus (C80 strain) were imported into RNAfold web server to predict their secondary structures (Gruber et al., 2008). The sequences and CT files were visualized by Vienna VARNA package. In order to understand whether those fixed SNPs could significantly change the secondary structure of virulent strains, the 5′UTR and 3′UTR of artificial mutant 5′UTR or 3′UTR were also imported into RNAfold web server to predict their secondary structure (Supplementary Table 2).

### Experimental design and immunohistochemistry (IHC)

Fifteen ducks were randomly divided into three groups. Group 1 (CH60 strain) and group 2 (H strain) received 1 ml of virus (4.56 × 10^8^ copies/ml) by intramuscular injection, while group 3 was injected with an equal volume of 0.85% physiological saline as a negative control. The tissues of the liver and kidney from the mature ducks (160 days of age) inoculated with virulent strains and chicken embryo attenuated strains at 4 days post-inoculation were fixed in 4% paraformaldehyde, dehydrated, embedded in paraffin, sectioned into 4-μm thick sections and followed with previous established IHC protocol (Ou et al., 2017a).

### qRT-PCR

Virus loads in liver and kidney were detected by real-time PCR assay according to the previously established method (Yang et al., 2008). Briefly, one hundred milligrams of each tissue were used for RNA isolation, then the viral RNA copies was detected by one step Real-time PCR assay. The viral RNA copies in liver or kidney were translated by copies (Log_10_/g).

### Homologous modeling

Online homologous modeling software of SWISS-MODEL was used to analysis the structural variations resulted from missense mutations on Table S2 in the process of serial passages in chicken embryos (Biasini et al., 2014). It base sensitive Hidden Markov Models (HMM) to search against the SWISS-MODEL Template Library (SMTL). And those amino acid (aa) substitutes were demonstrated by the PyMOL molecular graphics system (DeLano, 2002; Supplemental Figures 2–4). Templates for homologous modeling are as follows, Ljiugan viral capsids (PDB:3Jb4: A/B/C) for modeling DHAV-1 VP1/0/3 (Zhu et al., 2015), GTPase IMAP family member 7 (PDB: 3zjc.3.A) for 2A2 protein (Schwefel et al., 2013), Adipose phospholipase A (PDB: 4fa0.1.A) for 2A3 protein (Pang et al., 2012), Actin-related protein 2/3 complex subunit 3(PDB: 4jd2.1.E) for 2B protein (Luan and Nolen, 2013), Minichromosome maintenance protein (MCM)(PDB: 4r7y.1.A) for 2C protein (Miller et al., 2014), AopB (PDB: 3wxx.2.B) for 3A protein (Nguyen et al., 2015), iron-regulated surface determinant protein H (PDB: 2lhr.1.A) for 3B protein (Spirig et al., 2013), Enterovirus 71 3C protein (PDB: 3qzq.4.A) for 3C protein (Wang et al., 2011), Poliovirus 3D polymerase (PDB: 4nlr.1.A)for 3D protein (Sholders and Peersen, 2014).

## Results

### Convergent evolution of the virulent strains upon passaging in chicken embryos

**Figure 1:**
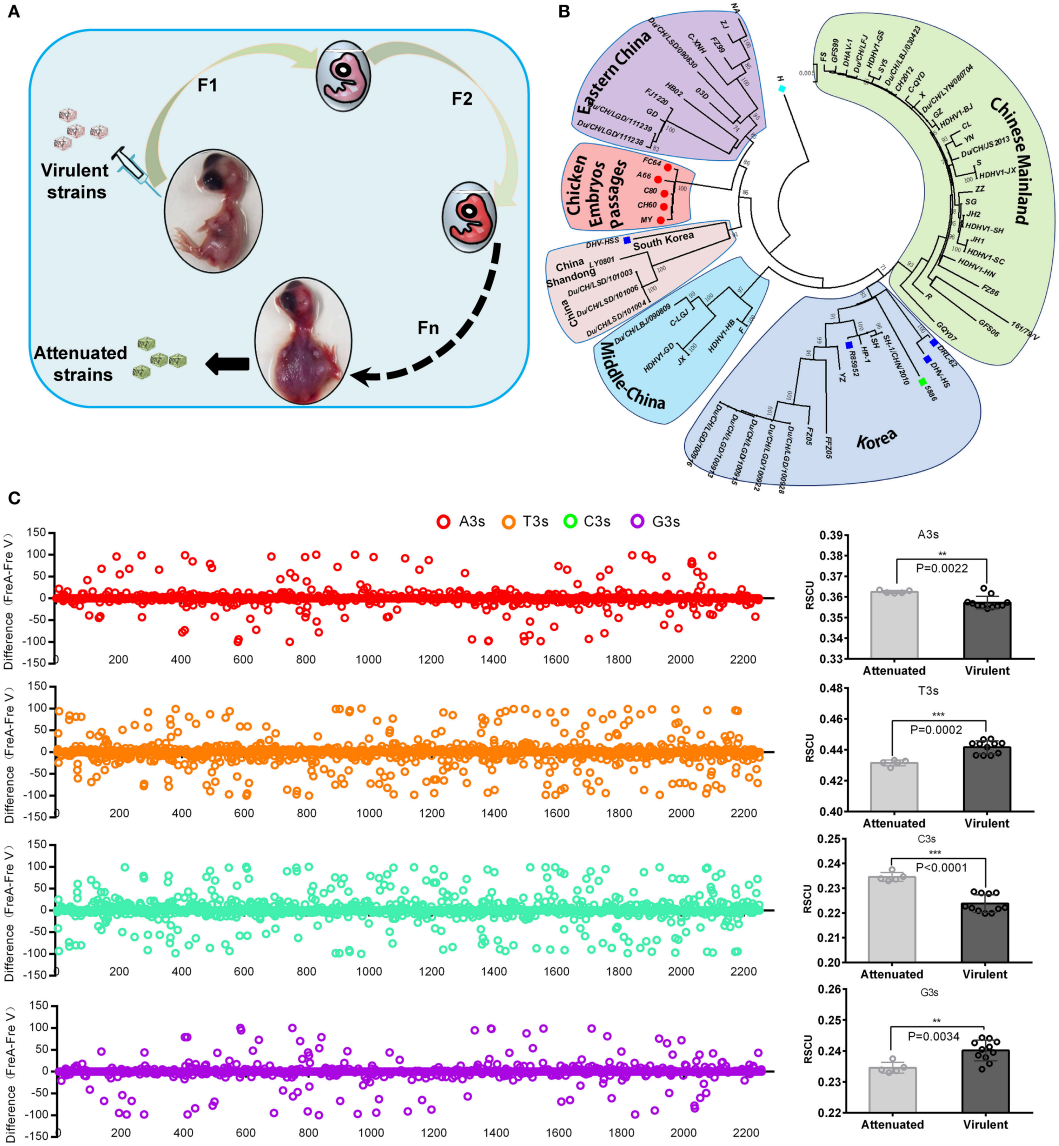
Overview of viral attenuation in chicken embryos and host selection on virus. **(A)** Virulent strains isolated from ducklings can be attenuated through series passaging in chicken embryos, which accompanied by seriously histopathological injury after 60 passages (Hwang and Dougherty, 1962). **(B)** Phylogenetic analysis indicated virulent strains isolated from different regions caused a convergent evolution under similar host selection-chicken embryos. In addition, genetic distance was much closer than virulent strains. The evolutionary tree was inferred using the Neighbor-Joining method with 1000 bootstrap test. **(C)** Thymine at the 3rd codon position (T3s), A3s, G3s, C3s between the attenuated and virulent strains were compared in whole genome and sum (Left and right). The differences were calculated by N3s frequencies of attenuated strains minus N3s frequencies of virulent strains (Fre A-Fre V). The total N3s were also compared by Student *T*-test. **P < 0.01, or ***P < 0.001.

In general, virulent strains of DHAV-1 isolated from ducklings can be attenuated through series of passaging in chicken embryos, accompanied by histopathological injury from mild to severe (Figure 1A; Hwang and Dougherty, 1962). Phylogenetic analysis indicated a convergent evolution of the virulent strains isolated from different regions under the host selection of chicken embryos (Figure 1B). Genomic and protein sequence alignment between chick embryo attenuated strains and duck virulent strains indicated that a large number of identical mutations were selected in those five attenuated strains (Supplementary Data 1–2). next, the nucleotide frequencies at the third position were compared between attenuated and virulent strains (Figure 1C). To estimate the percentage of synonymous and non-synonymous, the frequencies of nucleotides at each site were used to construct two conserved viral ORFs (virulent strain and attenuated strain). As calculated, the total mutations in the ORF is 208, when compared to the above constructed sequence. Specifically, 30 out of 208 mutations will lead to 27 non-synonymous mutations. Therefore, the percentage of synonymous mutation is 85.57% (178/208 = 0.8557) (Supplementary Data 3). Those results indicated that codon usage bias was apparently changed after passaging in chick embryos. Specifically, Adenine at the 3rd codon position (A3s) and C3s in chicken embryo attenuated strains is much more abundant than that in virulent strains (*P* < 0.01), but much less at T3s and G3s (Figure 1C). During the process of passaging, a number of SNPs were also acquired (Supplementary Table 2). These results indicated convergent evolution of genomic sequence and the pattern of codon usage of DHAV-1 during serial passage in chick embryo.

### Incompatible host translation shapes viral codon usage bias

**Figure 2:**
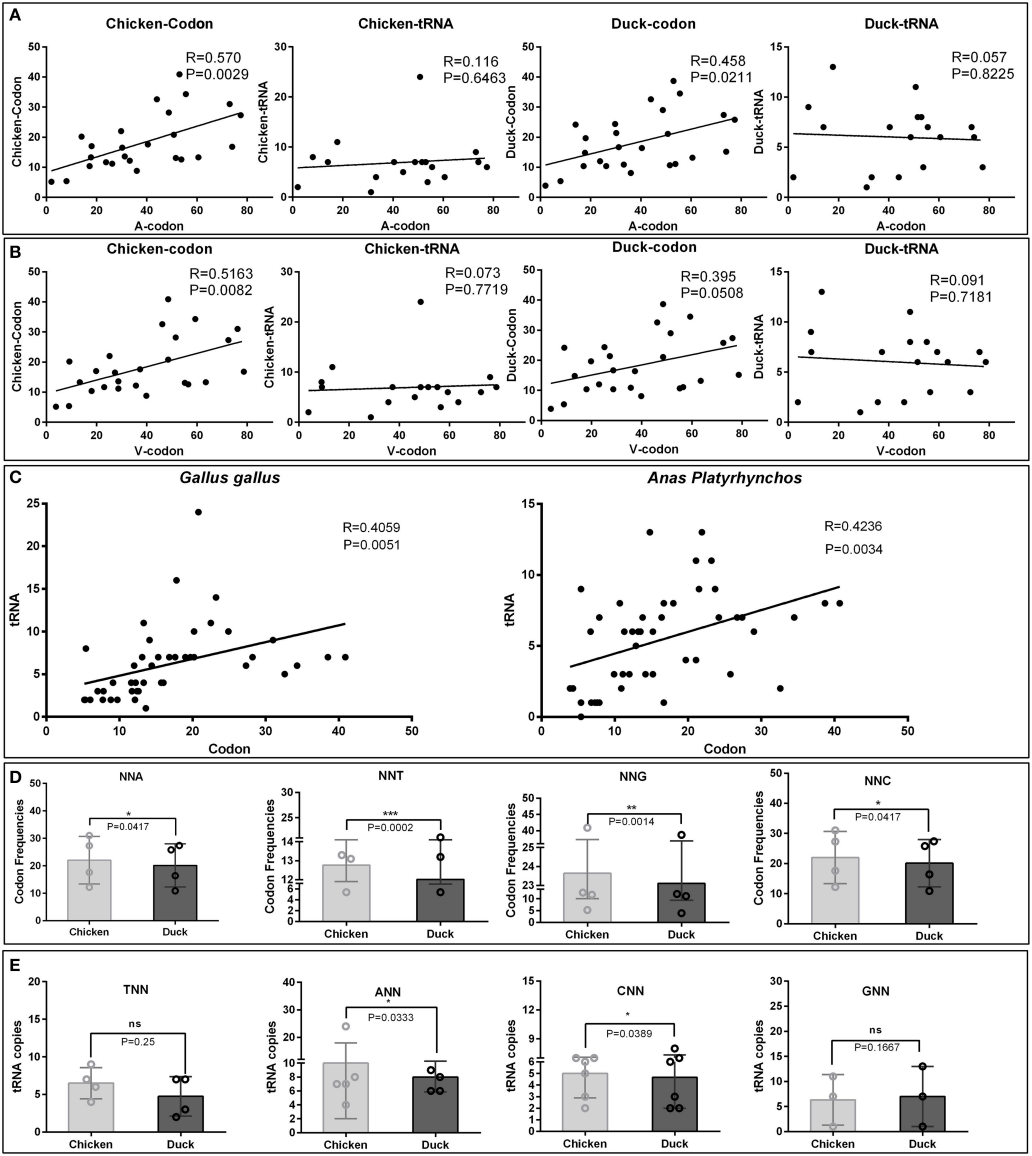
Comparative analysis of codon usage frequencies and tRNA copies in chickens and ducks. To elucidate the potential mechanism of codons bias in chick embryo attenuated strains, the corresponding RSCU and tRNA copies in chickens and ducks were analyzed. **(A)** Those significant changed codons in the attenuated strains were correlated with corresponding codon usage frequencies and tRNA copies in chicken and ducks. Correlation index (R) and significant levels (*P*-values) were also listed. **(B)** The counterparts in the virulent strains were also analyzed by the same methods. **(C)** Correlation between codon usage frequencies and tRNA copies in chicken and duck were both significant correlated (*P* < 0.05). **(D)** NNA, NNT, NNG, and NNC used in chicken and duck were compared. **(E)** tRNA^TNN^, tRNA^ANN^, tRNA^CNN^, and tRNA^GNN^ used in chicken and ducks were compared. The difference of each groups were analysis by Wilcoxon matched-pairs signed-ranks test. *P < 0.05, **P < 0.01, or ***P < 0.001.

The virulent viruses insolated from duck are originally non-infectious to chicken. While after series of passages in chicken embryos, it indeed adapt the new host (Hwang and Dougherty, 1962). When we inoculate back this chicken attenuated virus to their original host, duck, virulence is attenuated. In all organism, protein translation is the last step to readout genetic codons. While different host translation machinery uses different decoding strategy, the major reason is diversity of codon usage bias used by different host, as chicken and duck in this study. Importantly, it had been proven that the expression of viral genes largely relies on the host translation system, and bias codons that is compatible to their host can improve translation efficiencies and protein fold (Hanson and Coller, 2017). To elucidate the potential mechanism of codon bias in chick embryo attenuated strains, the corresponding codon frequencies and tRNA copies in chickens and ducks were simultaneously analyzed with viral codon usage bias (Supplementary Tables 3–5). The correlation analysis indicates that the codon frequencies in attenuated strains are higher (24.45%) in correlation with chicken codon frequencies than that in duck [Correlation index (0.570 vs. 0.458)]. But those codon frequencies are not correlated with tRNA copies in both chicken and duck genome (Figure 2A). Interestingly, the corresponding codon frequencies in virulent strains are also higher (30.70%) in correlation with chicken codon frequencies than that in duck [Correlation index (0.5163 vs. 0.395)]. Those codon frequencies in virulent strains are not correlated with tRNA copies in both chicken and duck genome (Figure 2B). In the genomes of both *Gallus gallus* and *Anas platyrhynchos*, the codon frequencies per thousand is positively correlated with the abundance of tRNA in its genome respectively (*P* < 0.01) (Figure 2C). The corresponding codons, NNA, NNT, NNG and NNC, used in chicken are significantly higher than that in duck (*P* < 0.05 at least) (Figure 2D). Since tRNA copies have significant impact on codon usage bias, the corresponding tRNA copies were also analyzed. tRNA^ANN^ and tRNA^CNN^, but not tRNA^TNN^ and tRNA^GNN^, are significantly higher than that in duck (*P* < 0.05) (Figure 2E). Altogether, the higher correlation of codon frequencies in chicken embryo attenuated strains and their new host (chicken) indicated that viral codon usage bias was shaped by incompatible host translation that duck insolated virus must adapt in chicken embryos by using this incompatible host translation.

### High variations in secondary structure of 5′UTR but not 3′UTR of the DHAV-1 genome

Because viral 5′UTR and 3′UTR are constructively needed for IRES-mediated translation. Their secondary structural variations could regulate the efficiency of viral translation. Therefore, the secondary structures of 5′UTR and 3′UTR of the DHAV-1 virulent strain (ATCC, DRL-62) and chick embryo attenuated strain (C80) were predicted by RNAfold web server according to the rule of minimum free energy (MFE) (Gruber et al., 2008). We next analyzed whether the confirmed mutations in Supplementary Table 2 can lead to a secondary structural variations or not. We found that the secondary structure at 5′UTR was heavily changed during the process of serial passaging in chick embryos and this was consistent with DRL-62 mutation analysis (Supplementary Figure 1a). However, there was only one nucleotide substitution (145, A-G) occurred at 3'UTR which led to a slight extension of a central stem (Supplementary Figure 1b).

### Tertiary structural variations in both virulent strains and chicken embryo attenuated strains

To further understand structural variations of viral proteome caused by those missense mutations, comparative homology modeling was used. In P1-coded viral capsid (P1), four substitutes (T3S, E205K, R217K, D234N) in the VP1 and three substitutes (P55L, T163A, A168T) in the VP0 were displayed in the Supplementary Figure 2. These mutations are mainly located within the interface between VP1 and VP0. Except for the rest of P2 region, 2C^pro^ was the only one truncated into three fragments to build its tertiary structure, including 2C_1−137_ similar to Minichromosome Maintenance Protein (MCM) terminal, 2C_138−264_ to major MCM, 2C_265−333_ to central domain of MCM (Supplementary Figure 3d). The mutation H142Y nearby G4 box in ${\text{2A}}_{2}^{\text{pro}}$, which changed from a basic aa to an aromatic aa, may attribute to the effect of maladaptive chick embryos. In the P3-region, only one substitute (E30G) in 3B^pro^ and four substitutes (G46E, C89S, D91E, and L434F) in 3D^pro^ were identified. Those mutations focus on the surface loop of 3D^pro^ except for the central alpha-helix of 2B^pro^ (Supplementary Figure 4d).

### Lower expression levels of viral nonstructural proteins in duck liver and kidney infected with chicken embryo attenuated DHAV-1

**Figure 3:**
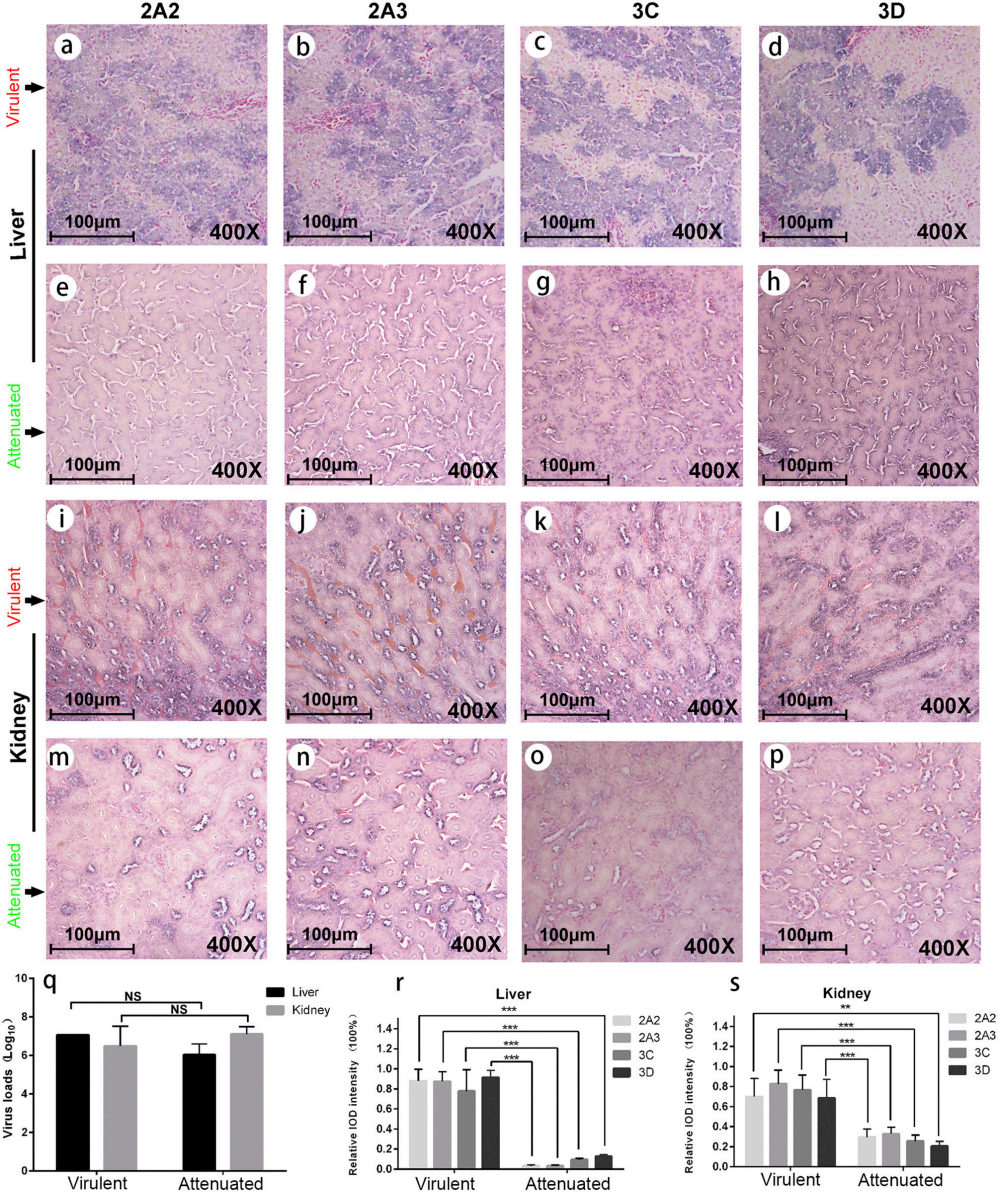
Viral protein expression in virulent and attenuated strains infected liver and kidney. IHC staining of ${{\text{2A}}_{3}}^{\text{pro}}$, ${{\text{2A}}_{3}}^{\text{pro}}$, 3C^pro^, and 3D^pro^ (left to right in each row, respectively) in liver and kidney infected with virulent virus and chicken embryo attenuated virus respectively. The first and second rows display the above four viral protein expressions in liver infected with virulent virus **(a–d)** and attenuated virus **(e-h)**. The third and fourth rows display those four viral protein expressions in kidney infected with virulent virus **(i–l)** and attenuated virus **(m–p)**. The virus loads in both liver and kidney were not significantly different between two groups **(q)**. However, the attenuated strain shows less viral protein expression in duck liver and kidney when compared to a virulent strain **(r,s)**. The data was analyzed by Student *T*-test. **P < 0.01, or ***P < 0.001.

To experimentally verify the impaired expression of viral proteins of the chicken embryo attenuated DHAV-1, we performed infection assay in ducks and evaluated the expression levels of nonstructural proteins in the liver and kidney (Figure 3). We observed significantly lower expression levels of four viral proteins in chicken embryo attenuated strains infected liver and kidney compared to virulent strains infected tissues, despite with similar virus loads (Figures 3A–S). The expression patterns of these viral nonstructural proteins in those two groups were also different, reflecting the stronger invasiveness of the virulent strains in the liver (Figures 3A–H). Additionally, clear steatosis was observed in the virulent virus infected liver but not in chicken embryo attenuated virus infected liver (Figures 3A–H).

## Discussion

**Figure 4:**
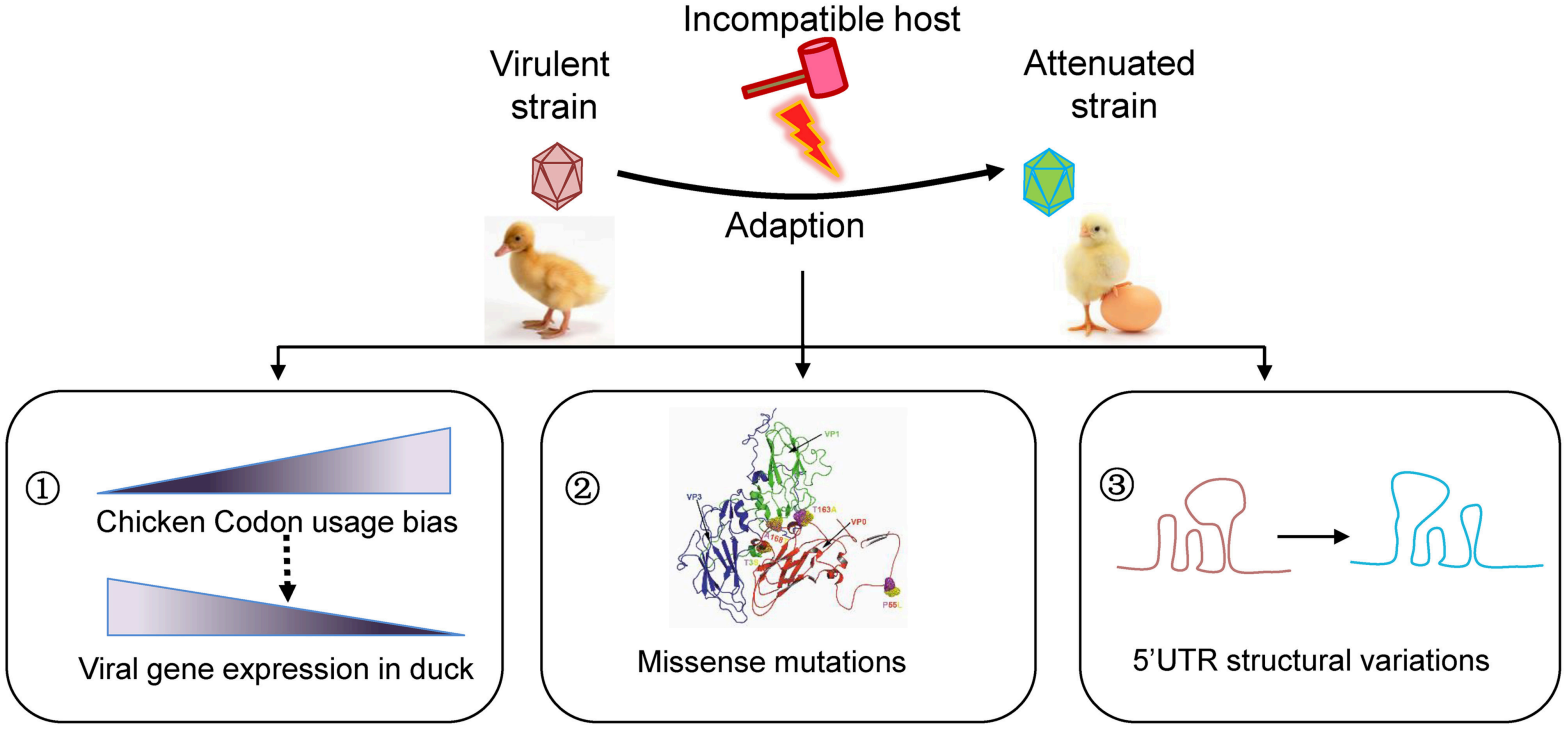
Schematic model for viral attenuation in chicken embryos. Series of viral passaging in chicken embryo is very effective for development of an attenuated vaccine. During the passaging, different types of mutations will occur. Synonymous mutations do not change the amino acid sequences, but they significantly change the codon usage bias that highly regulate the efficiency of gene translation. In fact, codon usage bias of duck virulent strains skewed to the counterparts in chicken after series of passaging, which is essential to increasing viral gene expression in a given host. Of note, the missense mutations in coding region and mutations in untranslated regions may also contribute to viral attenuation of DHAV-1 to some extent. Consequently, the virulence will be attenuated when inoculate to duck, the origin host.

If viruses survived in an unsuitable host, they likely “endure” and then “enjoy” the selections. The remarkable adaptation of some viruses to new hosts is heavily dependent on the generation of *de novo* mutations. The mutation rates vary tremendously among different types of viruses. RNA viruses in particular with a single-stranded genome mutate faster than DNA viruses. As a positive single-stranded RNA virus, the virulent DHAV-1 strains isolated from different geographic regions indeed undergo a convergent evolution driven by passaging in chicken embryos revealed by our phylogenetic analysis. During this process, different types of mutations, including synonymous and non-synonymous mutations in the coding region and mutations in the UTR, have emerged. Conceivably, these mutations coordinately drive the adaptation to the environment (Figure 4).

In respect to the successful development of the live attenuated DHAV-1 vaccine, we believe that the rapid emerging of many synonymous mutations (85.57%) is essential. Although these mutations do not change the amino acid sequence of the viral proteins, they highly regulate the efficiency of gene translation according to codon usage bias. We have previously identified that virulent and attenuated DHAV-1 strains have different codon usage bias (Ou et al., 2017a). In this study, we further revealed a higher correlation between viral codon usage of the attenuated strains with chicken codon usage, compared to the counterpart of virulent strains with duck (Figure 4). The universal and basic driving force is translation, because viral gene expression highly depends on the codon usage bias of the hosts (Ran et al., 2014; Powell and Dion, 2015). Previous studies have also demonstrated that preferred codon frequencies in highly expressed genes correlate with tRNA abundances (Novoa and De Pouplana, 2012; Powell and Dion, 2015). We found that significant more A3s and C3s in attenuated than virulent strains indeed couple with more NNA and NNC codon frequencies in chicken, but the corresponding tRNA copies (tRNA^UNN^ and tRNA^GNN^) were not higher in chicken. A possible explanation could be that tRNA^ANN^ gene copies may have part or indirect impact on codon usage bias.

Importantly, we experimentally confirmed that viral protein expression in the liver and kidney is significantly lower with distinct expression patterns in ducks inoculated with the attenuated strain compared to the inoculation of virulent strain, despite with similar viral titers. Our findings are consistent with previous studies that viral virulence is decreased by the incompatible translation in the process of passages (Ou et al., 2017a) and preferred codon frequencies are correlated with high level of gene expression (Ran et al., 2014; Powell and Dion, 2015).

Besides, the missense mutations in coding region and mutations in UTR may also contribute to viral attenuation of DHAV-1 (Figure 4). The previous study reported that that accumulated amino acid changes in capsid protein of Calicivirus resulted in disappearance of a helix structure, and thus a new phenotype (Nilsson et al., 2003). We observed that the capsid variations are mainly focus on the interface of VP1/0/3, which may lead to a variable spatial organization in viral morphologenisis (Nilsson et al., 2003; Wen et al., 2015). It has been reported that mutations in both 2B protein and 2C protein of hepatitis A virus are involved in adaptation in cell culture, especially in 3889 (Ala-Val), 4087 (Lys-Met), and 4222 (Phe-Ser) (Emerson et al., 1992). However, only one substitute Ala-Val (329, G*C*A-G*T*A) in 2A3 protein was identified in DHAV-1 virulent strains that is very close to 2B protein or 2C protein. While a single surface mutation (Arg-Gly) in NS5B of hepatitis C virus which is similar to 3D polymerase of Picoranvirus increases the replication in cell line (Lohmann et al., 2001). As identified in this study, all these mutations take place at the surface of 3D polymerase (one in palm, one in thumb, two in fingers). These viral-coded proteases interact with other viral proteins or host cell factors, which are vital for viral survival and shunting down host immune responses (Yang et al., 2007; Qu et al., 2011). The 5′UTR of *Picornaviridae* locates an Internal Ribosome Entry Site (IRES), which is vital for viral translation. We found that mutations in 5′UTR but not in 3′UTR cause highly secondary structural variations, which may lead to a translation attenuation due to alterations of stem-loop structures (Ochs et al., 2003; Souii et al., 2013).

Vaccine development is essential in combating infections in human and animals. The live attenuated vaccines developed by series of passages in a given host are widely used since the recent half century (Bhamarapravati and Sutee, 2000; Belshe et al., 2007; Hviid et al., 2008; Song et al., 2013; Minor, 2016). However, developing such a vaccine is laborious and time-consuming. Thus, understanding the mechanisms of viral attenuation will hopefully provide a simple and universal way to vaccine development. In fact, codon deoptimization has been attempted in the development of live attenuated Influenza A and respiratory virus vaccines (Nogales et al., 2014; Cox et al., 2015; Fan et al., 2015; Stobart and Moore, 2015). However, these innovative approaches in vaccine development have been not widely explored and fundamental research is urgently required in parallel to further understand the mechanisms of viral attenuation.

In summary, we have demonstrated that virulent DHAV-1 strains undergo a convergent evolution in the chicken embryos during passaging. Consequently, their codon usage bias skewed to the counterparts in *Gallus gallus*, which is essential to viral attenuation driven by incompatible translation. These knowledge are important for the future development of innovative approaches in designing live attenuated vaccines.

## Ethics statement

The 9-day-old specific pathogen free (SPF) chicken embryos used in passage study were purchased at Merial Company (http://www.merial.com.cn), and this study performed in strict accordance with the recommendations in the ARRIVE guidelines (http://www.nc3rs.org.uk/arrive-guidelines). The animal experiment has been approved by the committee of experiment operational guideline and animal welfare of Sichuan Agricultural University, China (The approved permit number is XF2014-18). All ducks were handled in compliance with the animal welfare regulations and maintained according to standard protocols. All surgeries performed on animals were under sodium pentobarbital anesthesia, and all efforts were made to minimize suffering.

## Author contributions

XO, MW, SM, JC, and AC designed the experiment. XO and SM performed the experiments. XO, JC, and SM wrote the paper. XO, QP, MP, MW, DZ, SC, RJ. SZ, XZ, YL, YY, LZ, and ML proofread the manuscript and data analysis. AC and MW contributed materials and analysis tools. ML, QY, YW, XC, XZ, SZ, and AC contributed to analysis of the experimental data. AC and QP contributed to project supervision. All authors revised it carefully for important intellectual content and approved the manuscript to be published.

### Conflict of interest statement

The authors declare that the research was conducted in the absence of any commercial or financial relationships that could be construed as a potential conflict of interest.
