# Supplementary material for: Incompatible Translation Drives a Convergent Evolution and Viral Attenuation During the Development of Live Attenuated Vaccine
Source: Front Cell Infect Microbiol. 2018 Jul 18;8:249. doi: 10.3389/fcimb.2018.00249 (PMC6058041; doi:10.3389/fcimb.2018.00249)
Supplement: Supplementary file 1 [file Presentation_1.pdf]

Supplementary Material

Incompatible translation drives a convergent evolution and viral attenuation during the development of live attenuated vaccine

Xumin Ou [1,2,3†], Mingshu Wang [1,2,4†], Sai Mao [1,2†], Jingyu Cao [1,2†], Anchun Cheng [1,2,4*], Dekang Zhu [2,4], Shun Chen [1,2,4], Renyong Jia [1,2,4], Mafeng Liu [1,2,4], Qiao Yang [1,2,4], Ying Wu [1,2,4], Xinxin Zhao [1,2,4], Shaqiu Zhang [1,2,4], Yunya Liu [1,2,4], Yanling Yu [1,2,4], Ling Zhang [1,2,4], Xiaoyue Chen [2,4], Maikel P. Peppelenbosch [3] and Qiuwei Pan [3]

1 Institute of Preventive Veterinary Medicine, Sichuan Agricultural University, Chengdu, China,

2 Key Laboratory of Animal Disease and Human Health of Sichuan Province, Sichuan Agricultural University, Chengdu, China,

3 Department of Gastroenterology and Hepatology, Erasmus MC-University Medical Center, Rotterdam, Netherlands,

4 Avian Disease Research Center, College of Veterinary Medicine, Sichuan Agricultural University, Chengdu, China

* Correspondence: chenganchun@vip.163.com

1 Supplementary data

Supplementary data.1 Genomic sequence alignment between virulent and attenuated strains

Supplementary data.2 Protein sequence alignment between virulent and attenuated strains

Supplementary data.3 Constructed viral ORFs (nucleotides and protein) between virulent and attenuated strains

2 Supplementary Figures and Tables

Supplementary Figure 1 Secondary structural variations of 5'UTR and 3'UTR

Supplementary Figure 2 Quaternary structural variations in capsid.

Supplementary Figure 3 Tertiary structural variations in P2-nonstructural proteins

Supplementary Figure 4 Tertiary structural variations in P3 non-structure proteins

Supplementary Table 1 Representative viral strains in this analysis

Supplementary Table 2 Fixed SNPs in the genome of attenuated and virulent strains10

Supplementary Table 3 RSCU of Virulent strains and attenuated strains

Supplementary Table 4 Codon usage bias index of virulent strains and chick embryo attenuated strains

Supplementary Table 5 Comparative analysis of codon usage frequencies and tRNA copies in chickens and ducks

Supplementary data.1 Genomic sequence alignment between virulent and attenuated strains

```
                       *        20         *        40         *        60         *        80         *       100
CH60        : TTTGAAAGC-GGGTGCATGCATGGCCATTTTCCAGCACAGGTTCAGCTGGTATGGCATGCATGCATCGACTGCTTGGGGGTGTCCCTGACCCCCTTAATTCAACG : 104
A66         : TTTGAAAGC-GGGTGCATGCACGGCCATTTTCCAGCACAGGTTCAGCTGGTATGGCATGCATGCATCGACTGCTTGGGGGTGTCCCTGACCCCCTTAATTCAACG : 104
C80         : -TTGAAAGC-GGGTGCATGCATGGCCATTTTCCAGCACAGGTTCAGCTGGTATGGCATGCATGCATCGACTGCTTGGGGGTGTCCCTGACCCCCTTAATTCAACG : 103
MY          : TTTGAAAGC-GGGTGCATGTATGGCCATTTTCCAGCACAGGTTCAGCTGGTATAGCATGCATGCATCGACTGCTTGGGGGTGTCCCTGACCCCCTTAATTCAACG : 104
FC64        : TTTGAAAGC-GGGTGCATGCATGGCCATTTTCCAGCACAGGTTCAGCTGGTATGGCATGCATGCATCGACTGCTTGGGGGTGTCCCTGACCCCCTTAATTCAACG : 104
03D         : ---GAAAGC-GGGTGCATGCATGGCCATTTTCCAGCACAGGTTCAGCTGGTATGGCATGCATGCATCGACTGCTTGGGGGTGTCCCTGACCCCCTTAATTCAACG : 101
ZJ          : ---GAAAGC-GGGTGCATGCATGGCCATTTTCCAGCACAGGTTTAGCTGGTATGGCATGCATACACCGACTGCTTGGGGGTGTCCCTGACCCCCTTAATTCAACG : 101
NA          : TTTGAAAGC-GGGTGCATGCATGGCCATTTTCCAGCACAGGTTTAGCTGGTATGGCATGCATGCACCGACTGCTTGGGGGTGTCCCTGACCCCCTTAATTCAACG : 104
C-XNH       : -TTGAAAGC-GGGTGCATGCATGGCCATTTTCCAGCACAGGTTCAGCTGGTATGGCATGCATGCACTGACTGCTTGGGGGTGTCCCTGACCCCCTTAATTCAACG : 103
LSD/090830  : TTTGAAAGCCGGGTGCATGCATGGCCATTTTCCAGCACAGGTTCAGCTGGTATGGCATGCATGCATCGACTGCTTGGGGGTGTCCCTGACCCCCTTAATTCAACG : 105
FZ99        : -TTGAAAGC-GGGTGCATGCATGGCCATTTTCCAGCACAGGTTCAGCTGGTATGGCATGCGTGCATCGATTGCTTGGGGGTGTCCCTGACCCCCCTAATTCAACG : 103
               ttGAAAGC GGGTGCATGcAtGGCCATTTTCCAGCACAGGTTcAGCTGGTATgGCATGCaTgCA cGAcTGCTTGGGGGTGTCCCTGACCCCCtTAATTCAACG

                  *       120         *       140         *       160         *       180         *       200         *
CH60        : TCTAGCCCACGATACGGGCACCCTATCCAATTTGTTTCCCTTTACCTCCCATGGACTGACTTGTATGGACATGGATCTAAATTGGATTATCCTTTACTTGTGGTG : 209
A66         : TCTAGCCCACGATACGGGCACCCTATCCAATTTGTTTCCCTTTACCTCCCATGGACTGACTTGTATGGACATGGATCTAAATTGGATTATTCTTTACTTGTGGTG : 209
C80         : TCTAGCCCACGATACGGGCACCCTATCCAATTTGTTTCCCTTTACCTCCCATGGACTGACTTGTATGGACATGGATCTAAATTGGATTATCCTTTACTTGTGGTG : 208
MY          : TCTAGCCCACGATACGGGCACCCTATCCAATTTGTTTCCCTTTACCTCCCATGGACTGACTTGTATGGACATGGATCTAAATTGGATTATCCTTTACTTGTGGTG : 209
FC64        : TCTAGCCCACGATACGGGCACCCTATCCAATTTGTTTCCCTTTACCTCCCATGGACTGACTTGTATGGACATGGATCTAAATTGGATTATCCTTTACTTGTGGTG : 209
03D         : TCTAGCCCACGATACGGGCACCCTACCCAATTTGTATCCCTTTACCTCCCATGGATTGACTTGTATGGACTTGGATCTAAATTGGATTATCCTTTACTTGTGGTG : 206
ZJ          : TCTAGCCCACGATACGGGCACCCTATCCAATTTGTATCCCTTTACCTCCCAAGGACTGACTTGTATGGACTTGGATCTAAATTGGATTGTCCTCTACTTGTGGTG : 206
NA          : TCTAGCCCACGATACGGGCACCCTATCCAATTTGTATCCCTTTACCTCCCAAGGACTGACTTGTATGGACTTGGATCTAAATTGGATTGTCCTCTACTTGTGGTG : 209
C-XNH       : TCTAGCCCACGATACGGGCACCCTATCCAATTTGTATCCCTTTACCTCCCAAGGACTGATTTGTATGGACTTGGATCTAAATTGGATTATCCTCTACTTGCGGTG : 208
LSD/090830  : TCTAGCCCACGATACGGGCATCCTATCCAATTTGTATCCCTTTACCTCCCATGGACTGACTTGTATGGACTTGGATCTAAATTGGATTATCCTTTACTTGTGGTG : 210
FZ99        : TCTAGCCCACGATACGGGCACCCTATCCAATTTGTATCCCTTTACCTCCCAAGGACTGACTTGTATGGACTTGGATCTAAATTGGATTGTCCTCTACTTGTGGTG : 208
              TCTAGCCCACGATACGGGCAcCCTAtCCAATTTGT TCCCTTTACCTCCCA GGAcTGAcTTGTATGGAC TGGATCTAAATTGGATT TcCT TACTTGtGGTG

                     220         *       240         *       260         *       280         *       300         *
CH60        : GTTAGCCAACCATGCTCTGGTGTAATGATC-CCATGTGTTCATCTGGCTAAGTGCCGACCTAGGAGGTGGTGCTGAAATATTGCAAGCCACTAGCGTTGTTACAC : 313
A66         : GTTAGCCAACCATGCTCTGGTGTAATGATC-CCATGTGTTCATCTGGCTAAGTGCCGACCTAGGAGGTGGTGCTGAAATATTGCAAGCCACTAGCGTCGTTACAC : 313
C80         : GTTAGCCAACCATGCTCTGGTGTAATGATC-CCATGTGTTCATCTGGCTAAGTGCCGACCTAGGAGGTGGTGCTGAAATATTGCAAGCCACTAGCGTCGTTACAC : 312
MY          : GTTAGCCAACCATGCTCTGGTGTAATGATC-CCATGTGTTCATCTGGCTAAGTGCCGACCTAGGAGGTGGTGCTGAAATATTGCAAGCCACTAGCGTTGTTACAC : 313
FC64        : GTTAGCCAACCATGCTCTGGTGTAATGATC-CCATGTGTTCATCTGGCTAAGTGCCGACCTAGGAGGTGGTGCTGAAATATTGCAAGCCACTAGCGTCGTTACAC : 313
03D         : GTTAGCCAACCATGCTCTGGTGTAATGATT-CCATGTGTTCATCTGGCTAAGTACCGACCTAGGAGGTGGTGCTGAAATATTGCAAGCCACTAGCGTCGTTATGC : 310
ZJ          : GTTAGCCAACCATGCTCTGGTGTAATGATT-CCATGTGTTCGTTTGGCTAAGTGTCGACCTAGGAGGTGGTGCTGAAATATTGCAAGCCACTAGCGTCGTTACGC : 310
NA          : GTTAGCCAACCATGCTCTGGTGTAATGATT-CCATGTATTCGTTTGGCTAAGTGTCGACCTAGGAGGTGGTGCTGAAATATTGCAAGCCACTAGCGTCGTTACGC : 313
C-XNH       : GTTAGCCAACCATGCTCTGGTGTAATGATT-CCATGTGTTCATTTGGCTAGGTGTTGACCTAGGAGGTGGTGCTGAAATATTGCAAGCCACTAGTGTCGTTACAC : 312
LSD/090830  : GTTAGCCAACCATGCTCTGGTGTAATGATT-CCATGTGTTCATCTGGCTAAGTGTCGACCTAGGAGGTGGTGCTGAAATATTGCAAGCCACTAGCGTCATTACGC : 314
FZ99        : GTTAGCCAACCATGCTCTGGTGTAATGATTTCCATGTGTTCCTTTGGCTAAGTGCCGACCTAGGAGGTGGTGCTGAAATATTGCAAGCCACTAGCGTCGTTACGC : 313
              GTTAGCCAACCATGCTCTGGTGTAATGAT  CCATGTgTTC T TGGCTAaGTg cGACCTAGGAGGTGGTGCTGAAATATTGCAAGCCACTAGcGTcgTTAc C

                320         *       340         *       360         *       380         *       400         *       420
CH60        : TTGACCTCTGGATACGTAGGATTAATTGCATTCTGTACAGAGGAAGGCTAGTCTATGCCTTGGGCTAATGGTCTTCGTTGTGAAACGGATTACCGGTAGTAGCAT : 418
A66         : TTGACCTCTGGATACGTAGGATTAATTGCATTCTGTACAGAGGAAGGCTAGTCTATGCCTTGGGCTAATGGTCTTCGTTGTGAAACGGATTACCGGTAGTAGCAT : 418
C80         : TTGACCTCTGGATACGTAGGATTAATTGCATTCTGTACAGAGGAAGGCTAGTCTATGCCTTGGGCTAATGGTCTTCGTTGTGAAACGGATTACCGGTAGTAGCAT : 417
MY          : TCGACCTCTGGATACGTAGGATTAATTGCATTTTGTACAGAGGAAGGCTAGTCTATGCCTTGGGCTAATGGTCTTCGTTGTGAAACGGATTACCGGTAGTAGCAT : 418
FC64        : TTGACCTCTGGATACGTAGGATTAATTGCATTCTGTACAGAGGAAGGCTAGTCTATGCCTTGGGCTAATGGTCTTCGTTGTGAAACGGATTACCGGTAGTAGCAT : 418
03D         : TTAGCCTCTGGATACATGGGATTAATTGCATTCTGTACAGAGGAAGGCTAGTCTATGCCTTGGGCTAATGGTCTTCGTTGTGAAACGGATTACCGGTAGTAGCAT : 415
ZJ          : TTAGCTTCTGGATACGTGGGATTAATTGCATTCTGTACAGAGGAAGGCTAGTCTATGCCTTGGGCTAATGGTCTTCGTTGTGAAACGGATTACCGGTAGTAGCAT : 415
NA          : TTAGCTTCTGGATACGTGGGATTAATTGCATTCTGTACAGAGGAAGGCTAGTCTATGCCTTGGGCTAATGGTCTTCGTTGTGAAACGGATTACCGGTAGTAGCAT : 418
C-XNH       : TTAGCTTCTGGATACGTGGGATTAATTGCATTCTGTACAGAGGAAGGCTAGTCTATGCCTTGGGCTAATGGTCTTCGTTGTGAAACGGATTACCGGTAGTAGCAT : 417
LSD/090830  : TTAGCCCTGGATATGTGGGATCAATTACATTCTGTACAGAGGAAGGCTAGTCTATGCCTTGGTCTAATGGTCTTCGTTGTGAAACGGATTACCGGTAGTAGCAT : 419
FZ99        : TTAACTTCTGGATACGTGGGATTAATTGCATTCTGTACAGAGGAAGGCTAGTCTATGCCTTGGGCTAATGGTCTTCGTTGTGAAACGGATTACCGGTAGTAGCAT : 418
              Tt  C tCTGGATAcgT GGATtAATTgCATTcTGTACAGAGGAAGGCTAGTCTATGCCTTGGgCTAATGGTCTTCGTTGTGAAACGGATTACCGGTAGTAGCAT

                       *       440         *       460         *       480         *       500         *       520
CH60        : CTTAGACTACCAAGGTTGTAGGTGAGTGTGTGGTCTAGAGTAGGCACATATCTGTCCAGGCACATGCACATTGTTGCGATTGCTCCAGACTAGTTCCTGAGGGAC : 523
A66         : CTTAGACTACCAAGGTTGTAGGTGAGTGTGTGGTCTAGAGTAGGCACATATCTGTCCAGGCACATGCACATTGTTGCGATTGCTCCAGACTAGTTCCTGAGGGAC : 523
C80         : CTTAGACTACCAAGGTTGTAGGTGAGTGTGTGGTCTAGAGTAGGCACATATCTGTCCAGGCACATGCACATTGTTGCGATTGCTCCAGACTAGTTCCTGAGGGAC : 522
MY          : CTTAGACTACCAAGGTTGTAGGTGAGTGTGTGGTCTAGAGTAGGCACATATCTGTCCAGGCACATGCACATTGTTGCGATTGCTCCAGACTAGTTCCTGAGGGAC : 523
FC64        : CTTAGACTACCAAGGTTGTAGGTGAGTGTGTGGTCTAGAGTAGGCACATATCTGTCCAGGCACATGCACATTGTTGCGATTGCTCCAGACTAGTTCCTGAGGGAC : 523
03D         : CTTAGACTACCAAGGTTGTAGGTGAGTGTGTGGTCTAGAGTAGGCACACATCTGTCCAGGCACATGCACATTGTTGCGATTGCTCCAGACTAGTTCCTGAGGGAC : 520
ZJ          : CCTAGACTACCAAGGTTGTAGGTGAGTGTGTGGTCTAGAGTAGGCGCACATCTGTCCAGGCACATGCACATTGTTGCGATTGCTCCAGACTAGTTCCTGAGGGAC : 520
NA          : CCTAGACTACCAAGGTTGTAGGTGAGTGTGTGGTCTAGAGTAGGCGCACATCTGTCCAGGCACATGCACATTGTTGCGATTGCTCCAGACTAGTTCCTGAGGGAC : 523
C-XNH       : CCTAGACTACCAAGGTTGTAGGTGAGTGTGTGGTCTAGAGTAGGCACATATCTGTCCAGGCACATGCACATTGTTGTGATTGCTCCAGACTAGTTCCTGAGGGAC : 522
LSD/090830  : CTTAGACTACCAAGGTTGTAGGTGAGTGTGTGGTCTAGAGTAGGCACACATCTATCCAGGCACATGCACATTGTTGTGATTGCTCCAGACTAGTTCCTGAGGGAT : 524
FZ99        : CTTAGACTACCAAGGTTGTAGGTGAGTGTGTGGTCTAGAGTAGGCACACATCTGTCCAGGCACATGCACATTGTTGCGATTGCTCCAGACTAGTTCCTGAGGGAC : 523
              C TAGACTACCAAGGTTGTAGGTGAGTGTGTGGTCTAGAGTAGGCaCA ATCTgTCCAGGCACATGCACATTGTTGcGATTGCTCCAGACTAGTTCCTGAGGGAc

                  *       540         *       560         *       580         *       600         *       620         *
CH60        : AGATGTTGTGGGGGGCTCGGGAAAACCCCCTTAACCTACACTGCCTGATAGGGTCGCGGCTGGTCGAGTCCCACACACTATAATACCAGTAGACTTTCATGAAAT : 628
A66         : AGATGTTGTGGGGGGCTCGGGAAAACCCCCTTAACCTACACTGCCTGATAGGGTCGCGGCTGGTCGAGTCCCACACACTATAATACCAGTAGACTTTCATGAAAT : 628
C80         : AGATGTTGTGGGGGGCTCGGGAAAACCCCCTTAACCTACACTGCCTGATAGGGTCGCGGCTGGTCGAGTCCCACACACTATAATACCAGTAGACTTTCATGAAAT : 627
MY          : AGATGTTGTGGGGGGCTCGGGAAAACCCCCTTAACCTACACTGCCTGATAGGGTCGCGGCTGGTCGAGTCCCACACACTATAATACCAGTAGACTTTCATGAAAT : 628
FC64        : AGATGTTGTGGGGGGCTCGGGAAAACCCCCTTAACCTACACTGCCTGATAGGGTTGCGGCTGGTCGAGTCCCGCACACTATAATACCAGTAGACTTTCATGAAAT : 628
03D         : AGATGTTGTGGGGGGCTTGGGAAAACCCCCTTAACCTACATTGCCTGATAGGGTCGCGGCTGGTCGAGTCCCACACACTATAATACCAGTAGACTTTCATGCAAT : 625
ZJ          : AGATGTTGTGGGGGGCTTGGGAAAACCCCCTTAACCTACACTGCCTGATAGGGTCGCGGCTGGTCGAGTCCCACACACTATAATACCAGTAGACTTTCATGCAAT : 625
NA          : AGATGTTGTGGGGGGCTTGGGAAAACCCCCTTAACCTACACTACCTGATAGGGTCGCGGCTGGTCGAGTCCCACACACTATAATATCAGTAGACTTTCATGCAAT : 628
C-XNH       : AGATGTTGTGGGGGGCTTGGGAAAACCCCCTTAACCTACACTACCTGATAGGGTCGCGGCTGGTCGAGTCCCACACACTATAATATCAGCAGACTTTCATGCAAT : 627
LSD/090830  : AGATGTTGTGGGGGGCTTGGGAAAACCCCCTTAACCTACACTGCCTGATAGGGTCGCGGCTGGTCGAGTCCCACACACTATAATACCAGTAGACTTTCATGCAAT : 629
FZ99        : AGATGTTGTGGGGGGCTTGGGAAAATCCCCTTAACCTACACTACCTGATAGGGTCGCGGCTGGTCGAGTCCCACACACTATAATACCAGTAGACTTTCATGCAAT : 628
              AGATGTTGTGGGGGGCT GGGAAAAcCCCCTTAACCTACAcT CCTGATAGGGTcGCGGCTGGTCGAGTCCCaCACACTATAATAcCAGtAGACTTTCATG AAT

                     640         *       660         *       680         *       700         *       720         *
CH60        : GGATACTCTTACCAAAAATATTGAAGATGCAACAGTCAACATCATTGGATCTTGTGCAGAAAAGGTGGAGGAAGCAATTTCAGGCCTAGGGGCAGTGGAAAGTGT : 733
A66         : GGATACTCTTACCAAAAATATTGAAGATGCAACAGTCAACATCATTGGATCTTGTGCAGAAAAGGTGGAGGAAGCAATTTCAGGCCTAGGGGCAGTGGAAAGTGT : 733
C80         : GGATACTCTTACCAAAAATATTGAAAATGCAACAGTCAACATCATTGGATCTTGTGCAGAAAAGGTGGAGGAAGCAATTTCAGGCCTAGGGGCAGTGGAAAGTGT : 732
MY          : GGATACTCTTACCAAAAATATTGAAGATGCAACAGTCAACATCATTGGATCTTGTGCAGAAAAGGTGGAGGAAGCAATTTCAGGCCTAGGGGCAGTGGAAAGTGT : 733
FC64        : GGATACTCTTACCAAAAATATTGAAGATGCAACAGTCAACATCATTGGATCTTGTGCAGAAAAGGTGGAGGAAGCAATTTCAGGCCTAGGGGCAGTGGAAAGTGT : 733
03D         : GGATACTCTTACCAAAAATATTGAAGATGCAACAGTCAACATCATTGGATCTTGTGCAGAAAAGGTGGAAGAAGCAATTTCAGGCCTAGGGGCAGTGGAAAGTGT : 730
ZJ          : GGATACTCTTACTAAAAACATTGAAGATGCAACAGTTAACATCATTGGATCTTGTGCAGAAAAGGTGGAAGAAGCAATTTCAGGCCTAGGGGCAGTGGAAAGTGT : 730
NA          : GGATACTCTTACTAAAAACATTGAAGATGCAACAGTTAACATCATTGGATCTTGTGCAGAAAAGGTGGAAGAAGCAATTTCAGGCCTAGGGGCAGTGGAAAGTGT : 733
C-XNH       : GGATACTCTTACCAAAAACATTGAAGATGCAACAGTTAACATCATTGGATCTTGTGCAGAAAAGGTGGAAGAAGCAATTTCAGGCCTAGGGGCAGTGGAAAGTGT : 732
LSD/090830  : GGATACTCTTACCAAAAACATTGAAGATGCGACAGTCAACATTATTGGATCTTGTGCGGAAAAGGTGGAGGAAGCAATTTCAGGCCTAGGGGCAGTGGAAAGTGT : 734
FZ99        : GGATACTCTTACCAAAAACATTGAAGATGCAACAGTTAACATCATTGGATCTTGTGCAGAAAAGGTGGAAGAAGCAATTTCAGGCCTAGGGGCAGTGGAAAGTGT : 733
              GGATACTCTTACcAAAAA ATTGAAgATGCaACAGT AACATcATTGGATCTTGTGCaGAAAAGGTGGA GAAGCAATTTCAGGCCTAGGGGCAGTGGAAAGTGT

                740         *       760         *       780         *       800         *       820         *       840
CH60        : GGCATCCACTAATTCAGCCATTGCCACTGCCAATGCAACAACTACACAGACAATACTAGACCCAACGGAGGGTTCTACTGATGATTTCTACTCCTGTTCTTATGA : 838
```

```
A66        : GGCATCCACTAATTCAGCCATTGCCTCTGCCAATGCAACAACTACACAGACAATACTAGACCCAACGGAGGGTTCTACTGATGATTTCTACTCCTGTTCTTATGA :  838
C80        : GGCATCCACTAATTCAGCCATTGCCACTGCCAATGCAACAACTACACAGACAATACTAGACCCAACGGAGGGTTCTACTGATGATTTCTACTCCTGTTCTTATGA :  837
MY         : GGCATCCACTAATTCAGCCATTGCCACTGCCAATGCAACAACTACACAGACAATACTAGACCCAACGGAGGGTTCTACTGATGATTTCTACTCCTGTTCTTATGA :  838
FC64       : GGCATCCACTAATTCAGCCATTGCCACTGCCAATGCAACAACTACACAGACAATACTAGACCCAACGGAGGGTTCTACTGATGATTTCTACTCCTGTTCTTATGA :  838
03D        : GGCATCCACTAATTCAGCCATTGCCACTGCCAATGCAACAACTACACAGACAATACCAGACCCAACGGAGGGTTCCACTGATGATTTCTACTCCTGTTCTTATGA :  835
ZJ         : GGCATCCACTAACTCAGCCATTGCCACTGCCAATGCAACAACTACACAGACAATACCAGACCCAACGGAGGGCTCCACTGATGATTTCTACTCCTGTTCTTATGA :  835
NA         : GGCATCCACTAACTCAGCCATTGCCACTGCCAATGCAACAACTACACAGACAATACCAGACCCAACGGAGGGCTCCACTGATGATTTCTACTCCTGTTCTTATGA :  838
C-XNH      : GGCGTCCACTAACTCAGCCATTGCCACTGCCAATGCAACAACTACACAGACAATACCAGACCCAACAGAGGGCTCCACTGATGATTTCTACTCCTGTTCTTATGA :  837
LSD/090830 : GGCATCCACTAACTCAGCCATTGCCACTGCTAATGCAACAACTACACAGACAATACCAGACCCAACGGAGGGTTCTACTGATGATTTTTACTCCTGTTCTTATGA :  839
FZ99       : GGCATCCACTAACTCAGCCATTGCCACTACCAATGCAACAACTACACAGACAATACCAGACCCAACGGAGGGCTCCACTGATGATTTCTACTCCTGTTCTTATGA :  838
             GGCaTCCACTAA TCAGCCATTGCCaCTgCcAATGCAACAACTACACAGACAATAC AGACCCAACgGAGGG TC ACTGATGATTTcTACTCCTGTTCTTATGA

                     *        860           *        880           *        900           *        920           *        940
CH60       : AGTAGGAGCTCAAGGGGATAATATTTCTAGATTGGTACATCTGGTTACAGGACAGTGGGTTCCAAATGATGATTATTATGCCTGCCTACGCTGGTTAGCAACACC :  943
A66        : AGTAGGAGCTCAAGGGGATAATATTTCTAGATTGGTACATCTGGTTACAGGACAGTGGGTTCCAAATGATGATTATTATGCCTGCCTACGCTGGTTAGCAACACC :  943
C80        : AGTAGGAGCTCAAGGGGATAATATTTCTAGATTGGTACATCTGGTTACAGGACAGTGGGTTCCAAATGATGATTATTATGCCTGCCTACGCTGGTTAGCAACACC :  942
MY         : AGTAGGAGCTCAAGGGGATAATATTTCTAGATTGGTACATCTGGTTACAGGACAGTGGGTTCCAAATGATGATTATTATGCCTGCCTACGCTGGTTAGCAACACC :  943
FC64       : AGTAGGAGCTCAAGGGGATAATATTTCTAGATTGGTACATCTGGTTACAGGACAGTGGGTTCCAAATGATGATTATTATGCCTGCCTACGCTGGTTAGCAACACC :  943
03D        : AGTAGGAGCTCAAGGGGATAACATTTCTAGATTGGTACATCTGGTTACAGGACAGTGGGTTCCAAATGATGATTATTATGCCTGCCTACGCTGGTTAGCAACACC :  940
ZJ         : AGTAGGAGCTCAAGGGGATAACATTTCTAGATTGGTACATCTGGTTACAGGACAGTGGATTCCAAATGATGATTATTATGCCTGCCTACGCTGGTTAGCAACACC :  940
NA         : AGTAGGAGCTCAAGGAGATAACATTTCTAGATTGGTACATCTGGTTACAGGACAGTGGGTTCCAAATGATGATTATTATGCCTGCCTACGCTGGTTAGCAACACC :  943
C-XNH      : AGTAGGAGCTCAAGGAGATAACATTTCTAGATTGGTGCATCTGGTTACAGGACAGTGGATTCCAAATGATGATTATTATGCCTGCCTACGCTGGTTAGCAACACC :  942
LSD/090830 : AGTAGGAGCTCAAGGGGATAACATTTCTAGATTGGTACATCTGGTTACAGGACAGTGGGTTCCAAATGATGATTATTATGTCTGCCTACGCTGGTTAGCAACACC :  944
FZ99       : AGTAGGAGCTCAAGGAGATAATATTTCTAGATTGGTACATCTGGTTACAGGACAGTGGATTCCAAATGATGATTATTATGCCTGCCTACGCTGGTTAGCAACACC :  943
             AGTAGGAGCTCAAGG GATAA ATTTCTAGATTGGTaCATCTGGTTACAGGACAGTGG TTCCAAATGATGATTATTATGcCTGCCTACGCTGGTTAGCAACACC

                 *        960           *        980           *       1000           *       1020           *       1040           *
CH60       : TGCTTGTTTTTTCCAAAATAACACACAACCAGCATATGGCCAGACACGATATTTTAGGTTTATCAGATGTGGCTTCCATTTTAGGTTGCTTGTGAATGCCCCCTC : 1048
A66        : TGCTTGTTTTTTCCAAAATAACACACAACCAGCATATGGCCAGACACGATATTTTAGGTTTATCAGATGTGGCTTCCATTTTAGGTTGCTTGTGAATGCCCCCTC : 1048
C80        : TGCTTGTTTTTTCCAAAATAACACACAACCAGCATATGGCCAGACACGATATTTTAGGTTTATCAGATGTGGCTTCCATTTTAGGTTGCTTGTGAATGCCCCCTC : 1047
MY         : TGCTTGTTTTTTCCAAAATAACACACAACCAGCATATGGCCAGACACGATATTTTAGGTTTATCAGATGTGGCTTCCATTTTAGGTTGCTTGTGAATGCCCCCTC : 1048
FC64       : TGCTTGTTTTTTCCAAAATAACACACAACCAGCATATGGCCAGACACGATATTTTAGGTTTATCAGATGTGGCTTCCATTTTAGGTTGCTTGTGAATGCCCCCTC : 1048
03D        : TGCTTGTTTTTTCCAAAATAACACACAACCAGCTTATGGCCAGACACGATATTTTAGGTTTATCAGATGTGGCTTCCATTTTAGGTTGCTTGTAAATGCCCCCTC : 1045
ZJ         : TGCTTGCTTTTTTCAAAATAACACACAACCAGCTTATGGCCAGACACGATATTTTAGGTTTATCAGATGTGGCTTCCATTTTAGGTTGCTTGTGAATGCCCCCTC : 1045
NA         : TGCTTGCTTTTTTCAAAATAACACACAACCAGCTTATGGCCAGACACGATATTTTAGGTTTATCAGATGTGGCTTCCATTTTAGGTTGCTTGTGAATGCCCCCTC : 1048
C-XNH      : TGCCTGCTTTTTTCAAAATAATACACAACCAGCTTATGGCCAGACACGATATTTTAGGTTTATTAGATGTGGCTTCCATTTTAGGTTGCTTGTGAATGCCCCCTC : 1047
LSD/090830 : TGCTTGTTTTTTCCAAAACAACACACAACCAGCTTATGGCCAGACACGATATTTTAGGTTTATCAGATGTGGCTTCCATTTTAGGTTGCTTGTGAACGCCCCCTC : 1049
FZ99       : TGCTTGCTTTTTTCAAAATAACACACAACCAGCTTATGGCCAGACTCGATATTTTAGGTTTATCAGATGTGGCTTCCATTTTAGGTTGCTCGTGAATGCCCCCTC : 1048
             TGCtTG TTTTT CAAAAtAAcACACAACCAGC TATGGCCAGACaCGATATTTTAGGTTTATcAGATGTGGCTTCCATTTTAGGTTGCTtGTgAAtGCCCCCTC

                   1060           *       1080           *       1100           *       1120           *       1140           *
CH60       : TGGCTCTGCTGGAGCACTTATGCTAGTTTGGATGCCCTACCCCTATTGTCGGGTCTTATCTGGTGCTAATCAGATCCATACAAATGTTGAGAGAAGGAGTCTAAT : 1153
A66        : TGGCTCTGCTGGAGCACTTATGCTAGTTTGGATGCCCTACCCCTATTGTCGGGTCTTATCTGGTGCTAATCAGATCCATACAAATGTTGAGAGAAGGAGTCTAAT : 1153
C80        : TGGCTCTGCTGGAGCACTTATGCTAGTTTGGATGCCCTACCCCTATTGTCGGGTCTTATCTGGTGCTAATCAGATCCATACAAATGTTGAGAGAAGGAGTCTAAT : 1152
MY         : TGGCTCTGCTGGAGCACTTATGCTAGTTTGGATGCCCTACCCCTATTGTCGGGTCTTATCTGGTGCTAATCAGATCCATACAAATGTTGAGAGAAGGAGTCTAAT : 1153
FC64       : TGGCTCTGCTGGAGCACTTATGCTAGTTTGGATGCCCTACCCCTATTGTCGGGTCTTATCTGGTGATAATCAGATCCATACAAATGTTGAGAGAAGGAGTCTAAT : 1153
03D        : TGGCTCTGCTGGAGCACTTATGTTAGTTTGGATGCCCTACCCCTATTGTCGGGTCTTATCTGGTACTAATCAGATCCATGCAAATGTTGAGAGAAGGAGTCTAAT : 1150
ZJ         : TGGCTCTGCTGGAGCGCTTATGCTAGTTTGGATGCCCTACCCCTATTGTCGGGTTTTATCTGGTACTAATCAGATCCATGTAAATGTTGAAAGAAGGAGTCTAAT : 1150
NA         : TGGCTCTGCTGGAGCGCTTATGCTAGTTTGGATGCCCTACCCCTATTGTCGGGTTTTATCTGGTACTAATCAGATCCATGCAAATGTTGAAAGAAGGAGTCTAAT : 1153
C-XNH      : TGGCTCTGCTGGAGCACTTATGCTAGTTTGGATGCCCTACCCCTATTGTCGGGTTTTATCTGGTTCTAATCAGATTCATGCAAATGTTGAGAGAAGGAGTCTAAT : 1152
LSD/090830 : TGGCTCTGCTGGAGCACTTATGTTAGTTTGGATGCCCTACCCTTATTGTCGGGTTTTATCTGGTACTAATCAGATTCATGCAAATGTTGAGAGGAGGAGTCTAAT : 1154
FZ99       : TGGCTCTGCTGGAGCACTTATGTTAGTTTGGATGCCCTACCCCTATTGTCGGGTTTTATCTGGTACTAATCAGATTCATGCAAATGTTGAGAGAAGGAGTCTAAT : 1153
             TGGCTCTGCTGGAGCaCTTATG TAGTTTGGATGCCCTACCCcTATTGTCGGGT TTATCTGGT cTAATCAGAT CAT cAAATGTTGAgAGAaGGAGTCTAAT

                 1160           *       1180           *       1200           *       1220           *       1240           *       1260
CH60       : GAACCTGCCCTATGCCATCTTGGATCTCCGCACCAACACAGAAATTGACCTTGTAGTTCCATATGTCAACTACCGGAACTATGTAGAGATTACAACCAGTGATAC : 1258
A66        : GAACCTGCCCTATGCCATCTTGGATCTCCGCACCAACACAGAAATTGACCTTGTAGTTCCATATGTCAACTACCGGAACTATGTAGAGATTACAACCAGTGATAC : 1258
C80        : GAACCTGCCCTATGCCATCTTGGATCTCCGCACCAACACAGAAATTGACCTTGTAGTTCCATATGTCAACTACCGGAACTACGTAGAGATTACAACCAGTGATAC : 1257
MY         : GAACCTGCCCTATGCCATCTTGGATCTCCGCACCAACACAGAAATTGACCTTGTAGTTCCATATGTCAACTACCGGAACTATGTAGAGATTACAACCAGTGATAC : 1258
FC64       : GAACCTGCCCTATGCCATCTTGGATCTCCGCACCAACACAGAAATTGACCTTGTAGTTCCATATGTCAACTACCGGAACTATGTAGAGATTACAACCAGTGATAC : 1258
03D        : GAACCTGCCTTATGCCATCTTGGATCTCCGCACCAATACAGAAATTGACCTTGTGGTTCCATATGTCAACTACCGGAACTATGTGGAGATTACAACCAGTGATAC : 1255
ZJ         : GAACCTGCCTTATGCCATCTTGGATCTTCGCACCAACACAGAAATTGACCTTGTGGTTCCATATGTAAATTACCGAAACTATGTGGAGATTACAACCAGTGACGC : 1255
NA         : GAACCTGCCTTATGCCATCTTGGATCTTCGCACCAACACAGAAATTGACCTTGTGGTTCCATATGTAAATTACCGAAACTATGTGGAGATTACAACCAGTGACGC : 1258
C-XNH      : GAACCTACCTTATGCCATCTTGGATCTCCGCACCAACACAGAAATTGACCTTGTGGTTCCATATGTCAACTACCGAAACTATGTGGAGATTACAACCAGTGACAC : 1257
LSD/090830 : GAACCTGCCTTATGCCATCTTGGATCTCCGCACCAACACAGAAATTGACCTTGTGGTTCCATATGTCAACTACCGGAACTATGTGGAGATTACAACCAGTGACAC : 1259
FZ99       : GAACCTGCCTTATGCCATCTTGGATCTCCGCGCTAACACAGAAATTGACCTTGTGGTTCCATATGTCAACTACCGAAACTATGTGGAGATTACAACCAGTGACGC : 1258
             GAACCTgCC TATGCCATCTTGGATCTcCGCaCcAAcACAGAAATTGACCTTGT GTTCCATATGTcAAcTACCG AACTAtGT GAGATTACAACCAGTGA  C

                     *       1280           *       1300           *       1320           *       1340           *       1360
CH60       : AACTGGTGGTGCCATCTGTGTCATTGTGTTAGGCAAGTACCGACATGGCAATGGAACCTCCAACACTGTAGATTTCACATTATTTGGAGAACTCCTTGAAACTGA : 1363
A66        : AACTGGTGGTGCCATCTGTGTCATTGTGTTAGGCAAGTACCGACATGGCAATGGAACCTCCAACACTGTAGATTTCACATTATTTGGAGAACTCCTTGAAACTGA : 1363
C80        : AACTGGTGGTGCCATCTGTGTCATTGTGTTAGGCAAGTACCGACATGGCAATGGAACCTCCAACACTGTAGATTTCACATTATTTGGAGAACTCCTTGAAACTGA : 1362
MY         : AACTGGTGGTGCCATCTGTGTCATTGTGTTAGGCAAGTACCGACATGGCAATGGAACCTCCAACACTGTAGATTTCACATTATTTGGAGAACTCCTTGAAACTGA : 1363
FC64       : AACTGGTGGTGCCATCTGTGTCATTGTGTTAGGCAAGTACCGACATGGCAATGGAACCTCCAACACTGTAGATTTCACATTATTTGGAGAACTCCTTGAAACTGA : 1363
03D        : AACTGGTGGTGCTATCTGTGTGGTTGTGCTAGGCAAGTACCGACATGGCAATGGAACCTCCAACACTGTAGATTTCACATTATTTGGAGAACTCCTTGAAACTGA : 1360
ZJ         : AACTGGTGGTGCTATCTGTGTGATTGTGCTAGGCAAGTACCGGCATGGCAATGGAACCTCCAACACTGTGGATTTCACATTATTTGGAGAACTCCTTGAAACTGA : 1360
NA         : AACTGGTGGTGCTATCTGTGTGATTGTGCTAGGCAAGTACCAGCATGGCAATGGAACCTCCAACACTGTGGATTTCACATTATTTGGAGAACTCCTTGAAACTGA : 1363
C-XNH      : AACTGGTGGTGCTATCTGTGTGATTGTGCTAGGCAAATACCGGCATGGCAATGGAACCTCCAATACTGTGGATTTCACATTATTTGGAGAACTCCTTGAAACTGA : 1362
LSD/090830 : AACTGGTGGTGCTATCTGTGTGATTGTGCTAGGCAAGTACCGACATGGCAATGGAACTTCCAATACTGTAGATTTCACATTATTTGGAGAACTCCTTGAAACTGA : 1364
FZ99       : AACTGGTGGTGCTATCTGTGTGATTGTGCTAGGCAAGTACCGGCATGGCAATGGAACCTCCAACACTGTGGATTTCACATTATTTGGAGAACTCCTTGAAACTGA : 1363
             AACTGGTGGTGC ATCTGTGT aTTGTG TAGGCAAgTACCg CATGGCAATGGAACcTCCAAcACTGT GATTTCACATTATTTGGAGAACTCCTTGAAACTGA

                 *       1380           *       1400           *       1420           *       1440           *       1460           *
CH60       : TTTGCAGTGTCCCCGACCATTTGACAATCAGGGAAAGAGAAAACCATGCAGGAGGCCCATTCACAAACCCAAGAATCCACCACAAGAGCCAAGGGTCATCATTCA : 1468
A66        : TTTGCAGTGTCCCCGACCATTTGACAATCAGGGAAAGAGAAAACCATGCAGGAGGCCCATTCACAAACCCAAGAATCCACCACAAGAGCCAAGGGTCATCATTCA : 1468
C80        : TTTGCAGTGTCCCCGACCATTTGACAATCAGGGAAAGAGAAAACCATGCAGGAGGCCCATTCACAAACCCAAGAATCCACCACAAGAGCCAAGGGTCATCATTCA : 1467
MY         : TTTGCAGTGTCCCCGACCATTTGACAATCAGGGAAAGAGAAAACCATGCAGGAGGCCCATTCACAAACCCAAGAATCCACCACAAGAGCCAAGGGTCATCATTCA : 1468
FC64       : TTTGCAGTGTCCCCGACCATTTGACAATCAGGGAAAGAGAAAACCACGCAGGAGGCCCATTCACAAACCCAAGAATCCACCACAAGAGCCAAGGGTCATCATTCA : 1468
03D        : TTTGCAATGTCCCCGACCATTCGACAATCAGGGAAAGAGAAAACCACGCAGGAGGCCCATTCACAAACCCAAGAATCCACCACAGGAGCCAAGAGTCATCATTCA : 1465
ZJ         : TTTGCAGTGTCCCCGACCATTCGACAATCAGGGAAAGAGAAAACCACGCAGGAGGCCCATTCACAATCCCAAGAATCCACCACAGGAGCCAAGAGTCATTATTCA : 1465
NA         : TTTGCAGTGTCCCCGACCATTCGACAATCAGGGAAAGAGAAAACCACGCAGGAGGCCCATTCACAATCCCAAGAATCCACCACAGGAGCCAAGAGTCATTATTCA : 1468
C-XNH      : TTTGCAGTGTCCCCGACCATTCGACAATCAGGGAAAGAGAAAACCACGCAGGAGGCCCATTCACAAACCTAAGAATCCGCCACAGGAGCCAAGAGTCATCATTCA : 1467
LSD/090830 : TTTGCAGTGTCCCCGACCATTCGACAATCAGGGAAAGAGAAAACCACGCAGGAGGCCCATTCATAAACCCAAGAATCCACCACAGGAGCCAAGAGTCATCATTCA : 1469
FZ99       : TTTGCAGTGTCCCCGACCATTCGATAATCAGGGAAAGAGAAAACCACGCAGGAGGCCCATTCACAAACCCAAGAATCCGCCACAGGAGCCAAGAGTCATCATTCA : 1468
             TTTGCAgTGTCCCCGACCATT GAcAATCAGGGAAAGAGAAAACCA GCAGGAGGCCCATTCAcAAaCCcAAGAATCCaCCACA GAGCCAAG GTCATcATTCA

                   1480           *       1500           *       1520           *       1540           *       1560           *
CH60       : ACCAGGACCTGGGGCAGCAAACCTTTCAAATTCTAGTGTTATAGCAATGGCTGAAAGCACAGCACTTGCTAATGAAGGGACAGCTGTTGACTATTCCACAGCTGG : 1573
A66        : ACCAGGACCTGGGGCAGCAAACCTTTCAAATTCTAGTGTTATAGCAATGGCTGAAAGCACAGCACTTGCTAATGAAGGGACAGCTGTTGACTATTCCACAGCTGG : 1573
C80        : ACCAGGACCTGGGGCAGCAAACCTTTCAAATTCTAGTGTTATAGCAATGGCTGAAAGCACAGCACTTGCTAATGAAGGGACAGCTGTTGACTATTCCACAGCTGG : 1572
MY         : ACCAGGACCTGGGGCAGCAAACCTTTCAAATTCTAGTGTTATAGCAATGGCTGAAAGCACAGCACTTGCTAATGAAGGGACAGCTGTTGACTATTCCACAGCTGG : 1573
```

```
FC64       : ACCAGGACCTGGGGCAGCAAACCTTTCAAATTCTAGTGCTATAGCAATGGCTGAAAGCACAGCACTTGCTAATGAAGGGACAGCTGTTGACTATTCCATAGCTGG : 1573
03D        : ACCAGGACCTGGGGCAGCAAACCTTTCAAACTCTAGTGTTATAGCAATGGCTGAAAGCACAGCACTTGCCAATGAAGGGACAGCTGTTGACTATTCCACAGCCGG : 1570
ZJ         : ACCAGGACCTGGGGCAGCAAACCTTTCAAATTCTAGTGTTATAGCAATGGCTGAAAGCACAGCACTTGCCAATGAAGGGACAGCTGTTGACTATTCTACAGCCGG : 1570
NA         : ACCAGGACCTGGGGCAGCAAACCTTTCAAATTCTAGTGTTATAGCAATGGCTGAAAGCACAGCACTTGCCAATGAAGGGACAGCTGTTGACTATTCTACAGCCGG : 1573
C-XNH      : ACCAGGACCTGGGGCAGCAAACCTTTCAAATTCTAGTGTTATAGCAATGGCTGAAAGCACAGCACATAGCCAATGAAGGGACAGCTGTTGACTATTCCACAGCCGG : 1572
LSD/090830 : ACCAGGACCTGGAGCAGCAAACCTTTCAAACTCTAGTGTTATAGCGATGGCTGAAAGCACAGCACTTGCTAATGAAGGGACAGCTGTTGACTATTCCACAGCCGG : 1574
FZ99       : ACCAGGACCTGGGGCAGCAAACCTTTCAAACTCTAGTGTTATAGCAATGGCTGAAAGCACAGCACTTGCTAATGAAGGGACAGCTGTTGACTATTCTACAGCCGG : 1573
             ACCAGGACCTGGgGCAGCAAACCTTTCAAA TCTAGTGtTATAGCaATGGCTGAAAGCACAGCACTtGC AATGAAGGGACAGCTGTTGACTATTC AcAGC GG

               1580         *       1600         *       1620         *       1640         *       1660         *       1680
CH60       : ATGTGCATCATCTGTTGATGATGTAATTATGGTCCTGCGCCGATGGCAGATTCTAGCAAGTTTCCAGTGGCAGTCAACCCTAACTCCCAGTGCTCGCATTAATCG : 1678
A66        : ATGTGCATCATCTGTTGATGATGTAATTATGGTCCTGCGCCGATGGCAGATTCCAGCAAGTTTCCAGTGGCAGTCAACCCTAACTCCCAATGCTCGCATTAATCG : 1678
C80        : ATGTGCATCATCTGTTGATGATGTAATTATGGTCCTGCGCCGATGGCAGATTCTAGCAAGTTTCCAGTGGCAGTCAACCCTAACTCCCAATGCTCGCATTAATCG : 1677
MY         : ATGTGCATCATCTGTTGATGATGTAATTATGGTCCTGCGCCGATGGCAGATTCTAGCAAGTTTCCAGTGGCAGTCAACCCTAACTCCCAGTGATCGCATTAATCG : 1678
FC64       : ATGTGCATCATCTGTTGATGATGTAATTATGGTCCTGCGCCGATGGCAGATTCTAGCAAGTTTCCAGTGGCAGTCAACCCTAACTCCCAGTGCTCGCATTAATCG : 1678
03D        : ATGTGCATCATCTGTTGATGATGTAATTATGGTCCTGCGCCGATGGCAGATTCTAGCAAGTTTCCAGTGGCAGTCAACCCTAACTCCCAGTGTTCGCATTAATCG : 1675
ZJ         : GTGTGCGTCATCTGTTGATGATGTAATTATGGTCCTGCGCCGATGGCAGATTCTAGCAAGTTTCCAGTGGCAGTCAACTCAAACCCCCAGTACTCGCATTAATCG : 1675
NA         : GTGTGCGTCATCTGTTGATGATGTAATTATGGTCCTGCGCCGATGGCAGATTCTAGCAAGTTTCCAGTGGCAGTCAACTCAAACCCCCAGTACTCGCATTAATCG : 1678
C-XNH      : GTGTGCATCATCTGTTGATGATGTAATTATGGTCCTGCGCCGATGGCAGATTCTAGCAAGCTTCCAGTGGCAGTCAACCCAAACCCCCAGTACTCGCATTAATCG : 1677
LSD/090830 : ATGTGCATCATCTGTTGATGATGTAATTATGGTCCTGCGCCGATGGCAGATTTTAGCAAGTTTCCAGTGGCAGTCAACCTTAACTCCCAGTGCTCGCATTAATCG : 1679
FZ99       : GTGTGCGTCATCTGTTGATGATGTAATTATGGTCCTGCGCCGATGGCAGATTCTAGCAAGTTTCCAGTGGCAGTCAACCCAAACCCCCAGTACTCGCATTAATCG : 1678
              TGTGC TCATCTGTTGATGATGTAATTATGGTCCTGCGCCGATGGCAGATTctAGCAAGtTTCCAGTGGCAGTCAACcc AAC CCCAgT cTCGCATTAATCG

                         *       1700         *       1720         *       1740         *       1760         *       1780
CH60       : CTATCAATTGATTTTCAGAAACATCCCAACATTTTCTCTTTTTTTTGACAAATTTCAGTACTGGAGAGGGTCATTGGAGGTTAAATTTATGACATTTGGCAGCCA : 1783
A66        : CTATCAATTGATTTTCAGAAACATCCCAACATTTTCTCTCTTTTTTGACAAATTTCAGTACTGGAGAGGGTCATTGGAGGTTAAATTTATGACATTTGGCAGCCA : 1783
C80        : CTATCAATTGATTTTCAGAAACATCCCAACATTTTCTCTTTTTTTTGACAAATTTCAGTACTGGAGAGGGTCATTGGAGGTTAAATTTATGACATTTGGCAGCCA : 1782
MY         : CTATCAATTGATTTTCAGAAACATCCCAACATTTTCTCTTTTTTTTGACAAATTTCAGTACTGGAGAGGGTCATTGGAGGTTAAATTTATGACATTTGGCAGCCA : 1783
FC64       : CTATCAATTGATTTTCAGAAACATCCCAACATTTTCTCTTTTTTTTGACAAATTTCAGTACTGGAGAGGGTCATTGGAGGTTAAATTTATGACATTTGGCAGCCA : 1783
03D        : CTATCAATTGATTTTCAGAAACATCCCAACATTCTCTCTCTTCTTTGACAAATTTCAGTACTGGAGAGGGTCATTGGAGGTTAAATTCATGACATTTGGCAGCCA : 1780
ZJ         : CTATCTATTGATTTTTAGAAACATTCCAACATTTTCCCTCTTCTTTGATAAATTTCAGTACTGGAGAGGGTCATTGGAGGTTAAATTCATGACATTTGGCAGCCA : 1780
NA         : CTATCAATTGATTTTTAGAAACATTCCAACATTTTCCCTCTTCTTTGATAAATTTCAGTACTGGAGAGGGTCATTGGAGGTTAAATTCATGACATTTGGCAGCCA : 1783
C-XNH      : CTATCAATTGATTTTTAGAAATATTCCAACATTTTCCCTTTTCTTTGATAAATTTCAGTACTGGAGAGGGTCATTGGAGGTTAAATTCATGACATTTGGCAGCCA : 1782
LSD/090830 : TTACCAATTGATTTTCAGAAACATTCCAACATTCTCTCTCTTCTTTGACAAATTTCAGTACTGGAGAGGGTCATTGGAGGTTAAATTCATGACATTTGGCAGCCA : 1784
FZ99       : CTATCAATTGATTTTTAGAAACATTCCAACATTCACCCTCTTCTTTGATAAATTTCAGTACTGGAGAGGGTCATTGGAGGTTAAATTCATGACATTTGGCAGCCA : 1783
             cTAtCaATTGATTTT AGAAAcAT CCAACATT tC CT TT TTTGA AAATTTCAGTACTGGAGAGGGTCATTGGAGGTTAAATT ATGACATTTGGCAGCCA

                  *       1800         *       1820         *       1840         *       1860         *       1880         *
CH60       : ATTTAACACTGGGAGATATCAAATGTCTTGGTATCCAATTGCTGATGGTGAACAGAGCTTGGCACAATGCCAGAATTCGGTATTTGTAACTGGGGATGTGTGTGC : 1888
A66        : ATTTAACACTGGGAGATATCAAATGTCTTGGTATCCAATTGCTGATGGTGAACAGAGCTTGGCACAATGCCAGAATTCGGTATTTGTAACTGGGGATGTGTGTGC : 1888
C80        : ATTTAACACTGGGAGATATCAAATGTCTTGGTATCCAATTGCTGATGGTGAACAGAGCTTGGCACAATGCCAGAATTCGGTATTTGTAACTGGGGATGTGTGTGC : 1887
MY         : ATTTAACACTGGGAGATATCAAATGTCTTGGTATCCAATTGCTGATGGTGAACAGAGCTTGGCACAATGCCAGAATTCGGTATTTGTAACTGGGGATGTGTGTGC : 1888
FC64       : ATTTAACACTGGGAGATATCAAATGTCTTGGTATCCAATTGCTGATGGTGAACAGAGCTTGGCACAATGCCAGAATTCGGTATTTGTAACTGGGGATGTGTGTGC : 1888
03D        : ATTCAACACTGGGAGATATCAAATGTCTTGGTATCCAATTGCTGATGGTGAACAGAGCTTGGCGCAATGCCAGAATTCGGTGTTTGTAACTGGGGATGTGTGTGC : 1885
ZJ         : ATTCAATACTGGGAGATATCAAATGTCTTGGTATCCAATTGCTGATGGTGAACAGAGTTTGGCACAATGCCAGAATTCGGTGTTTGTAACTGGGGATGTGTGTGC : 1885
NA         : ATTCAATACTGGGAGATATCAAATGTCTTGGTATCCAATTGCTGATGGTGAACAGAGCTTGGCACAATGCCAGAATTCGGTGTTTGTAACTGGGGATGTGTGTGC : 1888
C-XNH      : ATTCAATACTGGGAGATATCAAATGTCTTGGTATCCAATTGCTGATGGTGAACAGAGCTTGGCGCAATGCCAGAATTCAGTGTTTGTAACTGGGGATGTGTGTGC : 1887
LSD/090830 : ATTCAACACTGGGAGATATCAAATGTCTTGGTATCCAATTGCTGATGGTGAACAGAGCTTGGCGCAATGCCAGAATTCGGTGTTTGTGACTGGGGATGTGTGTGC : 1889
FZ99       : ATTCAATACTGGGAGATATCAAATGTCTTGGTATCCAATTGCTGATGGTGAACAGAGCTTGGCACAATGCCAGAATTCGGTGTTTGTAACTGGGGATGTGTGTGC : 1888
             ATT AA ACTGGGAGATATCAAATGTCTTGGTATCCAATTGCTGATGGTGAACAGAGcTTGGC CAATGCCAGAATTCgGT TTTGTaACTGGGGATGTGTGTGC

                    1900         *       1920         *       1940         *       1960         *       1980         *
CH60       : CACACCTGTGACCCTAACTCTGCCATTTACATCAACCACCTGGCGCAAAAGCAGGAGAGACCCTTACGGGTATTTGATGTGGCATGTTGTCAATCGACTCACTGT : 1993
A66        : CACACCTGTGACCCTAACTCTGCCATTCACATCAACCACCTGGCGCAAAAGCAGGAGAGACCCTTACGGGTATTTGATGTGGCATGTTGTCAATCGACTCACTGT : 1993
C80        : CACACCTGTGACCCTAACTCTGCCATTTACATCAACCACCTGGCGCAAAAGCAGGAGAGACCCTTACGGGTATTTGATGTGGCATGTTGTCAATCGACTCACTGT : 1992
MY         : CACACCTGTGACCCTAACTCTGCCATTTACATCAACCACCTGGCGCAAAAGCAGGAGAGACCCTTACGGGTATTTGATGTGGCATGTTGTCAATCGACTCACTGT : 1993
FC64       : CACACCTGTGACCCTAACTCTGCCATTTACATCAACCACCTGGCGCAAAAGCAGGAGAGACCCTTACGGGTATTTGATGTGGCATGTTGTCAATCGACTCACTGT : 1993
03D        : TACACCCGTGACCCTAACTCTGCCATTTACATCAACCACCTGGCGTAAAAGCACAAGAGACCCTTACGGGTATTTGATGTGGCATGTTGTCAATCGACTCACTGT : 1990
ZJ         : CACACCCGTGACCCTAACTCTGCCATTTACATCAACCACCTGGCGCAAAAGCACAAGAGACCCTTACGGGTATTTGATGTGGCATGTTGTCAATCGACTCACCGT : 1990
NA         : CACACCCGTGACCCTAACTCTGCCATTTACATCAACCACCTGGCGCAAAAGCACAAGAGACCCTTACGGGTATTTGATGTGGCATGTTGTTAATCGACTCACTGT : 1993
C-XNH      : CACACCCGTGACCCTAACTTTGCCATTTACATCAACCACCTGGCGCAAAAGCACAAGAGACCCTTACGGGTATTTGATGTGGCACGTTGTCAATCGACTCACTGT : 1992
LSD/090830 : CACACCCGTGACTCTAACTCTGCCATTTACATCAACCACCTGGCGCAAAAGTACAAGAGACCCTTACGGGTATTTGATGTGGCATGTTGTCAATCGACTTACTGT : 1994
FZ99       : CACACCCGTGACCCTAACTCTGCCATTTACATCAACCACCTGGCGCAAAAGCACAAGAGACCCTTACGGGTATTTGATGTGGCATGTTGTCAATCGACTCACTGT : 1993
             cACACC GTGACcCTAACTcTGCCATTtACATCAACCACCTGGCGcAAAAGcAC AGAGACCCTTACGGGTATTTGATGTGGCAtGTTGTcAATCGACTcAcTGT

               2000         *       2020         *       2040         *       2060         *       2080         *       2100
CH60       : GAATTCATCAGCCCCATCCAATATTGACTGCACAGTGTTGCTCCGCGTGGGCAAAGATTTTCAGTTTACCGCCCCACTCTATGGAAATTTGCAGATGGCAACTAA : 2098
A66        : GAATTCATCAGCCCCATCCAATATTGACTGCACAGTGTTGCTCCGCGTGGGCAAAGATTTTCAGTTTACCGCCCCACTCTATGGAAATTTGCAGATGGCAACTAA : 2098
C80        : GAATTCATCAGCCCCATCCAATATTGACTGCACAGTGTTGCTCCGCGTGGGCAAAGATTTTCAGTTTACCGCCCCACTCTATGGAAATCTGCAGATGGCAACTAA : 2097
MY         : GAATTCATCAGCCCCATCCAATATTGACTGCACAGTGTTGCTCCGCGTGGGCAAAGATTTTCAGTTTACCGCCCCACTCTATGGAAATTTGCAGATGGCAACTAA : 2098
FC64       : GAATTCATCAGCCCCATCCAATATTGACTGCACAGTGTTGCTCCGCGTGGGCAAAGATTTTCAGTTTACCGCCCCACTCTATGGAAATTTGCAGATGGCAACTAA : 2098
03D        : GAATTCATCAGCCCCATCCAATATTGACTGCACAGTGTTGCTCCGTGTGGGCAAAGATTTTCAGTTTACTGCCCCACTCTATGGAAATTTGCAGATGGCAACCAA : 2095
ZJ         : GAACTCATCAGCCCCATCCAATATTGACTGCACGGTGTTGCTCCGTGTGGGTAAAGATTTTCAGTTCACTGCCCCACTTTATGGCAATCTGCAGATGGCAACCAA : 2095
NA         : GAATTCATCAGCCCCATCCAATATTGACTGCACGGTGTTGCTCCGTGTGGGTAAAGATTTTCAGTTCACTGCCCCACTTTATGGCAATTTGCAGATGGCAACCAA : 2098
C-XNH      : GAATTCATCAGCCCCATCCAATATTGACTGCACGGTGTTGCTCCGTGTGGGTAAAGATTTTCAGTTTACCGCCCCACTCTATGGCAATTTGCAGATGGCAACCAA : 2097
LSD/090830 : GAATTCATCAGCCCCATCCAATATTGACTGCACAGTGTTGCTCCGTGTGGGCAAAGATTTTCAGTTTACTGCCCCACTCTATGGAAATTTGCAGATGGCAACCAA : 2099
FZ99       : GAATTCATCAGCCCCATCCAATATTGACTGCACGGTGTTGCTCCGTGTGGGTAAAGATTTTCAGTTCACTGCCCCACTTTATGGCAATTTGCAGATGGCAACCAA : 2098
             GAAtTCATCAGCCCCATCCAATATTGACTGCAC GTGTTGCTCCG GTGGG AAAGATTTTCAGTT AC GCCCCACT TATGG AATtTGCAGATGGCAAC AA

                         *       2120         *       2140         *       2160         *       2180         *       2200
CH60       : CAATCAAGGTGATACCAACCAGCTAGGGGATGATGAGCCAGTTTGCTTCCTGAATTTTGAGACAGCTAATGTCCCAATACAAGGTGAATCTCACACTCTTGTTAA : 2203
A66        : CAATCAAGGTGATACCAACCAGCTAGGGGATGATGAGCCAGTTTGCTTCCTGAATTTTGAGACAGCTAATGTCCCAATACAAGGTGAATCTCACACTCTTGTTAA : 2203
C80        : CAATCAAGGTGATACCAACCAGCTAGGGGATGATGAGCCAGTTTGCTTCCTGAATTTTGAGACAGCTAATGTCCCAATACAAGGTGAATCTCACACTCTTGTTAA : 2202
MY         : CAATCAAGGTGATACCAACCAGCTAGGGGATGATGAGCCAGTTTGCTTCCTGAATTTTGAGACAGCTAATGTCCCAATACAAGGTGAATCTCACACTCTTGTTAA : 2203
FC64       : CAATCAAGGTGATACCAACCAGCTAGGGGATGATGAGCCAGTTTGCTTCCTGAATTTTGAGACAGCTAATGTCCCAATACAAGGTGAATCTCACACTCTTGTTAA : 2203
03D        : TAACCAAGGTGATTCCAACCAGTTAGGGGATGATGAGCCAGTTTGCTTTCTGAACTTTGAGACAGCTAATGTCCCGATACAAGGTGAATCTCACACTCTTGTTAA : 2200
ZJ         : TAATCAAGGCGATTCCAACCAGTTAGGGGATGATGAGCCAGTTTGCTTCCTGAACTTTGAGACAGCTAATGTCCCGATACAAGGTGAATCTCACACTCTTGTTAA : 2200
NA         : TAATCAAGGCGATTCCAACCAGTTAGGGGATGATGAGCCAGTTTGCTTCCTGAACTTTGAGACAGCTAATGTCCCGATACAAGGTGAATCTCACACTCTTGTTAA : 2203
C-XNH      : TAATCAAGGTGATTCCAACCAGTTAGGGGATGATGAGCCAGTTTGCTTCCTGAACTTTGAGACAGCTAATGTCCCGATACAAGGTGAATCTCACACTCTTGTTAA : 2202
LSD/090830 : TAACCAAGGTGACTCCAACCAGTTAGGGGATGATGAGCCAGTTTGTTTCCTGAATTTTGAGACAGCTAATGTCCCGATACAAGGTGAATCTCACACTCTTGTTAA : 2204
FZ99       : TAATCAAGGTGATTCCAACCAGTTAGGGGATGATGAGCCAGTTTGCTTCCTGAACTTTGAGACAGCTAATGTCCCGATACAAGGTGAATCTCATACTCTTGTTAA : 2203
              AAtCAAGGtGAt CCAACCAG TAGGGGATGATGAGCCAGTTTGcTTcCTGAA TTTGAGACAGCTAATGTCCC ATACAAGGTGAATCTCAcACTCTTGTTAA

                  *       2220         *       2240         *       2260         *       2280         *       2300         *
CH60       : ACACCTGTTTGGGAGGCAATGGTTAGTTAGGACTGTGCAACATGCTTCAACTGTGCAAGAGTTGGACCTCCAAGTCCCAGATAGAGGACATGCCTCTCTCATTCG : 2308
A66        : ACACCTGTCTGGGAGGCAATGGTTAGTTAGGACTGTGCAACATGCTTCAACTGTGCAAGAGTTGGACCTCCAAGTCCCAGATAGAGGACATGCCTCTCTCATTCG : 2308
C80        : ACACCTGTTTGGGAGGCAATGGTTAGTTAGGACTGTGCAACATGCTTCAACTGTGCAAGAGTTGGACCTCCAAGTCCCAGATAGAGGACATGCCTCTCTCATTCG : 2307
MY         : ACACCTGTTTGGGAGGCAATGGTTAGTTAGGACTGTGCAACATGCTTCAACTGTGCAAGAGTTGGACCTCCAAGTCCCAGATAGAGGACATGCCTCTCTCATTCG : 2308
FC64       : ACACCTGTTTGGGAGGCAATGGTTAGTTAGGACTGTGCAACATGCTTCAACTGTGCAAGAGTTGGACCTCCAAGTCCCAGATAGAGGACATGCCTCTCTCATTCG : 2308
03D        : ACACCTGTTTGGGAGGCAATGGTTGGTTAGGACTGTGCAACACGCTCCAACTGTGCAAGAGTTGGACCTCCAAGTTCCAGACAGAGGACATGCCTCTCTCATTCG : 2305
ZJ         : ACACCTGTTTGGGAGGCAATGGTTGGTTAGGACTGTGCAACACACTTCAACTGTGCAGGAGTTGGACCTCCAAGTTCCAGACAGAGGACATGCCTCTCTCATTCG : 2305
```

```
NA         : ACACCTGTTTGGGAGGCAATGGTTGGTTAGGACTGTGCAACACGCTTCAACTGTGCAGGAGTTGGACCTCCAAGTTCCAGACAGAGGACATGCCTCTCTCATTCG : 2308
C-XNH      : ACACCTGTTTGGGAGGCAATGGTTGGTTAGGACTGTGCAACACGCTTCAACTGTGCAGGAGTTGGACCTCCAAGTTCCAGACAGAGGACATGCCTCTCCCATTCG : 2307
LSD/090830 : ACACCTGTTTGGGAGGCAATGGTTGGTTAGGACTGTGCAACACGCTTTAACCGTGCAAGAGTTGGACCTCCAAGTCCCAGACAGAGGACATGCCTCTCTCATTCG : 2309
FZ99       : ACACCTGTTTGGGAGGCAATGGTTGGTTAGGACTGTGCAACACACTTCAACTGTGCAGGAGTTGGACCTCCAAGTTCCAGACAGAGGACATGCCTCTCTCATTCG : 2308
             ACACCTGTtTGGGAGGCAATGGTT GTTAGGACTGTGCAACA gCTtcAACtGTGCA GAGTTGGACCTCCAAGT CCAGA AGAGGACATGCCTCTCtCATTCG

                    2320         *        2340         *        2360         *        2380         *        2400         *
CH60       : GTTCTTTGCCTATTTTTCTGGAGAGATCATTCTTACCATTGTTAACAACGGCACTACACCAGCGATGGTGGCACATTCCTATTCTATGGATGACCTCAGTTCAGA : 2413
A66        : GTTCTTTGCCTATTTTTCTGGAGAGATCATTCTTACCATTGTTAACAACGGCACTACACCAGCGATGGTGGCACATTCCTATTCTATGGATGACCTCAGTTCAGA : 2413
C80        : GTTCTTTGCCTATTTTTCTGGAGAGATCATTCTTACCATTGTTAACAACGGCACTACACCAGCGATGGTGGCACATTCCTATTCTATGGATGACCTCAGTTCAGA : 2412
MY         : GTTCTTTGCCTATTTTTCTGGAGAGATCATTCTTACCATTGTTAACAACGGCACTACACCAGCGATGGTGGCACATTCCTATTCTATGGATGACCTCAGTTCAGA : 2413
FC64       : GTTCTTTGCCTATTTTTCTGGAGAGATCATTCTTACCATTGTTAACAACGGCACTACACCAGCGATGGTGGCACATTCCTATTCTATGGATGACCTCAGTTCAGA : 2413
03D        : GTTCTTTGCCTATTTTTCTGGAGAGATCATTCTCACTATTGTTAACAATGGCACTACACCAGCAATGGTAGCACATTCCTACTCTATGGATGATCTCAGTTCAGA : 2410
ZJ         : GTTCTTTGCCTATTTTTCTGGAGAGATCATTCTTACCATTGTTAATAATGGCACTACACCAGCAATGGTAGCACATTCTTATTCTATGGATGATCTCACTTCAGA : 2410
NA         : GTTCTTTGCCTATTTTTCTGGAGAGATCATTCTTACCATTGTTAACAATGGCACTACACCAGCAATGGTAGCACATTCTTATTCTATGGATGATCTCACTTCAGA : 2413
C-XNH      : GTTCTTTGCCTATTTTTCTGGAGAGATCATTCTTACCATTGTTAACAATGGCACTACACCAGCAATGGTAGCACATTCCTATTCTATGGATGATCTCAGTTCAGA : 2412
LSD/090830 : GTTTTTTGCCTATTTTTCTGGAGAGATCATTCTCACCATTGTTAACAATGGCACCACACCAGCAATGGTAGCACATTCCTATTCTATGGATGATCTCAGTTCAGA : 2414
FZ99       : GTTCTTTGCCTATTTTTCTGGAGAGATCATTCTTACCATTGTTAATAATGGCACTACACCAGCAATGGTAGCACATTCCTATTCTATGGATGATCTCAGTTCAGA : 2413
             GTTcTTTGCCTATTTTTCTGGAGAGATCATTCTtACcATTGTTAAcAA GGCACtACACCAGC ATGGT GCACATTCcTAtTCTATGGATGA CTCAgTTCAGA

              2420         *        2440         *        2460         *        2480         *        2500         *        2520
CH60       : GTATGCTGTTACAGCAATGGGAGGTGTGATGATCCCTGCCAATAGTGCCAAAAATATCTCTGTACCATTCTACTCTGTGACACCACTCAGGCCAACTCGACCAAT : 2518
A66        : GTATGCTGTTACAGCAATGGGAGGTGTGATGATCCCTGCCAATAGTGCCAAAAATATCTCTGTACCATTCTACTCTGTGACACCACTCAGGCCAACTCGACCAAT : 2518
C80        : GTATGCTGTTACAGCAATGGGAGGTGTGATGATCCCTGCCAATAGTGCCAAAAATATCTCTGTACCATTCTACTCTGTGACACCACTCAGGCCAACTCGACCAAT : 2517
MY         : GTATGCTGTTACAGCAATGGGAGGTGTGATGATCCCTGCCAATAGTGCCAAAAATATCTCTGTACCATTCTACTCTGTGACACCACTCAGGCCAACTCGACCAAT : 2518
FC64       : GTATGCTGTTACAGCAATGGGAGGTGTGATGATCCCTGCCAATAGTGCCAAAAATATCTCTGTACCATTCTACTCTGTGACACCACTCAGGCCAACTCGACCAAT : 2518
03D        : ATATGCTGTTACAGCAATGGGAGGTGTGATGATTCCTGCTAATAGTGCCAAAAATATATCTGTACCATTCTACTCTGTAACACCACTCAGGCCAACTCGACCAAT : 2515
ZJ         : ATATGCTGTTACAGCAATGGGAGGTGTGATGATTCCTGCTAATAGTGCCAAAAATATCTCTGTACCATTCTACTCTGTGACACCACTCAGGCCAACTCGACCAAT : 2515
NA         : ATATGCTGTTACAGCAATGGGTGGTGTGATGATTCCTGCTAATAGTGCCAAAAATATCTCTGTACCATTCTACTCTGTGACACCACTCAGGCCAACTCGACCAAT : 2518
C-XNH      : ATATGCTGTTACAGCAATGGGAGGTGTGATGATTCCTGCTAATAGTGCCAAAAATATCTCTGTACCATTCTATTCTGTGACACCACTCAGGCCAACTCGACCAAT : 2517
LSD/090830 : GTATGCTGTTACAGCAATGGGAGGTGTGATGATTCCTGCTAATAGTGCCAAAAATATTTCTGTACCATTTTACTCCGTGACACCACTCAGGCCAACTCGACCAAT : 2519
FZ99       : ATATGCTGTTACAGCAATGGGAGGTGTGATGATTCCTGCTAATAGTGCCAAAAATATCTCTGTACCATTCTACTCTGTGACACCACTCAGGCCAACTCGACCAAT : 2518
              TATGCTGTTACAGCAATGGGaGGTGTGATGAT CCTGC AATAGTGCCAAAAATATcTCTGTACCATTcTAcTCtGTgACACCACTCAGGCCAACTCGACCAAT

                        *        2540         *        2560         *        2580         *        2600         *        2620
CH60       : TCCTGGCACATCAGAGGCAACTTTTGGCAGGCTGTTCATGTGGACTCAATCAGGAAGTCTTTCAGTTTTTATGGGTCTCAAAAAGCCAGCTCTCTTCTTTTCCACT : 2623
A66        : TCCTGGCACATCAGAGGCAACTTTTGGCAGGCTGTTCATGTGGACTCAATCAGGAAGTCTTTCAGTTTTTATGGGTCTCAAAAAGCCAGCTCTCTTCTTTTCCACT : 2623
C80        : TCCTGGCACATCAGAGGCAACTTTTGGCAGGCTGTTCATGTGGACTCAATCAGGAAGTCTTTCAGTTTTTATGGGTCTCAAAAAGCCAGCTCTCTTCTTTTCCACT : 2622
MY         : TCCTGGCACATCAGAGGCAACTTTTGGCAGGCTGTTCATGTGGACTCAATCAGGAAGTCTTTCAGTTTTTATGGGTCTCAAAAAGCCAGCTCTCTTCTTTTCCACT : 2623
FC64       : TCCTGGCACATCAGAGGCAACTTTTGGCAGGCTGTTCATGTGGACCCAATCAGGAAGTCTTTCAGTTTTTATGGGTCTCAAAAAGCCAGCTCTCTTCTTTTCCACT : 2623
03D        : TCCTGGCACATCAGAAGCAACCTTTGGCAGGCTGTTCATGTGGACTCAATCAGGAAGTCTTTCAGTTTTTATGGGTCTCAAGAAACCAGCTCTCTTCTTCCCACT : 2620
ZJ         : TCCTGGCACATCAGAAGCAACCTTTGGCAGGCTGTTCATGTGGACTCAATCAGGAAGTCTTTCAGTTTTTATGGGTCTTAAGAAGCCAGCTCTCTTCTTCCCGCT : 2620
NA         : TCCTGGCACATCAGAAGCAACCTTTGGCAGGCTGTTCATGTGGACTCAATCAGGAAGTCTTTCAGTTTTTATGGGTCTTAAGAAGCCAGCTCTCTTCTTCCCGCT : 2623
C-XNH      : ACCTGGCACATCAGAAGCAACCTTTGGAAGGCTGTTCATGTGGACTCAATCAGGAAGTCTTTCAGTTTTTATGGGTCTCAAGAAGCCAGCCCTCTTCTTCCCGCT : 2622
LSD/090830 : TCCTGGCACATCAGAAGCAACCTTTGGCAGGCTGTTCATGTGGACTCAATCAGGAAGTCTTTCAGTTTTTATGGGTCTCAAGAAGCCAGCTCTCTTCTTCCCACT : 2624
FZ99       : TCCTGGCACATCAGAAGCAACCTTTGGCAGGCTGTTCATGTGGACTCAATCAGGAAGTCTTTCAGTTTTTATGGGTCTCAAGAAGCCAGCTCTCTTCTTCCCGCT : 2623
             tCCTGGCACATCAGA GCAAC TTTGGcAGGCTGTTCATGTGGACtCAATCAGGAAGTCTTTCAGTTTTTATGGGTCTcAA AAgCCAGCtCTCTTCTT CC CT

                  *        2640         *        2660         *        2680         *        2700         *        2720         *
CH60       : CCCTGCTCCCACTTCCACAACACTATCACGGGGATCCAATAGTGTTATTCCCACATTGGATCAGTCTGGAGATGAAGTAGATTGTCACTTCTGCAAAATTTGTTC : 2728
A66        : CCCTGCTCCCACTTCCACAACACTATCACGGGGATCCAATGGTGTTATTCCCACATTGGATCAGTCTGGAGATGAAGTAGATTGTCACTTCTGCAAAATTTGTTC : 2728
C80        : CCCTGCTCCCACTTCCACAACACTATCACGGGGATCCAATGGTGTTATTCCCACATTGGATCAGTCTGGAGATGAAGTAGATTGTCACTTCTGCAAAATTTGTTC : 2727
MY         : CCCTGCTCCCACTTCCACAACACTATCACGGGGATCCAATAGTGTTATTCCCACATTGGATCAGTCTGGAGATGAAGTAGATTGTCACTTCTGCAAAATTTGTTC : 2728
FC64       : CCCTGCTCCCACTTCCACAACACTATCACGGGGATCCAATGGTTTTATTCCCACATTGGATCAGTCTGGAGATGAAGTAGATTGTCACTTCTGCAAAATTTGTTC : 2728
03D        : CCCTGCTCCCACTTCCACAACAACATCACGGGGGTCCAATGGTGTTATTCCCACATTGGATCAGTCTGGGGATGAAGTAGATTGCCACTTCTGCGAAATTTGTTC : 2725
ZJ         : CCCTGCTCCCACTTCCACAACACTATCACGGAAATCCAATGATGTTATTCCCACATCGGATCAGTCTGGGGATGAAGTAGACTGTCACTTCTGCGAAATTTGTTC : 2725
NA         : CCCTGCTCCCACTTCCACAACACTAGCACGGAAATCCAATGATGTTATTCCCACATCAGATCAGTCTGGGGATGAAGTAGATTGTCACTTCTGCGAAATCTGTTC : 2728
C-XNH      : CCCTGCTCCCACTTCCACAACACTATCACGGAAATCCAATGATGTTATTCCCACATTGGATCAGTCTGGGGATGAAGTAGATTGTCACTTCTGCAAAATCTGTTC : 2727
LSD/090830 : CCCTGCTCCCACTTCTACAACATTATCACGGAAATCCAATGATGTTATTCCCACATTGGATCAGTCTGGGGATGAAGTAGATTGCCACTTCTGCGAAATTTGTTC : 2729
FZ99       : CCCTGCTCCCACTTCCACAACACTATCACGGAAATCCAATGATGTTATTCCCACATTGGATCAGTCTGGGGATGAAGTAGATTGTCACTTCTGCGAAATCTGTTC : 2728
             CCCTGCTCCCACTTCcACAACACtAtCACGG  aTCCAATg TgTTATTCCCACATtgGATCAGTCTGG GATGAAGTAGAtTGtCACTTCTGC AAAT TGTTC

                    2740         *        2760         *        2780         *        2800         *        2820         *
CH60       : TAAAATGAAGAGGATGTGGAAGCCAAAAGGGCACTTCAGATTTTGCCTTAGACTCAAAACACTAGCATTTGAACTCGATCTGGAAATTGAATCTGACCAAATTAG : 2833
A66        : TAAAATGAAGAGGATGTGGAAGCCAAAAGGGCACTTCAGATTTTGCCTTAGACTCAAAACACTAGCATTTGAACTCGATCTGGAAATTGAATCTGACCAAATTAG : 2833
C80        : TAAAATGAAGAGGATGTGGAAGCCAAAAGGGCACTTCAGATTTTGCCTTAGACTCAAAACACTAGCATTTGAACTCGATCTGGAAATTGAATCTGACCAAATTAG : 2832
MY         : TAAAATGAAGAGGATGTGGAAGCCAAAAGGGCACTTCAGATTTTGCCTTAGACTCAAAACACTAGCATTTGAACTCGATCTGGAAATTGAATCTGACCAAATTAG : 2833
FC64       : TAAAATGAAGAGGATGTGGAAGCCAAAAGGGTACTTCAGATTTTGCCTTAGACTCAAAACACTAGCATTTGAACTCGATCTGGAAATTGAATCTGACCAAATTAG : 2833
03D        : TAAAATGAAGAGGATGTGGAAGCCAAGAGGGCACTTCAGATTCTGTCTTAGACTCAAAACACTAGCATTTGAACTCAATCTGGAAATTGAATCTGACCAAATTAG : 2830
ZJ         : TAAAATGAAGAGGATGTGGAAGCCAAGAGGGCACTTTAGATTTTGCCTTAGACTCAAAACACTAGCATTTGAACTCAACCTGGAAATTGAATCTGACCAAATTAG : 2830
NA         : CAAAATGAAGAGGATGTGGAAGCCAAGAGGGCACTTTAGATTTTGCCTTAGACTCAAAACACTAGCATTTGAACTCAACCTGGAAATTGAATCTGACCAAATTAG : 2833
C-XNH      : TAAAATGAAGAGGATGTGGAAGCCAAGAGGGCACTTCAGATTTTGCCTTAGACTCAAAACACTAGCATTTGAACTCAACCTGGAAATTGAATCTGACCAAATTAG : 2832
LSD/090830 : TAAAATGAGGAGGATGTGGAAGCCAAGAGGGCGCTTCAGATTTTGCCTTAGACTCAAAACACTAGCATTTGAACTCAATTTGGAACTTGAATCTGACCAAATTAG : 2834
FZ99       : TAAAATGAAGAGGATGTGGAAGCCAAGAGGGCACTTCAGATTTTGCCTTAGACTCAAAACACTAGCATTTGAACTCAACCTGGAAATTGAATCTGACCAAATTAG : 2833
             tAAAATGAaGAGGATGTGGAAGCCAA AGGGcaCTTcAGATTtTGcCTTAGACTCAAAACACTAGCATTTGAACTC A cTGGAAaTTGAATCTGACCAAATTAG

              2840         *        2860         *        2880         *        2900         *        2920         *        2940
CH60       : AAACAAGAAAGATCTCACTACTGAAGGAGTGGAACCGAACCCAGGACCTATCTTAGTTGTTGGCAAATCAGGAAGTGGCAAGAGCCGACTTTGCAACGTTCTAGC : 2938
A66        : AAACAAGAAAGATCTCACTACTGAAGGAGTGGAACCGAACCCAGGACCTATCTTAGTTGTTGGCAAATCAGGAAGTGGCAAGAGCCGACTTTGCAACGTTCTAGC : 2938
C80        : AAACAAGAAAGATCTCACTACTGAAGGAGTGGAACCGAACCCAGGACCTATCTTAGTTGTTGGCAAATCAGGAAGTGGCAAGAGCCGACTTTGCAACGTTCTAGC : 2937
MY         : AAACAAGAAAGATCTCACTACTGAAGGAGTGGAACCGAACCCAGGACCTATCTTAGTTGTTGGCAAATCAGGAAGTGGCAAGAGCCGACTTTGCAACGTTCTAGC : 2938
FC64       : AAACAAGAAAGATCTCACTACTGAAGGAGTGGAACCGAACCCAGGACCTATCTTAGTTGTTGGCAAATCAGGAAGTGGCAAGAGCCGACTTTGCAACGTTCTAGC : 2938
03D        : AAACAAGAAAGATCTCACTACTGAAGGAGTGGAACCAAACCCAGGACCTATCTTAGTTGTTGGCAAATCAGGAAGTGGCAAGAGCCGACTTTGTAATGTTCTAGC : 2935
ZJ         : AAACAAGAAAGATCTTACTACTGAAGGAGTGGAACCAAACCCAGGACCTATCTTAGTTGTTGGCAAATCAGGAAGTGGCAAGAGCCGACTTTGTAATGTTCTAGC : 2935
NA         : AAACAAGAAAGATCTTACTACTGAAGGAGTGGAACCAAACCCAGGACCTATCTTAGTTGTTGGCAAATCAGGAAGTGGTAAGAGCCGACTTTGTAATGTTCTAGC : 2938
C-XNH      : AAATAAGAAAGATCTTACTACTGAAGGAGTGGAACCAAACCCAGGACCTATCTTAGTTGTTGGCAAGTCAGGAAGTGGCAAGAGTCGACTTTGCAATGTTCTAGC : 2937
LSD/090830 : AAATAAGAAAGATCTCACTACTGAAGGAGTGGAACCAAATCCAGGACCTATCTTAGTTGTTGGCAAATCAGGAAGTGGCAAGAGCCGACTTTGTAATGTTCTAGC : 2939
FZ99       : AAACAAGAAAGATCTTACTACTGAAGGAGTGGAACCAAACCCAGGACCTATCTTAGTTGTTGGCAAATCAGGAAGTGGCAAGAGCCGACTTTGTAATGTTCTAGC : 2938
             AAAcAAGAAAGATCT ACTACTGAAGGAGTGGAACC AAcCCAGGACCTATCTTAGTTGTTGGCAAaTCAGGAAGTGGcAAGAGcCGACTTTG AA GTTCTAGC

                        *        2960         *        2980         *        3000         *        3020         *        3040
CH60       : TGGTGTGGAGCTTTTTGAATCAAAACTGTCACCACGTTCAGTTACAATGGTTCATCAGGTTGAGACAATTGACATTGAAGGGAAGAAGGTGACAATAGTGGATTC : 3043
A66        : TGGTGTGGAGCTTTTTGAATCAAAACTGTCACCACGTTCAGTTACAATGGTTCATCAGGTTGAGACAATTGACATTGAAGGGAAGAAGGTGACAATAGTGGATTC : 3043
C80        : TGGTGTGGAGCTTTTTGAATCAAAACTGTCACCACGTTCAGTTACAATGGTTCATCAGGTTGAGACAATTGACATTGAAGGGAAGAAGGTGACAATAGTGGATTC : 3042
MY         : TGGTGTGGAGCTTTTTGAATCAAAACTGTCACCACGTTCAGTTACAATGGTTCATCAGGTTGAGACAATTGACATTGAAGGGAAGAAGGTGACAATAGTGGATTC : 3043
FC64       : TGGTGTGGAGCTTTTGGATCAAAACTGTCACCACGTTCAGTTACAATGGTTCATCAGGTTGAGACAATTGACATTGAAGGGAAGAAGGTGACAATAGTGGATTC : 3043
03D        : TGGTGTGGAGCTCTTTGAATCAAAACTGTCACCACGTTCAGTTACAATGGTTCATCAGGTTGAGACAATTGACGTTGAGGGGAAGAAGGTGACGATAGTGGATTC : 3040
ZJ         : TGGTGTGGAGCTCTTTGAATCAAAACTGTCACCACGTTCAGTTACAATGGTTCATCAGGTTGAGACAATTGATGTTGAAGGGAAGAAGGTGACAATAGTGGATTC : 3040
NA         : TGGTGTGGAGCTCTTTGAATCAAAACTGTCACCACGTTCAGTTACAATGATTCATCAGGTTGAGACAATTGATGTTGAAGGGAAGAAGGTGACAATAGTGGATTC : 3043
C-XNH      : TGGTGTGGAGCTCTTTGAATCAAAACTATCACCACGTTCAGTTACAATGGTTCATCAGGTTGAGACAATTGATGTTGAGGGGAAGAAGGTGACGATAGTGGATTC : 3042
LSD/090830 : TGGTGTGGAGCTCTTTGAATCAAAATTGTCACCATGTTCAGTCACAATGGTTCATCAGGTTGAAACGATTGACGTTGAGGGGAAGAAGGTGACGATAGTGGATTC : 3044
```

```
FZ99       : TGGTGTGGAGCTCTTTGAATCAAAACTGTCACCACGTTCAGTTACAATGGTTCATCAGGTTGAGACAATTGACATTGAAGGGAAGAAGGTGACGATAGTGGATTC : 3043
             TGGTGTGGAGCT TTTGaATCAAAAcTgTCACCAcGTTCAGTtACAATGgTTCATCAGGTTGAgACaATTGA  TTGA GGGAAGAAGGTGAC ATAGTGGATTC

                  *     3060        *     3080        *     3100        *     3120        *     3140        *
CH60       : ACCCGAAACACCAAATTATGATGGCCCCATTTCTGCCTTTTTTTACTTGATTGAGGCAGGCAGGTTCACAGCTGAAGACAAAGAGTATATCCAAATGATGAGAAA : 3148
A66        : ACCCGAAACACCAAATTATGATGGCCCCATTTCTGCCTTTTTTTACTTGATTGAGGCAGGCAGGTTCACAGCTGAAGACAAAGAGTACATCCAAATGATGAGAAA : 3148
C80        : ACCCGAAACACCAAATTATGATGGCCCCATTTCTGCCTTTTTTTACTTGATTGAGGCAGGCAGGTTCACAGCTGAAGACAAAGAGTACATCCAAATGATGAGAAA : 3147
MY         : ACCCGAAACACCAAATTATGATGGCCCCATTTCTGCCTTTTTTTATTTGATTGAGGCAGGCAGGTTCACAGCTGAAGACAAAGAGTATATCCAAATGATGAGAAA : 3148
FC64       : ACCCGAAACACCAAATTATGATGGCCCCATTTCAGCCTTTTTTTACTTGATTGAGGCAGGCAGGTTCACAGCTGAAGACAAAGAGTACATCCAAATGATGAGAAA : 3148
03D        : ACCTGAAACACCAAATTATGATGGCCCCATTTCTGCCTTTTTTTACTTGATTGAGGCAGGCAGGTTCACAGCTGAAGACAAGGAGTACATCCAAATGATGAGAAA : 3145
ZJ         : ACCTGAAACACCTAACTATGATGGCCCCATTTCTGCTTTTTTTTACTTGATTGAGGCAGGCAGGTTCACAGCTGAAGACAAGGAGTACATCCAAATGATGAGAAA : 3145
NA         : ACCTGAAACACCTAACTATGATGGCCCCATTTCTGCTTTTTTTTACTTGATTGAGGCAGGCAGGTTCACAGCTGAAGACAAGGAGTACATCCAAATGATGAGAAA : 3148
C-XNH      : GCCTGAAACACCAAACTATGATGGCCCCATTTCTGCTTTTTTTTACTTGATTGAGGCAGGCAGGTTCACAGTTGAAGACAAGGAGTACGTCCAAATGATGAGAAA : 3147
LSD/090830 : ACCTGAAACACCAAATTATGATGGCCCCATTTCTGCTTTTTTTTACTTGATTGAGGCAGGCAGGTTCACAGCTGAAGACAAGGAGTACATCCAAATGATGAGAAA : 3149
FZ99       : ACCTGAAACACCAAACTATGATGGCCCCATTTCTGCCTTTTTTTACTTGATTGAGGCAGGCAGGTTCACAGCTGAAGACAAGGAGTACATCCAAATGATGAGAAA : 3148
             aCC GAAACACCaAA TATGATGGCCCCATTTCtGC TTTTTTTAcTTGATTGAGGCAGGCAGGTTCACAGcTGAAGACAA GAGTAcaTCCAAATGATGAGAAA

                  3160        *     3180        *     3200        *     3220        *     3240        *
CH60       : GCGCTTTCCAAGCTTTGAGAAATCCACTATTTTGATTTTGAACCGAGCAGATGAATTGAAAACTGATGATGATATAAGAAATTGGATTAAGACATCTGGAGAGTT : 3253
A66        : GCGCTTTCCAAGCTTTGAGAAATCCACTATTTTGATTTTGAACCGAGCAGATGAATTGAAAACTGATGATGATATAAGAAATTGGATTAAGACATCTGGAGAGTT : 3253
C80        : GCGCTTTCCAAGCTTTGAGAAATCCACTATTTTGATTTTGAACCGAGCAGATGAATTGAAAACTGATGATGATATAAGAAATTGGATTAAGACATCTGGAGAGTT : 3252
MY         : GCGCTTTCCAAGCTTTGAGAAATCCACTATTTTGATTTTGAACCGAGCAGATGAATTGAAAACTGATGATGATATAAGAAATTGGATTAAGACATCTGGAGAGTT : 3253
FC64       : GCGCTTTCCAAGCTTTGAGAAATCCACTATTTTGATTTTGAACCGAGCAGATGAATTGAAAACTGATGATGATATAAGAAATTGGATTAAGACATCTGGAGAGTT : 3253
03D        : ACGCTTTCCAGGCTTTGAGAAATCCACTATTTTGATTTTGAACCGAGCAGATGAATTGAAAACTGATGATGATATAAGAAATTGGGTTAAGACATCTGGAGAGTT : 3250
ZJ         : GCGCTTTCCAGGCTTTGAGAAATCCACTATTTTGATTTTGAACCGAGCAGATGAATTGAAAACTGATGATGATATAAGAAAGTGGATTAAGACATCTGGAGAGTT : 3250
NA         : GCGCTTTCCAGGCTTTGAGAAGTCCACTATTTTGATTTTGAACCGAGCAGATGAATTGAAAACTGATGATGATATAAGAAAGTGGATTAAGACATCTGGAGAGTT : 3253
C-XNH      : GCGCTTTCCAGGCTTTGAGAAATCCACTATTTTGATTTTGAACCGAGCAAGTGAATTGAAAACTGATGATGATATAAGAAAGTGGGTTAAGACATCTGGAGAGTT : 3252
LSD/090830 : GCGCTTTCCTGGCTTTGAGAAATCCACTATTCTGATTTTGAACCGAGCAGATGAATTGAAAACCGATAATGATATAAGAAATTGGGTTAAGACATCTGGAGAGTT : 3254
FZ99       : GCGCTTTCCAGGCTTTGAGAAATCCACTATTTTGATTTTGAACCGAGCAGATGAATTGAAAACTGATGATGATATAAGAAAGTGGGTTAAGACATCTGGAGAATT : 3253
             gCGCTTTCCa GCTTTGAGAAaTCCACTATTtTGATTTTGAACCGAGCAgaTGAATTGAAAACtGATgATGATATAAGAAA TGG TTAAGACATCTGGAGAgTT

              3260        *     3280        *     3300        *     3320        *     3340        *     3360
CH60       : GGACTCTTTAGTGAGAGCCTGTGGGGGGCGTATAGCTAAATTTCACCGGTATAAAATAAACAGGGCTAAGTTGTTAGAGAAAGCAGCTACCCTTCCTGAGTTTGT : 3358
A66        : GGACTCTTTAGTGAGAGCCTGTGGGGGGCGTATAGCTAAATTTCACCGGTATAAAATAAACAGGGCTAAGTTGTTAGAGAAAGCAGCTACCCTTCCTGAGTTTGT : 3358
C80        : GGACTCTTTAGTGAGAGCCTGTGGGGGGCGTATAGCTAAATTTCACCGGTATAAAATAAACAGGGCTAAGTTGTTAGAGAAAGCAGCTACCCTTCCTGAGTTTGT : 3357
MY         : GGACTCTTTAGTGAGAGCCTGTGGGGGGCGTATAGCTAAATTTCACCGGTATAAAATAAACAGGGCTAAGTTGTTAGAGAAAGCAGCTACCCTTCCTGAGTTTGT : 3358
FC64       : GGACTCTTTAGTGAGAGCCTGTGGGGGGCGTATAGCTAAATTTCACCGGTATAAAATAAACAGGGCTAAGTTGTTAGAGAAAGCAGCTACCCTTCCTGAGTTTGT : 3358
03D        : GGACTCTCTAGTGAGAGCCTGTGGGGGGCGTATAGCTAAATTTCACCGGCACAAAATAAACAGGGCTAAGTTATTAGAGAAAGCAGCCACCCTTCCTGAGTTTGT : 3355
ZJ         : GGACTCTCTAGTGAGAGCTTGTGGGGGGCGTATAGCTAAACTCTACCGGCACAAAATAAATAGGACTAAGTTGTTAGAGAAAGCAGCCACCCTTCCTGAGTTTGT : 3355
NA         : GGACTCTCTAGTGAGAGCTTGTGGGGGGCGTATAGCTAAATTTCACCGGCACAAAATAAATAGGACTAAGTTGTTAGAGAAAGCAGCCACCCTTCCTGAGTTTGT : 3358
C-XNH      : GGACTCTCTAGTGAGAGCTTGTGGGGGGCGTATAGCTAAATTTTACTGGCACAAAATAAACAGGACTAAGTTGTTGGAGAGAAAGCAGCCACCCTTCCTGAGTTTGT : 3357
LSD/090830 : GGACTCTCTAGTGAGAGCCTGTGGGGGGCGTATAGCTAAATTTCACCGGCATAAAATAAACAGGGCTAAGTTGCTAGAGAAAGCAGCCACTCTTCCTGAGTTTGT : 3359
FZ99       : GGACTCTCTAGTGAGAGCTTGTGGGGGGCGTATAGCTAAATTTTACCGGCACAAAATAAACAGGGCTAAGTTGTTAGAGAAAGCAGCCACCCTTCCTGAGTTTGT : 3358
             GGACTCT TAGTGAGAGC TGTGGGGGGCGTATAGCTAAAtTt ACcGG A AAAATAAAcAGG CTAAGTTgtTaGAGAAAGCAGC ACcCTTCCTGAGTTTGT

                  *     3380        *     3400        *     3420        *     3440        *     3460
CH60       : CTCTCATTTGCCAAGACTTGTCTACAAGGATAGAAAAATGTACCGCCATTATGGTGTGCAGTGTGGTTCCACAGTTTTCCATATGGACTCAGAGAACATCCTTGA : 3463
A66        : CTCTCATTTGCCAAGACTTGTCTACAAGGATAGAAAAATGTACCGCCATTATGGTGTGCAGTGTGGTTCCACAGTTTTCCATATGGACTCAGAGAACATCCTTGA : 3463
C80        : CTCTCATTTGCCAAGACTTGTCTACAAGGATAGAAAAATGTACCGCCATTATGGTGTGCAGTGTGGTTCCACAGTTTTCCATATGGACTCAGAGAACATCCTTGA : 3462
MY         : CTCTCATTTGCCAAGACTTGTCTACAAGGATAGAAAAATGTACCGCCATTATGGTGTGCAGTGTGGTTCCACAGTTTTCCATATGGACTCAGAGAACATCCTTGA : 3463
FC64       : CTCTCATTTGCCAAGACTTGTCTACAAGGATAGAAAAATGTACCGCCATTATGGTGTGCAGTGTGGTTCCACAGTTTTCCATATGGACTCAGAGAACATCCTTGA : 3463
03D        : CTCTCATTTGCCAAGACTTGTCTACAAGGATAGAAAAATGTACCGCCATTATGGTGTGCAGTGTGGTTCCACAGTCTTCCACATGGACTCAGAGAACATCCTTGA : 3460
ZJ         : CTCTCATTTGCCAAGACTTGTCTATAAGGATAGAAGAATGTACCGCCATTATGGTGTGCAGTGTGGTTCCACAGTCTTCCACATGGACTCAGAGAACATCCTTGA : 3460
NA         : CTCTCATTTGCCAAGACTTGTCTATAAGGATAGAAGAATGTACCGCCATTATGGTGTGCAGTGTGGTTCCACAGTCTTCCACATGGATTCAGAGAACATCCTTGA : 3463
C-XNH      : CTCTCATTTGCCAAGACTTGTTTATAAGGATAGAAGAATCTACCGCCATTATGGTGTGCAGTGTGGTTCCACAGTCTTCCACATGGACTCAGAGAACATCCTTGA : 3462
LSD/090830 : CTCCCATTTGCCAAGACTTGTCTACAAGGATAGAAAAATGTACCGCCATTATGGTGTGCAGTGTGGTTCCACGGTCTTCCACATGGACTCAGAGAACATCCTTGA : 3464
FZ99       : CTCTCATTTGCCAAGACTTGTCTATAAGGATAGAAGAATGTACCGCCATTATGGTGTGCAGTGTGGTTCCACAGTCTTCCACATGGACTCAGAGAACATCCTTGA : 3463
             CTCtCATTTGCCAAGACTTGTcTA AAGGATAGAA AATgTACCGCCATTATGGTGTGCAGTGTGGTTCCACaGT TTCCA ATGGAcTCAGAGAACATCCTTGA

                  *     3480        *     3500        *     3520        *     3540        *     3560        *
CH60       : GTCAGCATTAAATGGTGAAGTCACAATCAAGCAAGAGAAGTGGAATGGCAATTGGAAACCAGCAGGTGATCACATGCAGTCAACAGCTAGCATGTATCTTAAATC : 3568
A66        : GTCAGCATTAAATGGTGAAGTCACAATCAAGCAAGAGAAGTGGAATGGCAATTGGAAACCAGCAGGTGATCACATGCAGTCAACAGCTAGCATGTATCTTAAATC : 3568
C80        : GTCAGCATTAAATGGTGAAGTCACAATCAAGCAAGAGAAGTGGAATGGCAATTGGAAACCAGCAGGTGATCACATGCAGTCAACAGCTAGCATGTATCTTAAATC : 3567
MY         : GTCAGCATTAAATGGTGAAGTCACAATCAAGCAAGAGAAGTGGAATGGCAATTGGAAACCAGCAGGTGATCACATGCAGTCAACAGCTAGCATGTATCTTAAATC : 3568
FC64       : GTCAGCATTAAATGGTGAAGTCACAATCAAGCAAGAGAAGTGGAATGGCAATTGGAAACCAGCAGGTGATCACATGCAGTCAACAGCTAGCATGTATCTTAAATC : 3568
03D        : GTCAGCATTAAATGGTGAAGTCACAATTAAGCAGGAGAAGTGGAATGGCAATTGGAAACCAGCAGGTGATCACATGCAGTCAACAGCCAGCATGTATCTTAAATC : 3565
ZJ         : GTCAGCATTAAATGGTGAAGTCACAATTAAGCAGGAGAAGTGGAATGGTAATTGGAAACCAGCAGGTGATCACATGCAGTCAACAGCCAGCATGTATCTTAAATC : 3565
NA         : GTCAGCATTAAATGGTGAAGTCACAATTAAGCAGGAGAAGTGGAATGGTAATTGGAAACCAGCAGGTGATCACATGCAGTCAACAGCCAGCATGTATCTTAAATC : 3568
C-XNH      : GTCAGCATTAAATGGTGAAGTCACAATTAAGCAGGAGAAGTGGAATGGTAATTGGAAACCAGCAGGTGATCACATGCAGTCAACAGCCAGTATGTATCTTAAATC : 3567
LSD/090830 : GTCAGCATTAAATGGTGAAGTTACAATCAAGCAGGAGAAGTGGAATGGCAATTGGAAACCAGCAGGTGATCACATGCAGTCAACAGCCAGCATGTATCTTAAATC : 3569
FZ99       : GTCAGCATTAAATGGTGAAGTCACAATTAAGCAGGAGAAGTGGAATGGTAATTGGAAACCAGCAGGTGATCACATGCAGTCAACAGCCAGCATGTATCTTAAATC : 3568
             GTCAGCATTAAATGGTGAAGTcACAAT AAGCA GAGAAGTGGAATGG AATTGGAAACCAGCAGGTGATCACATGCAGTCAACAGC AGcATGTATCTTAAATC

                  3580        *     3600        *     3620        *     3640        *     3660        *
CH60       : ACAGACTATGCCAAAATTCACTTTTTCAATTGATGAAAACTGTGAAACCTGGGCTAGACACTTGTTGGGCGACTATGGGGAAACACAAGGTCAGATTTTTAAGGA : 3673
A66        : ACAGACTATGCCAAAATTCACTTTTTCAATTGATGAAAACTGTGAAACCTGGGCTAGACACTTGTTGGGCGACTATGGGGAAACACAAGGTCAGATTTTTAAGGA : 3673
C80        : ACAGACTATGCCAAAATTCACTTTTTCAATTGATGAAAACTGTGAAACCTGGGCTAGACACTTGTTGGGCGACTATGGGGAAACACAAGGTCAGATTTTTAAGGA : 3672
MY         : ACAGACTATGCCAAAATTCACTTTTTCAATTGATGAAAACTGTGAAACCTGGGCTAGACACTTGTTGGGCGACTATGGGGAAACACAAGGTCAGATTTTTAAAGA : 3673
FC64       : ACAGACTATGCCAAAATTCACTTTTTCAATTGATGAAAACTGTGAAACCTGGGCTAGACACTTGTTGGGCGACTATGGGGAAACACAAGGTCAGATTTTTAAGGA : 3673
03D        : ACAGACTATGCCAAAATTTACTTTTTCAATTGATGAAAATTGTGAAACCTGGGCTAGACATTTGTTGGGCGATTATGGGGAAACACAAGGTCAGATTTTTAAGGA : 3670
ZJ         : ACAGACTATGCCAAAATTTACTTTTTCAATTGATGAAAATTGTGAAACCTGGGCTAGACATTTGTTGGGCGATTATGGGGAAACACAAGGTCAGATTTTTAAGGA : 3670
NA         : ACAGACTATGCCAAAATTTACCTTCTCAATTGATGAAAATTGTGAAACCTGGGCTAGACATTTGTTAGGCGATTATGGGGAAACACAAGGTCAGATTTTTAAGGA : 3673
C-XNH      : ACAGACTATGCCAAAATTTACTTTTTCAATTGATGAAAATTGTGAAACCTGGGCTAGACATTTGTTGGGCGACTATGGGGAAACACAAGGTCAGATTTTTAAGGA : 3672
LSD/090830 : ACAGACTATGCCAAAATTTGCTTTTTCAATTGATGAAAATTGTGAAACCTGGGCTAGACATTTGTTGGGCGACTATGGGGAAACACAAGGTCAGATTCTTAAGGA : 3674
FZ99       : ACAGACTATGCCAAAATTTACTTTTTCAATTGATGAAAATTGTGAAACCTGGGCTAGACATTTGTTGGGCGACTATGGGGAAACACAAGGTCAGATTTTTAAGGA : 3673
             ACAGACTATGCCAAAATT aCtTTtTCAATTGATGAAAA TGTGAAACCTGGGCTAGACA TTGTTgGGCGA TATGGGGAAACACAAGGTCAGATTtTTAAgGA

              3680        *     3700        *     3720        *     3740        *     3760        *     3780
CH60       : AAGGCTTATGTGGGCTGTAGCCCTTGGTTTTTTTATGACAATGAAAATCACTACAGACCAATCATTTCCAGGTAAAGATGCAATACACACAGTTCTCACCAAAAT : 3778
A66        : AAGGCTTATGTGGGCTGTAGCCCTTGGTTTTTTTATGACAATGAAAATCACTACAGACCAATCATTTCCAGGTAAAGATGCAATACACACAGTTCTCACCAAAAT : 3778
C80        : AAGGCTTATGTGGGCTGTAGCCCTTGGTTTTTTTATGACAATGAAAATCACTACAGACCAATCATTTCCAGGTAAAGATGCAATACACACAGTTCTCACCAAAAT : 3777
MY         : AAGGCTTATGTGGGCTGTAGCCCTTGGTTTTTTTATGACAATGAAAATCACTACAGACCAATCATTTCCAGGTAAAGATGCAATACACACAGTTCTCACCAAAAT : 3778
FC64       : AAGGCTTATGTGGGCTGTAGCCCTTGGTTTTTTTATGACAATGAAAATCACTACAGACCAATCATTTCCAGGTAAAGATGCAATACACACAGTTCTCACCAAAAT : 3778
03D        : GAGGCTTATGTGGGCTGCAGCCCTTGGTTTTTTTATGACAATGAAAATCACTACAGACCAATCATTTCCAGGTAAAGATGCAATACACACAGTCCTCACCAAAAT : 3775
ZJ         : AAGGCTTATGTGGGCTGCAGCCCTTGGTTTTTTTATGACAATGAAAATCACTACAGACCAATCATTTCCAGGCAAAGATGCAATACACACAGTTCTCACCAAAAT : 3775
NA         : AAGGCTTATGTGGGCTGCAGCCCTTGGTTTTTTTATGACAATGAAAATCACTACAGACCAATCATTTCCAGGCAAAGATGCAATACACACAGTTCTCACCAAAAT : 3778
C-XNH      : AAGGCTTATGTGGGCTGCAGCCCTTGGTTTTTTTATGACAATGAAAATCACTACAGACCAATCATTTCCAGGTAAAGATGCAATACACACAGTTCTCACCAAAAT : 3777
LSD/090830 : GAGGCTTATGTGGGCTGCAGCTCTTGGTTTTTTTATGACAATGAAAATCACCACAGACCAATCATTTCCAGGTAAAGATGCAATACACACAGTTCTCACCAAAAT : 3779
FZ99       : AAGGCTTATGTGGGCTGTAGCCCTTGGTTTTTTTATGACAATGAAAATCACTACAGACCAATCATTTCCAGGTAAAGATGCAATACACACAGTTCTCACCAAAAT : 3778
             aAGGCTTATGTGGGCTG AGCcCTTGGTTTTTTTATGACAATGAAAATCACtACAGACCAATCATTTCCAGGtAAAGATGCAATACACACAGTtCTCACCAAAAT
```

```
                   *      3800         *      3820         *      3840         *      3860         *      3880
CH60       : TTCAAATTTTATTTTTGGGGGCTTGGAAAATGAAGTTGTCCGTGTTGTCATCAGGACTGTGATTCGCATTGTTTGTTATCTCATTTTGTATATACATTCTCCCAA : 3883
A66        : TTCAAATTTTATTTTTGGGGGCTTGGAAAATGAAGTTGTCCGTGTTGTCATCAGGACTGTGATTCGCATTGTTTGTTATCTCATTTTGTATATACATTCTCCCAA : 3883
C80        : TTCAAATTTTATTTTTGGGGGCTTGGAAAATGAAGTTGTCCGTGTTGTCATCAGGACTGTGATTCGCATTGTTTGTTATCTCATTTTGTATATACATTCTCCCAA : 3882
MY         : TTCAAATTTTATTTTTGGGGGCTTGGAAAATGAAGTTGTCCGTGTTGTCATCAGGACTGTGATTCGCATTGTTTGTTATCTCATTTTGTATATACATTCTCCCAA : 3883
FC64       : TTCAAATTTTATTTTTGGGGGCTTGGAAAATGAAGTTGTCCGTGTTGTCATCAGGACTGTGATTCGCATTGTTTGTTATCTCATTTTGTATATACATTCTCCCAA : 3883
03D        : TTCAAATTTTATTTTTGGGGGCTTGGAAAATGAAGTTGTCCGTGTTGTCATCAGGACTGTGATTCGCATTGTTTGTTACCTTATTTTGTATATACATTCTCCCAA : 3880
ZJ         : TTCAAATTTTATTTTTGGGGGCTTGGAAAATGAAGTTGTCCGTGTTGTTATTAGGACTGTGATTCGCATTGTTTGTTATCTTATTTTGTATATACATTCTCCCAA : 3880
NA         : TTCAAATTTTATTTTTGGGGGCTTGGAAAATGAAGTTGTCCGTATTGTTATTAGGACTGTGATTCGCATTGTTTGTTATCTTATTTTGTATATACATTCTCCCAA : 3883
C-XNH      : TTCAAATTTTATTTTTGGGGGCTTGGAAAATGAAGTTGTCCGTGTTGTTATTAGGACTGTGATTCGCATCGTTTGTTATCTTATTTTGTATATACATTCTCCCAA : 3882
LSD/090830 : TTCAAATTTTATTTTTGGGGGCTTGGAAAATGAAGTTGTCCGTGTTGTCATCAGGACTGTGATTCGCATTGTCTGTTATCTTATTTTGTATATACATTCTCCCAA : 3884
FZ99       : TTCAAATTTTATTTTTGGGGGCTTGGAAAATGAAGTTGTCCGTGTTGTTATTAGGACTGTGATTCGCATTGTTTGTTATCTTATTTTGTATATACATTCTCCCAA : 3883
             TTCAAATTTTATTTTTGGGGGCTTGGAAAATGAAGTTGTCCGTgTTGT AT AGGACTGTGATTCGCATtGTtTGTTAtCT ATTTTGTATATACATTCTCCCAA

              *      3900         *      3920         *      3940         *      3960         *      3980         *
CH60       : CATAGTCACAACAGGGACTTTGGTTGCTTTGCTAGCTTTAGATGCCACCAGCATGTCAATGGATCAAGGACTCAAAACTCTCTGCATGAGCCTAGTGGATGGTGA : 3988
A66        : CATAGTCACAACAGGGACTTTGGTTGCTTTGCTAGCTTTAGATGCCACCAGCATGTCAATGGATCAAGGACTCAAAACTCTCTGCATGAGCCTAGTGGATGGTGA : 3988
C80        : CATAGTCACAACAGGGACTTTGGTTGCTTTGCTAGCTTTAGATGCCACCAGCATGTCAATGGATCAAGGACTCAAAACTCTCTGCATGAGCCTAGTGGATGGTGA : 3987
MY         : CATAGTCACAACAGGGACTTTGGTTGCTTTGCTAGCTTTAGATGCCACCAGCATGTCAATGGATCAAGGACTCAAAACTCTCTGCATGAGCCTAGTGGATGGTGA : 3988
FC64       : CATAGTCACAACAGGGACTTTGGTTGCTTTGCTAGCTTTAGATGCCACCAGCATGTCAATGGATCAAGGACTCAAAACTCTCTGCATGAGCCTAGTGGATGGTGA : 3988
03D        : CATAGTCACAACAGGGACTTTGGTTGCTTTGTTAGCTTTAGATGCCACCAGTATGTCAATGGATCAAGGACTCAAAACTCTCTGTATGAGCCTGGTGGATGGTGA : 3985
ZJ         : CATAGTCACAACAGGGACTTTGGTTGCTTTGTTAGCTTTAGATGCCACCAGTATGTCAATGGATCAAGGACTCAAAACTCTCTGCATGAGCCTGGTGGATGGTGA : 3985
NA         : CATAGTCACAACAGGGACTTTGGTTGCTTTGTTAGCTTTAGATGCCACCAGTATGTCAATGGATCAAGGACTCAAAACTCTCTGCATGAGCCTGGTGGATGGTGA : 3988
C-XNH      : CATAGTCACAACAGGAACTTTGGTTGCTTTGTTAGCTTTAGATGCCACCAGTATGTCAATGGATCAAGGGCTCAAAACTCTCTGCATGAGCCTGGTGGATGGTGA : 3987
LSD/090830 : CATAGTTACAACAGGGACTTTGGTTGCTTTGTTAGCTTTAGATGCCACTAGTATGTCAATGGATCAAGGACTCAAAACTCTCTGTATGAGCCTGGTGGATGGTGA : 3989
FZ99       : CATAGTCACAACAGGGACTTTGGTTGCTTTGTTAGCTTTAGATGCCACCAGTATGTCAATGGATCAAGGACTCAAAACTCTCTGCATGAGCCTAGTGGATGGTGA : 3988
             CATAGTcACAACAGGgACTTTGGTTGCTTTG TAGCTTTAGATGCCACcAG ATGTCAATGGATCAAGGaCTCAAAACTCTCTGcATGAGCCT GTGGATGGTGA

                4000         *      4020         *      4040         *      4060         *      4080         *
CH60       : TTTTGGCAAATTTTGTTCAGCTTTGTTAGAAAAAAATACAAACTGTTGACGAGGCTGATCTAAAGAAAACCATTCCAAAATTCAATGACATGTTAGAGGATCAATC : 4093
A66        : TTTTGGCAAATTTTGTTCAGCTTTGTTAGAAAAAAATACAAACTGTTGACGAGGCTGATCTAAAGAAAACCATTCCAAAATTCAATGACATGTTAGAGGATCAATC : 4093
C80        : TTTTGGCAAATTTTGTTCAGCTTTGTTAGAAAAAAATACAAACTGTTGACGAGGCTGATCTAAAGAAAACCATTCCAAAATTCAATGACATGCTAGAGGATCAATC : 4092
MY         : TTTTGGCAAATTTTGTTCAGCTTTGTTAGAAAAAAATACAAACTGTTGACGAGGCTGATCTAAAGAAAACCATTCCAAAATTCAATGACATGTTAGAGGATCAATC : 4093
FC64       : TTTTGGCAAATTTTGTTCAGCTTTGTTAGAAAAAAATACAAACTGTTGACGAGGCTGATCTAAAGAAAACCATTCCAAAATTCAATGACATGTTAGAGGATCAATC : 4093
03D        : TTTTGGCAAATTTTGTTCAGCTTTGTTAGAAAAAAATACAAATTGTTGACGAGGCTGATCTAAAGAAAACTATTCCAAAATTCAATGATATGTTAGAGGATCAATC : 4090
ZJ         : TTTTGGCAAATTTTGTTCAGCTTTGTTAGAAAAAAATACAAACTGTTGACGAGGCTGATCTAAAGAAAACTATTCCAAAATTCAATGACATGTTAGAGGATCAATC : 4090
NA         : TTTTGGCAAATTTTGTTCAGCTTTGTTAGAAAAAAATACAAACTGTTGACGAGGCTGATCTAAAGAAAACTATTCCAAAATTCAATGACATGTTAGAGGATCAATC : 4093
C-XNH      : TTTTGGCAAATTTTGTTCAGCTTTGTTAGAAAAAAATACAAACTGTTGATGAGGCTGATCTAAAAAAAACTATTCCAAAATTCAATGACATGTTAGAGGATCAATC : 4092
LSD/090830 : TTTTGGCAAATTTTGTTCAGCTTTGTTAGAAAAAAATACAAACTGTTGACGAGGCTGATCTAAAGAAAACTATTCCAAAATTCAATGGCATGTTAGAGGATCAATC : 4094
FZ99       : TTTTGGCAAATTTTGTTCAGCTTTGTTAGAAAAAAATACAAACTGTTGACGAGGCTGATCTAAAGAAAACTATTCCAAAATTCAATGACATGTTAGAGGATCAATC : 4093
             TTTTGGCAAATTTTGTTCAGCTTTGTTAGAAAAAAATACAAAcTGTTGAcGAGGCTGATCTAAAgAAAAC ATTCCAAAATTCAATGacATGtTAGAGGATCAATC

             4100         *      4120         *      4140         *      4160         *      4180         *      4200
CH60       : TGGCAAAACCACCTCTCCTAAATCTTTTAATGATTGGACAACCTGTGCCAAAAATGTACAATGGTGGCTTGAATCATTTGTCAAAGTTGTAAATTGGCTTAAAGA : 4198
A66        : TGGCAAAACCACCTCTCCTAAATCTTTTAATGATTGGACAACCTGTGCCAAAAATGTACAATGGTGGCTTGAATCATTTGTCAAAGTTGTAAATTGGCTTAAAGA : 4198
C80        : TGGCAAAACCACCTCTCCTAAATCTTTTAATGATTGGACAACCTGTGCCAAAAATGTACAATGGTGGCTTGAATCATTTGTCAAAGTTGTAAATTGGCTTAAAGA : 4197
MY         : TGGCAAAACCACCTCTCCTAAATCTTTTAATGATTGGACAACCTGTGCCAAAAATGTACAATGGTGGCTTGAATCATTTGTCAAAGTTGTAAATTGGCTTAAAGA : 4198
FC64       : TGGCAAAACCACCTCTCCTAAATCTTTTAATGATTGGACAACCTGTGCCAAAAATGTACAATGGTGGCTTGAATCATTTGTCAAAGTTGTAAATTGGCTTAAAGA : 4198
03D        : TGGCAAAGCCACCTCTCCCAAATCTTTTAATGATTGGACAACCTGTGCCAAAAATGTACAATGGTGGCTTGAATCATTTGTCAAAGTTGTAAATTGGCTTAAAGA : 4195
ZJ         : TGGCAAAGCCACCTCTCCTAAATCTTTTAATGATTGGACAACCTGTGCCAAAAATGTGCAATGGTGGCTTGAATCATTTGTCAAAGTTGTAAATTGGCTCAAAGA : 4195
NA         : TGGCAAAGCCACCTCTCCTAAATCTTTTAACGATTGGACAACCTGTGCCAAAAATGTGCAATGGTGGCTTGAATCATTTGTCAAAGTTGTAAATTGGCTCAAAGA : 4198
C-XNH      : TGGCAAAACCACCTCTCCCAAATCTTTTAATGATTGGACAACCTGTGCCAAAAATGTGCAATGGTGGCTCGAATCATTTGTCAAAGTTGTAAATTGGCTCAAAGA : 4197
LSD/090830 : TGGCAAAGCCACCTCTCCCAAATCTTTTAATGATTGGACAACCTGTGCAAAAAATGTACAATGGTGGCTTGAATCATTTGTCAAAGTTGTAAATTGGCTTAAAGA : 4199
FZ99       : TGGCAAAGCCACCTCTCCCAAATCTTTTAATGATTGGACAACCTGTGCAAAAAATGTGCAATGGTGGCTCGAATCATTTGTCAAAGTTGTAAATTGGCTCAAAGA : 4198
             TGGCAAA CCACCTCTCC AAATCTTTTAAtGATTGGACAACCTGTGCcAAAAATGT CAATGGTGGCTtGAATCATTTGTCAAAGTTGTAAATTGGCT AAAGA

                   *      4220         *      4240         *      4260         *      4280         *      4300
CH60       : AAAAATATTTCCTTCAAAAACTGACCCAACTCTTCAGTGGCTCCAGGACCACGAAGAACATATAGCTATCATGTTGGCATTGTGTGATGAGCACCTTTTGTATGCT : 4303
A66        : AAAAATATTTCCTTCAAAAACTGACCCAACTCTTCAGTGGCTCCAGGACCACGAAGAACATATAGCTATCATGTTGGCATTGTGTGATGAGCACCTTTTGTATGCT : 4303
C80        : AAAAATATTTCCTTCAAAAACTGACCCAACTCTTCAGTGGCTCCAGGACCACGAAGAACATATAGCTATCATGTTGGCATTGTGTGATGAGCACCTTTTGTATGCT : 4302
MY         : AAAAATATTTCCTTCAAAAACTGACCCAACTCTTCAGTGGCTCCAGGACCACGAAGAACATATAGCTATCATGTTGGCATTGTGTGATGAGCACCTTTTGTATGCT : 4303
FC64       : AAAAATATTTCCTTCAAAAACTGACCCAACTCTTCAGTGGCTCCAGGACCACGAAGAACATATAGCTATCATGTTGGCATTGTGTGATGAGCACCTTTTGTATGCT : 4303
03D        : AAAAGTATTTCCTTCAAAAACTGACCCAACTCTTCAGTGGCTCCAGGACCATGAAGAACATATAGCTATCATGTTGGCATTGTGTGATGAGCACCTCTGTATGCT : 4300
ZJ         : AAAAGTATTTCCTTCAAAAACTGACCCAACTCTTCAGTGGCTCCAGGACCATGAAGAACATATAGCTATCATGTTGGCATTGTGTGATGAGCACCTTTTGTATGCT : 4300
NA         : AAAAGTATTTCCTTCAAAAACTGACCCAACTCTTCAGTGGCTCCAGGACCATGAAGAACATATAGCTATCATGTTGGCATTGTGTGATGAGCACCTTTTGTATGCT : 4303
C-XNH      : AAAAGTATTTCCTTCAAAAACTGACCCAACTCTTCAGTGGCTCCAGGACCATGAAGAGCATATAGCTATCATGTTGGCATTGTGTGATGAGCACCTTTTGTATGCT : 4302
LSD/090830 : AAAAGTGTTTCCTTCAAAAACTGACCCAACTCTTCAGTGGCTCCAGGACCATGAAGAACATATAGCTATTATGTTGGCATTGTGTGATGAGCACCTTTTGTATGCT : 4304
FZ99       : AAAAGTATTTCCTTCAAAAACTGACCCAACTCTTCAGTGGCTCCAGGACCATGAAGAACATATAGCTATCATGTTGGCATTGTGTGATGAGCACCTTTTGTATGCT : 4303
             AAAA TaTTTCCTTCAAAAACTGACCCAACTCTTCAGTGGCTCCAGGACCA GAAGAaCATATAGCTATcATGTTGGCATTGTGTGATGAGCACCTTtTGTATGCT

              *      4320         *      4340         *      4360         *      4380         *      4400         *
CH60       : AAGAACTGAAAAAGATTACATTTGTGAGCACAATACTCGCCCAAAACATCAGTGTTTGGTTGAAATGGTCTCTGGCACATTGAACCAACTCCAAGGTATTTCAAG : 4408
A66        : AAGAACTGAAAAAGATTACATTTGTGAGCACAATACTCGCCCAAAACATCAGTGTTTGGTTGAAATGGTCTCTGGCACATTGAACCAACTCCAAGGTATTTCAAG : 4408
C80        : AAGAACTGAAAAAGATTACATTTGTGAGCACAATACTCGCCCAAAACATCAGTGTTTGGTTGAAATGGTCTCTGGCACATTGAACCAACTCCAAGGTATTTCAAG : 4407
MY         : AAGAACTGAAAAAGATTACATTTGTGAGCACAATACTCGCCCAAAACATCAGTGTTTGGTTGAAATGGTCTCTGGCACATTGAACCAACTCCAAGGTATTTCAAG : 4408
FC64       : AAGAACTGAAAAAGATTACATTTGTGAGCACAATACTCGCCCAAAACATCAGTGTTTGGTTGAAATGGTCTCTGGCACATTGAACCAACTCCAAGGTATTTCAAG : 4408
03D        : AAGAACTGAAAAAGATTATATTTGTGAGCACAACACTCGCCCAAAACATCAGCGTTTGGTTGAAATGGTCTCTGGTACATTGAACCAACTCCAAGGTATTTCAAG : 4405
ZJ         : AAGAACTGAAAAAGATTATATCTGTGAGCACAATACTCGCCCAAAACATCAGCGTTTGGTTGAAATGGTCTCTGGCACATTGAACCAACTCCAAGGTATTTCAAG : 4405
NA         : AAGAACTGAAAAAGATTATATCTGTGAGCACAATACTCGCCCAAAACATCAGCGTTTGGTTGAAATGGTCTCTGGCACATTGAACCAACTCCAAGGTATTTCAAG : 4408
C-XNH      : AAGAACTGAAAAAGATTATATTTGTGAACACAATACTCGCCCAAAACATCAGCGTTTGGTTGAAATGGTCTCTGGCACATTGAACCAACTCCAAGGTATTTCAAG : 4407
LSD/090830 : AAGAACTGAAAAAGATTATATTTGTGAGCACAACACTCGCCCAAAACATCAGCGTTTAGTTGAAATGGTCTCTGGTACATTGAACCAACTCCAAGGTATTTCAAG : 4409
FZ99       : AAGAACTGAAAAAGATTATATTTGTGAGCACAATACTCGCCCAAAACATCAGCGTTTGGTTGAAATGGTCTCTGGTACATTGAACCAACTCCAAGGTATTTCAAG : 4408
             AAGAACTGAAAAAGATTA ATtTGTGAgCACAAtACTCGCCCAAAACATCAG GTTTgGTTGAAATGGTCTCTGG ACATTGAACCAACTCCAAGGTATTTCAAG

                4420         *      4440         *      4460         *      4480         *      4500         *
CH60       : TGCTCGTGAATTGGCAGCTAGACTACAGCATGTGTTGAACAAACTCCACCAAGTCAATTTTGAGCCTGAACTGGAGTGGACACACCGTCCTGAACCTCTTGGTAT : 4513
A66        : TGCTCGTGAATTGGCAGCTAGACTACAGCATGTGTTGAACAAACTCCACCAAGTCAATTTTGAGCCTGAACTGGAGTGGACACACCGTCCTGAACCTCTTGGTAT : 4513
C80        : TGCTCGTGAATTGGCAGCTAGACTACAGCATGTGTTGAACAAACTCCACCAAGTCAATTTTGAGCCTGAACTGGAGTGGACACACCGTCCTGAACCTCTTGGTAT : 4512
MY         : TGCTCGTGAATTGGCAGCTAGACTACAGCATGTGTTGAACAAACTCCACCAAGTCAATTTTGAGCCTGAACTGGAGTGGACACACCGTCCTGAACCTCTTGGTAT : 4513
FC64       : TGCTCGTGAATTGGCAGCTAGACTACAGCATGTGTTGAACAAACTCCACCAAGTCAATTTTGAGCCTGAACTGGAGTGGACACACCGTCCTGAACCTCTTGGTAT : 4513
03D        : TGCTCGTGAATTGGCAGCTAGACTACAGCATGTGCTGAACAAACTCCACCAAGTCAATTTTGAACCTGAACTGGAGTGGACACACCGTCCTGAACCTCTTGGTAT : 4510
ZJ         : TGCTCGTGAATTGGCAGCTAGATTACAGCATGTGTTGAATAAACTCCACCAAGTCAATTTTGAGCCTGAACTGGAGTGGACACACCGTCCTGAACCTCTTGGTAT : 4510
NA         : TGCTCGTGAATTGGCAGCTAGATTACAGCATGTGTTGAATAAACTCCACCAAGTCAATTTTGAGCCTGAACTGGAGTGGACACACCGTCCTGAACCTCTTGGTAT : 4513
C-XNH      : TGCTCGTGAATTGGCAGCTAGATTACAGCATGTGTTGAATAAACTCCACCAAGTCAATTTTGAGCCTGAACTGGAGTGGACACACCGTCCTGAACCTCTTGGTAT : 4512
LSD/090830 : TGCCCGTGAATTGGCAGCTAGATTACAGCATGTGTTGAACAAACTCCACCAAGTCAATTTTGAACCTGAATTGGAGTGGACACACCGTCCTGAACCTCTTGGTAT : 4514
FZ99       : TGCTCGTGAATTGGCAGCTAGATTACAGCATGTGTTGAATAAACTCCACCAAGTCAATTTTGAGCCTGAACTGGAGTGGACACACCGTCCTGAACCTCTTGGTAT : 4513
             TGCtCGTGAATTGGCAGCTAGA TACAGCATGTGTtGAA AAACTCCACCAAGTCAATTTTGAgCCTGAAcTGGAGTGGACACACCGTCCTGAACCTCTTGGTAT

             4520         *      4540         *      4560         *      4580         *      4600         *      4620
CH60       : ATGGATATCTGGTGGCCCTGGTGTTGGGAAGAGCTTTTTATCAAATTACATTGTTAAAGAAATAGCCAAATTAAAGCATTGGAAGAGTTATGCCAACCCAACTGG : 4618
A66        : ATGGATATCTGGTGGCCCTGGTGTTGGGAAGAGCTTTTTATCAAATTACATTGTTAAAGAAATAGCCAAATTAAAGCATTGGAAGAGTTATGCCAACCCAACTGG : 4618
```

```
C80        : ATGGATATCTGGTGGCCCTGGTGTTGGGAAGAGCTTTTTATCAAATTACATTGTTAAAGAAATAGCCAAATTAAAGCATTGGAAGAGTTATGCCAACCCAACTGG : 4617
MY         : ATGGATATCTGGTGGCCCTGGTGTTGGGAAGAGCTTTTTATCAAATTACATTGTTAAAGAAATAGCCAAATTAAAGCATTGGAAGAGTTATGCCAACCCAACTGG : 4618
FC64       : ATGGATATCTGGTGGCCCTGGTGTTGGGAAGAGCTTTTTATCAAATTACATTGTTAAAGAAATAGCCAAATTAAAGCATTGGAAGAGTTATGCCAACCCAACTGG : 4618
03D        : ATGGATATCTGGTGGCCCTGGTGTTGGGAAGAGTTTTTTATCAAATTACATTGTTAAAGAAATAGCCAAATTAAAGCATTGGAAGAGTTATGCCAACCCAACTGG : 4615
ZJ         : ATGGATATCCGGTGGCCCTGGTGTTGGGAAGAGTTTTTTATCAAATTACATTGTTAAAGAAATAGCCAAACTAAAGCATTGGAAGAGTTATGCCAACCCAACTGG : 4615
NA         : ATGGATATCCGGTGGCCCTGGTGTTGGGAAGAGTTTTTTATCAAATTACATTGTTAAAGAAATAGCCAAACTAAAGCATTGGAAGAGTTATGCCAACCCAACTGG : 4618
C-XNH      : ATGGATATCCGGTGGCCCTGGTGTTGGGAAGAGTTTTTTATCAAATTACATTGTTAAAGAAATAGCCAAACTAAAGCATTGGAAGAGTTATGCCAACCCAACTGG : 4617
LSD/090830 : ATGGATATCTGGTGGCCCTGGTGTTGGGAAGAGTTTTTTATCAAATTATATTGTTAAAGAAATAGCCAAATTAAAACATTGGAAGAGTTATGCCAATCCAACTGG : 4619
FZ99       : ATGGATATCCGGTGGCCCTGGTGTTGGGAAGAGTTTTTTATCAAATTACATTGTTAAAGAAATAGCCAAACTAAAGCATTGGAAGAGTTATGCCAACCCAACTGG : 4618
             ATGGATATC GGTGGCCCTGGTGTTGGGAAGAG TTTTTATCAAATTAcATTGTTAAAGAAATAGCCAAA TAAAgCATTGGAAGAGTTATGCCAAcCCAACTGG

                     *        4640         *        4660         *        4680         *        4700         *        4720
CH60       : GAGTAAGCATATGGATGGGTATGTGTCACAGGAGATTCATGTTTTTGATGATTTTGGTCAAAATCGGGAAGAGGAAGACTATTCTTTAATTTGTAACCTTATTTC : 4723
A66        : GAGTAAGCATATGGATGGGTATGTGTCACAGGAGATTCATGTTTTTGATGATTTTGGTCAAAATCGGGAAGAGGAAGACTATTCTTTAATTTGTGACCTTATTTC : 4723
C80        : GAGTAAGCATATGGATGGGTATGTGTCACAGGAGATTCATGTTTTTGATGATTTTGGTCAAAATCGGGAAGAGGAAGACTATTCTTTAATTTGTAACCTTATTTC : 4722
MY         : GAGTAAGCATATGGATGGGTATGTGTCACAGGAGATTCATGTTTTTGATGATTTTGGTCAAAATCGGGAAGAGGAAGACTATTCTTTAATTTGTAACCTTATTTC : 4723
FC64       : GAGTAAGCATATGGATGGGTATGTGTCACAGGAGATTCATGTTTTTGATGATTTTGGTCAAAATCGGGAAGAGGAAGACTATTCTTTAATTTGTAACCTTATTTC : 4723
03D        : AAGTAAGCATATGGATGGGTATGTGTCACAGGAGATTCATGTTTTTGATGATTTTGGTCAAAATCGGGAAGAAGAGGACTATTCTTTAATCTGTAATCTTATTTC : 4720
ZJ         : GAGTAAGCATATGGATGGGTATGTGTCACAGGAGATTCATGTTTTTGATGATTTTGGTCAAAATCGGGAAGAAGAGGATTATTCTTTAATCTGTAACCTTATTTC : 4720
NA         : GAGTAAGCATATGGATGGGTATGTGTCACAGGAGATTCATGTTTTTGATGATTTTGGTCAAAATCGGGAAGAAGAGGATTATTCTTTAATCTGTAACCTTATTTC : 4723
C-XNH      : GAGTAAGCATATGGATGGGTATGTGTCACAGGAGATTCATGTTTTTGATGATTTTGGTCAAAATCGGGAAGAAGAGGATTATTCTTTAATCTGTAACCTTATTTC : 4722
LSD/090830 : AAGTAAGCATATGGATGGGTATGTATCACAGGAGATTCATGTTTTTGATGATTTTGGTCAGAATCGGGAAGAAGAAGACTATTCTTTAATCTGTAACCTTATTTC : 4724
FZ99       : GAGTAAGCATATGGATGGGTATGTGTCACAGGAGATTCATGTTTTTGATGATTTTGGTCAAAATCGGGAAGAAGAGGATTATTCTTTAATCTGCAACCTTATTTC : 4723
             gAGTAAGCATATGGATGGGTATGTgTCACAGGAGATTCATGTTTTTGATGATTTTGGTCAaAATCGGGAAGA GA GA TATTCTTTAAT TGTaAcCTTATTTC

                  *        4740         *        4760         *        4780         *        4800         *        4820         *
CH60       : TAGTGTTCCATTCATTACTCCAAAGGCAAGTGTAGAGGCCAAGGGCACCCAGTACAGGGGACGCCTTGTTGTTGTCACAACCAATAGACGGGATTTCACTTCTTG : 4828
A66        : TAGTGTTCCATTCATTACTCCAAAGGCAAGTGTAGAGGCCAAGGGCACCCAGTACAGGGGACGCCTTGTTGTTGTCACAACCAATAGACGGGGTTTCACTTCTTG : 4828
C80        : TAGTGTTCCATTCATTACTCCAAAGGCAAGTGTAGAGGCCAAGGGCACCCAGTACAGGGGACGCCTTGTTGTTGTCACAACCAATAGACGGGATTTCACTTCTTG : 4827
MY         : TAGTGTTCCATTCATTACTCCAAAGGCAAGTGTAGAGGCCAAGGGCACCCAGTACAGGGGACGCCTTGTTGTTGTCACAACCAATAGACGGGATTTCACTTCTTG : 4828
FC64       : TAGTGTTCCATTCATTACTCCAAAGGCAAGTGTAGAGGCCAAGGGCACCCAGTACAGGGGACGCCTTGTTGTTGTCACAACCAATAGACGGGATTTCACTTCTTG : 4828
03D        : TAGTGTTCCATTTATTACTCCAAAAGCAAGTGTAGAGGCTAAGGGCACCCAATACAGAGGACGCCTTGTTGTCGTCACAACCAATAGACGGGATTTTACTTCCTG : 4825
ZJ         : TAGTGTTCCATTCATTACTCCAAAAGCAAGTGTAGAGGCTAAGGGCACACAATACAGAGGACGCCTTGTTGTTGTCACAACCAATAGACGGGATTTCACTTCATG : 4825
NA         : TAGTGTTCCATTCATTACTCCAAAAGCAAGTGTAGAGGCTAAGGGCACACAATACAGAGGACGCCTTGTTGTTGTCACAACCAATAGACGGGATTTCACTTCATG : 4828
C-XNH      : TAGTGTTCCATTCATTACTCCAAAAGCAAGTGTAGAGGCTAAGGGCACTCAATACAGGGGACGCCTTGTTGTTGTCACAACCAATAGACGGGATTTCACTTCCTG : 4827
LSD/090830 : TAGTGTTCCATTCATTACTCCAAAAGCAAGTGTAGAGGCTAAGGGCACCCAATATAGAGGACGCCTTGTTGTCGTCACAACCAATAGACGGGATTTCACTTCCTG : 4829
FZ99       : TAGTGTTCCATTCATTACTCCAAAAGCAAGTGTAGAGGCTAAGGGCACTCAATACAGAGGACGCCTTGTTGTTGTCACAACCAATAGACGGGATTTCACTTCCTG : 4828
             TAGTGTTCCATTcATTACTCCAAA GCAAGTGTAGAGGC AAGGGCAC CA TAcAG GGACGCCTTGTTGTtGTCACAACCAATAGACGGGaTTTcACTTC TG

                   4840         *        4860         *        4880         *        4900         *        4920         *
CH60       : CAAGTTGACTGACCCAGATGCATTGGAAAGGCGTTTCCCTATTCGCCTTAATATTCGGCCATTGCAAAAATATAACCACAAGGGCCGTTTGGATGTGGCAACAGC : 4933
A66        : CAAGTTGACTGACCCAGAAGCATTGGAAAGGCGTTTCCCTATTCGCCTTAATATTCGGCCATTGCAAAAATATAACCACAAGGGCCGTTTGGATGTGGCAACAGC : 4933
C80        : CAAGTTGACTGACCCAGATGCATTGGAAAGGCGTTTCCCTATTCGCCTTAATATTCGGCCATTGCAAAAATATAACCACAAGGGCCGTTTGGATGTGGCAACAGC : 4932
MY         : CAAGTTGACTGACCCAGATGCATTGGAAAGACGTTTCCCTATTCGCCTTAATATTCGGCCATTGCAAAAATATAACCACAAGGGCCGTTTGGATGTGGCAACAGC : 4933
FC64       : CAAGTTGACTGACCCAGATGCATTGGAAAGGCGTTTCCCTATTCGCCTTAATATTCGGCCATTGCAAAAATATAACCACAAGGGCCGTTTGGATGTGGCAACAGC : 4933
03D        : TAAGTTGACTGACCCAGATGCACTGGAAAGGCGCTTTCCTATTCGCCTTAATATCCGGCCATTGCAAAAGTATAATCACAAGGGCCGTTTGGATGTGGCAACAGC : 4930
ZJ         : TAAGTTGACTGACCCAGATGCACTGGAAAGGCGCTTTCCTATTCGCCTTAATATCCGGCCATTGCAAAAGTATAACCACAAGGGCCGTTTGGATGTGGCAACAGC : 4930
NA         : TAAGTTGACTGACCCAGATGCACTGGAAAGGCGCTTTCCTATTCGCCTTAATATCCGGCCATTGCAAAAGTATAACCACAAGGGCCGTTTGGATGTGGCAACAGC : 4933
C-XNH      : TAAGTTGACTGACCCAGATGCACTGGAAAGACGCTTTCCTATTCGCCTTAATATCCGGCCATTGCAAAAGTATAACCACAAGGGCCGTTTGGATGTGGCAACAGC : 4932
LSD/090830 : TAAGTTGACTGACCCAGATGCACTGGAAAGGCGCTTTCCTTTTCGCCTTAACATTCGGCCATTGCAAAAGTACAACCATAAGGGCCGTTTGGATGTGGCAACAGC : 4934
FZ99       : TAAGTTGACTGACCCAGATGCACTGGAAAGGCGCTTTCCTATTCGCCTTAATATCCGGCCATTGCAAAAGTATAACCACAAGGGCCGTTTGGATGTGGCAACAGC : 4933
              AAGTTGACTGACCCAGAtGCA TGGAAAGgCG TT CCTaTTCGCCTTAAtAT CGGCCATTGCAAAA TAtAAcCAcAAGGGCCGTTTGGATGTGGCAACAGC

              4940         *        4960         *        4980         *        5000         *        5020         *        5040
CH60       : TATGAGAGATGGAAGCTTGCAGGGAGGAGCTTGCTGGGAAAGGGATATAGGCCAATTAGGCCTTGAATGTTGGAATCCTATAAATGGCCAAACCCTGATTGATGA : 5038
A66        : TATGAGAGATGGAAGCTTGCAGGGAGGAGCTTGCTGGGAAAGGGATATAGGCCAATTAGGCCTTGAATGTTGGAATCCTATAAATGGCCAAACCCTGATTGATGA : 5038
C80        : TATGAGAGATGGAAGCTTGCAGGGAGGAGCTTGCTGGGAAAGGGATATAGGCCAATTAGGCCTTGAATGTTGGAATCCTATAAATGGCCAAACCCTGATTGATGA : 5037
MY         : TATGAGAGATGGAAGCTTGCAGGGAGGAGCTTGCTGGGAAAGGGATATAGGCCAATTAGGCCTTGAATGTTGGAATCCTATAAATGGCCAAACCCTGATTGATGA : 5038
FC64       : TATGAGAGATGGAAGCTTGCAGGGAGGAGCTTGCTGGGAAAGGGATATAGGCCAATTAGGCCTTGAATGTTGGAATCCTATAAATGGCCAAACCCTGATTGATGA : 5038
03D        : CATGAGAGATGGAAGCTTGCAGGGAGGAACTTGCTGGGAAAGGGATATAGGCCAATTAGGTCTTGAATGTTGGAATCCCATAAATGGCCAAATCTTAATTGATGA : 5035
ZJ         : CATGAGAGATGGAAGCTTGCAGGGAGGAACTTGCTGGGAAAGGGATATAGGCCAATTAGGTCTTGAATGTTGGAATCCCATAAATGGCCAAACCCTAATTGATGA : 5035
NA         : CATGAGAGATGGAAGCTTGCAGGGAGGAACTTGCTGGGAAAGGGATATAGGCCAATTAGGTCTTGAATGTTGGAATCCCATAAATGGCCAAACCCTAATTGATGA : 5038
C-XNH      : CATGAGAGATGGAAGCTTGCAGGGAGGAACTTGCTGGGAAAGGGATATAGGCCAATTAGGTCTTGAATGTTGGAATCCCATAAATGGCCAAACCCTAATTGATGA : 5037
LSD/090830 : CATGAGAGATGGAAGCTTGCAGGGAGGAACTTGCTGGGAAAGGGATATAGGCCAATTAGGTCTTGAATGCTGGAATCCCATAAATGGCCAAACCCTAATTGATGA : 5039
FZ99       : CATGAGAGATGGAAGCTTGCAGGGAGGAACTTGCTGGGTAAGGGATATAGGCCAATTAGGTCTTGAATGTTGGAATCCCATAAATGGCCAAACCCTAATTGATGA : 5038
              ATGAGAGATGGAAGCTTGCAGGGAGGA CTTGCTGGGaAAGGGATATAGGCCAATTAGG CTTGAATGtTGGAATCC ATAAATGGCCAAAcCcT ATTGATGA

                     *        5060         *        5080         *        5100         *        5120         *        5140
CH60       : AATTATGAGTGAGTTGCAGGTTAGGCAAGAAGTAGCTTCCTTTATGAACCAGTCTAAGGTGAGGCGTTTCTCTGACCCGGAGACTCTTTTTAGTGATTTGGAAGA : 5143
A66        : AATTATGAGTGAGTTGCAGGTTAGGCAAGAAGTAGCTTCCTTTATGAACCAGTCTAAGGTGAGGCGTTCCTCTGACCCGGAGACTCTTTTCAGTGATTTGGAAGA : 5143
C80        : AATTATGAGTGAGTTGCAGGTTAGGCAAGAAGTAGCTTCCTTTATGAACCAGTCTAAGGTGAGGCGTTTCTCTGACCCGGAGACTCTTTTTAGTGATTTGGAAGA : 5142
MY         : AATTATGAGTGAGTTGCAGGTTAGGCAAGAAGTAGCTTCCTTTATGAACCAGTCTAAGGCGAGGCGTTTCTCTGACCCGGAGACTCTTTTTAGTGATTTGGAAGA : 5143
FC64       : AATTATGAGTGAGTTGCAGGTTAGGCAAGAAGTAGCTTCCTTTATGAACCAGTCTAATGTGAGGCGTTTCTCTGACCCGGAGACTCTTTTTAGTGATTTGGAAGA : 5143
03D        : AATTATGAGTGAGTTGCAGGTTAGGCAAGAAGTGGCTTCCTTTATGAACCAGTCCAAGGTGAGGCGTTTTTCTGACCCGGAGACACTTTTTAGTGATTTGGAAGA : 5140
ZJ         : AATTATGAGTGAGTTGCAGGTCAGGCAAGAAGTAGCTTCCTTTATGAACCAGTCAAAGGTGAGGCGTTTCTCTGACCCAGAGACACTTTTTAGTGACTTGGAAGA : 5140
NA         : AATTATGAGTGAGTTGCAGGTCAGGCAAGAAGTAGCTTCCTTTATGAACCAGTCAAAGGTGAGGCGTTTCTCTGACCCAGAGACACTTTTTAGTGACTTGGAAGA : 5143
C-XNH      : AATTATGAGTGAGTTGCAGGTTAGGCAAGAAGTAGCTTCCTTTATGAACCAGTCTAAGGTGAGGCGTTTCTCTGACCCGGAGACACTTTTTAGTGATTTGGAGGA : 5142
LSD/090830 : AATTATGAGTGAGTTGCAGGTTAGGCAAGAAGTAGCTTCCTTTATGAATCAGTCTAAGGTGAGGCGCTTCTCTGACCCGGAGACACTTTTTAGTGATTTGGAAGA : 5144
FZ99       : AATTATGAGTGAGTTGCAGGTTAGGCAAGAAGTAGCTTCCTTTATGAACCAGTCTAAGGTGAGGCGTTTTTCTGACCCGGAGACACTTTTTAGTGATTTGGAAGA : 5143
             AATTATGAGTGAGTTGCAGGTtAGGCAAGAAGTaGCTTCCTTTATGAAcCAGTC AAgGtGAGGCGtTtcTCTGACCCgGAGAC CTTTTtAGTGAtTTGGAaGA

                  *        5160         *        5180         *        5200         *        5220         *        5240         *
CH60       : TCTAAAATTGGAATTTGATTTTGATCAACTCGAGCAACAAGCTAAACTTTTTGCAAAACCAAAAGAAGGAAAAATCTCTAAATTTAGGGCTTGGGTTAGAGACTG : 5248
A66        : TCTAAAATTGGAATTTGATTTTGATCAACTCGAGCAACAAGCTAAACTTTTTGCAAAACCAAAAGAAGGAAAAATCTCTAAATTTAGGGCTTGGGTTAGAGACTG : 5248
C80        : TCTAAAATTGGAATTTGATTTTGATCAACTCGAGCAACAAGCTAAACTTTTTGCAAAACCAAAAGAAGGAAAAATCTCTAAATTTAGGGCTTGGGTTAGAGACTG : 5247
MY         : TCTAAAATTGGAATTTGATTTTGATCAACTCGAGCAACAAGCTAAACTTTTTGCAAAACCAAAAGAAGGAAAAATCTCTAAATTTAGGGCTTGGGTTAGAGACTG : 5248
FC64       : TCTAAAATTGGAATTTGATTTTGATCAACTCGAGCAACAAGCTAAACTTTTTGCAAAACCAAAAGAAGGAAAAATCTCTAAATTTAGGGCTTGGGTTAGAGACTG : 5248
03D        : TCTAAAATTGGAATTTGATTTTGATCAATTGGAGCAACAAGCTAAACTTTTTGCAAAACCAAAAGAAGGAAAAATCTCTAAATTTAGGGCCTGGGTTAGAGACTG : 5245
ZJ         : TCTAAAATTGGAATTTGATTTTGATCAATTGGAGCAGCAAGCTAAACTTTTTGCAAAACCAAAAGAAGGAAAAATCTCTAAATTTAGGGCTTGGGTTAGAGACTG : 5245
NA         : TCTAAAATTGGAATTTGATTTTGATCAATTGGAGCAGCAAGCTAAACTTTTTGCAAAACCAAAAGAAGGAAAAATCTCTAAATTTAGGGCTTGGGTTAGAGACTG : 5248
C-XNH      : TCTAAAATTGGAATTTGATTTTGATCAATTGGAGCAACAAGCTAAACTTTTTGCAAAACCAAAAGAAGGAAAAATCTCTAAATTTAGGGCCTGGGTTAGAGACTG : 5247
LSD/090830 : TCTAAAATTGGAATTTGATTTTGATCAATTGGAGCAACAAGCTAAACTTTTTGCAAAACCAAAAGAAGGAAAAATCTCTAAATTTAGGGCTTGGGTTAGAGACTG : 5249
FZ99       : TCTAAAATTGGAATTTGATTTTGATCAATTGGAGCAACAAGCTAAACTTTTTGCAAAACCAAAAGAAGGAAAAATCTCTAAATTTAGGGCCTGGGTTAAAGACTG : 5248
             TCTAAAATTGGAATTTGATTTTGATCAA T GAGCAaCAAGCTAAACTTTTTGCAAAACCAAAAGAAGGAAAAATCTCTAAATTTAGGGC TGGGTTAgAGACTG

                   5260         *        5280         *        5300         *        5320         *        5340         *
CH60       : CACAGGGAAGATTAAGGGTTTTCTGGAGCGGAATCGGGCTTGGATTCTTGGTATAGGAACCCTTGGAACCATCATGTCACTTGTGACCATGTGTGTCCCATTAGC : 5353
A66        : CACAGGGAAGATTAAGGGTTTTCTGGAGCGGAATCGGGCTTGGATTCTTGGTATAGGAACCCTTGGAACCATCAAGTCACTTGTGACCATGTGTGTCCCATTAGC : 5353
C80        : CACAGGGAAGATTAAGGGTTTTCTGGAGCGGAATCGGGCTTGGATTCTTGGTATAGGAACCCTTGGAACCATCATGTCACTTGTGACCATGTGTGTCCCATTAGC : 5352
MY         : CACAGGGAAGATTAAGGGTTTTCTGGAGCGGAATCGGGCTTGGATTCTTGGTATAGGAACCCTTGGAACCATCATGTCACTTGTGACCATGTGTGTCCCATTAGC : 5353
FC64       : CACAGGGAAGATTAAGGGTTTTCTGGAGCGGAATCGGGCTTGGATTCTTGGTATAGGAACCCTTGGAACCATCATGTCACTTGTGACCATGTGTGTCCCATTAGC : 5353
```

```
03D        : TACAGGGAAGATTAAGGGTTTTCTGGAGCGAAATCGGGCTTGGATTCTTGGTATAGGAACCCTTGGAACCATTATGTCACTTGTGACTATGTGTGTCCCATTAGC : 5350
ZJ         : TACAGGGAAGATTAAGGGTTTTCTGGAGCGAAATCGGGCTTGGATTCTTGGTATAGGAACCCTTGGAACCATTATGTCACTTGTGACCATGTGTGTCCCATTAGC : 5350
NA         : TACAGGGAAGATTAAGGGTTTTCTGGAGCGAAATCGGGCTTGGATTCTTGGCATAGGAACCCTTGGAACCATTATGTCACTAGTGACCATGTGTGTCCCATTAGC : 5353
C-XNH      : TACAGGGAAGATTAAGGGTTTTCTGGAGCGAAATCGGGCTTGGATTCTTGGTATAGGAACCCTTGGAACCATTATGTCACTAGTGACTATGTGTGTCCCATTAGC : 5352
LSD/090830 : CACAGGGAAGATTAAGGGTTTTCTGGAGCGAAATCGGGCTTGGATTCTTGGTATAGGAACCCTTGGAACCATTATGTCACTTGTGACCATGTGTGTCCCATTAGC : 5354
FZ99       : TACAGGGAAGATTAAGGGTTTTCTGGAGCGAAATCGGGCTTGGATTCTTGGTATAGGAACCCTAGGAACCATTATGTCACTAGTGACCATGTGTGTTCCATTAGC : 5353
              ACAGGGAAGATTAAGGGTTTTCTGGAGCG AATCGGGCTTGGATTCTTGGtATAGGAACCCTtGGAACCAT AtGTCACT GTGACcATGTGTGTcCCATTAGC

                5360         *        5380         *        5400         *        5420         *        5440         *        5460
CH60       : TAGACGCTTTGCTCAGTCAATCTATTCACAACAACCTGTGGCCAAAACACTCCCTAAAGACTTTAAGGTAGCTGTTAAGAAACACATTGAGAAATTAGAAACTGG : 5458
A66        : TAGACGCTTTGCTCAGTCAATCTATTCACAACAACCTGTGGCCAAAACACTCCCTAAAGACTTTAAGGTAGCTGTTAAGAAACACATTGAGAAATTAGAAACTGG : 5458
C80        : TAGACGCTTTGCTCAGTCAATCTATTCACAACAACCTGTGGCCAAAACACTCCCTAAAGACTTTAAGGTAGCTGTTAAGAAACACATTGAGAAATTAGAAACTGG : 5457
MY         : TAGACGCTTTGCTCAGTCAATCTATTCACAACAACCTGTGGCCAAAACACTCCCTAAAGACTTTAAGGTAGCTGTTAAGAAACACATTGAGAAATTAGAAACTGG : 5458
FC64       : TAGACGCTTTGCTCAGTCAATCTATTCACAACAACCTGTGGCCAAAACACTCCCTAAAGACTTTAAGGTAGCTGTTAAGAAACACATTGAGAAATTAGAAACTGG : 5458
03D        : TAGACGCTTTGCCCAGTCGATCTATTCACAACAACCTGTGGCCAAAACACTCCCTAAAGATTTTAAGGTAGCTGTTAAGAAACACATTGAAAAACTAGAAACTGA : 5455
ZJ         : TAGGCGCTTTGCCCAGTCAATCTATTCACAACAACCTGTGGCAAAAACACTCCCTAAAGATTTTAAGGTAGCTGTTAAGAAACACATTGATAAACTAGAAACTGA : 5455
NA         : TAGGCGCTTTGCCCAGTCAATCTATTCACAACAACCTGTGGCAAAAACACTCCCTAAAGATTTTAAGGTAGCTGTTAAGAAACACATTGATAAACTAGAAACTGA : 5458
C-XNH      : TAGGCGCTTTGCCCAGTCGATCTATTCACAACAACCTGTGGCAAAAACACTCCCTAAAGATTTTAAGGTAGCTGTCAAGAAACACATTGATAAACTAGAAACTGA : 5457
LSD/090830 : GAGACGCTTTGCCCAGTCGATCTATTCACAACAACCTGTGGCCAAAACACTCCCTAAAGATTTTAAGGTAGCTGTTAAGAAACACATTGAAAAACTAGAAACTGG : 5459
FZ99       : TAGGCGCTTTGCCCAGTCGATCTATTCACAACAACCTGTGGCAAAAACACTCCCTAAAGATTTTAAGGTAGCTGTTAAGAAACACATTGATAAACTAGAAACTGA : 5458
             tAG CGCTTTGC CAGTC ATCTATTCACAACAACCTGTGGC AAAACACTCCCTAAAGA TTTAAGGTAGCTGTtAAGAAACACATTGA AAA TAGAAACTG

                       *        5480         *        5500         *        5520         *        5540         *        5560
CH60       : ATTGGAAGACCAGAGCGGGCGGGTGAATTTCAGACATATTTGCAATAGACTCGTCAATGTGAGTAATGAGGATGAAGTAGCCACTGGTTTAGCCATTGGTGGGAA : 5563
A66        : ATTGGAAGACCAGAGCGGGCGGGTGAATTTCAGACATATTTGCAATAGACTCGTCAATGTGAGTAATGAGGATGAAGTAGCCACTGGTTTAGCCATTGGTGGGAA : 5563
C80        : ATTGGAAGACCAGAGCGGGCGGGTGAATTTCAGACATATTTGCAATAGACTCGTCAATGTGAGTAATGAGGATGAAGTAGCCACTGGTTTAGCCATTGGTGGGAA : 5562
MY         : ATTGGAAGACCAGAGCGGGCGGGTGAATTTCAGACATATTTGCAATAGACTCGTCAATGTGAGTAATGAGGATGAAGTAGCCACTGGTTTAGCCATTGGTGGGAA : 5563
FC64       : ATTGGAAGACCAGAGCGGGCGGGTGAATTTCAGACATATTTGCAATAGACTCGTCAATGTGAGTAATGAGGATGAAGTAGCCACTGGTTTAGCCATTGGTGGGAA : 5563
03D        : ATTGGAAGATCAGAGCGGGCGGGTGAATTTTAGACATATTTGTAACAGACTCGTCAATGTGAGTAATGAGGATGAAGTAGCCACTGGTTTAGCCATTGGTGGGAA : 5560
ZJ         : ATTGGAAGATCAGAGCGGGCGGGTAAATTTCAGACATATTTGTAACAGACTCGTCAATGTAAGCAATGAGAATGAAGTAGCCACTGGTTTAGCCATTGGTGGGAA : 5560
NA         : ATTGGAGGATCAGAGCGGGCGGGTAAATTTCAGACATATTTGTAACAGACTCGTCAATGTAAGCAATGAGAATGAAGTAGCCACTGGTTTAGCCATTGGTGGGAA : 5563
C-XNH      : ATTGGAAGATCAGAGCGGGCGGGTGAATTTCAGACATATCTGTAACAGACTCGTCAATGTGAGTAATGAGAATGAAGTAGCTACTGGTTTAGCCATTGGTGGGAA : 5562
LSD/090830 : ATTGGAAGATCAGAGCGGGCGGGTGAATTTCAGACATATTTGTAATAGACTCGTTAATGTGAGTAATGAGGATGAAGTAGCCACTGGTTTAGCCATTGGTGGGAA : 5564
FZ99       : ATTGGAAGATCAGAGCGGGCGGGTGAATTTCAGACATATCTGTAACAGACTCGTCAATGTGAGTAATGAGGATGAAGTAGCCACTGGTTTAGCCATTGGTGGGAA : 5563
             ATTGGAaGA CAGAGCGGGCGGGTgAATTTcAGACATATtTG AA AGACTCGTcAATGTgAGtAATGAG ATGAAGTAGCcACTGGTTTAGCCATTGGTGGGAA

                 *        5580         *        5600         *        5620         *        5640         *        5660         *
CH60       : AAATGTCTTGACATTTGGGCATTCTAAATTTACACAATTGGATGAGATTAGGGACATGCAATTCAATGCACCAGCTAAGGGGACCCCCATTACTTATGATGGAGA : 5668
A66        : AAATGTCTTGACATTTGGGCATTCTAAATTTACACAATTGGATGAGATTAGGGACATGCAATTCAATGCACCAGCTAAGGGGACCCCCATTACTTATGATGGAGA : 5668
C80        : AAATGTCTTGACATTTGGGCATTCTAAATTTACACAATTGGATGAGATTAGGGACATGCAATTCAATGCACCAGCTAAGGGGACCCCCATTACTTATGATGGAGA : 5667
MY         : AAATGTCTTGACATTTGGGCATTCTAAATTTACACAATTGGATGAGATTAGGGACATGCAATTCAATGCACCAGCTAAGGGGACCCCCATTACTTATGATGGAGA : 5668
FC64       : AAATGTCTTGACATTTGGGCATTCTAAATTTACACAATTGGATGAGATTAGGGACATGCAATTTAATGCACCAGCTAAGGGAACCCCCATTACTTATGATGGAGA : 5668
03D        : AAATGTTTTGACATTTGGGCATTCTAAATTTACACAATTGGATGAGATTAGGGACATTCAATTTAATGCGCCAGCTAAGGGGACCCCCATTACTTATGATGGAGA : 5665
ZJ         : AAATGTTTTGACATTTGGGCATTCTAAATTTACACAATTGGATGAGATTAGGGATATGCAATTTAATGCGCCAGCTAAGGGGACCCCCATTACTTATGATGGAGA : 5665
NA         : AAATGTTTTAACATTTGGGCATTCTAAATTTACACAATTGGATGAGATTAGGGATATGCAATTTAATGCGCCAGCTAAGGGGACCCCCATTACTTATGATGGAGA : 5668
C-XNH      : AAATGTCTTGACATTTGGACATTCTAAATTCACACAATTAGATGAGATTAGGGATATGCAATTTAATGCGCCAGCTAAGGGGACCCCCATTACTTATGATGGAGA : 5667
LSD/090830 : AAATGTCTTGACATTTGGGCATTCTAAATTTACACAATTGGATGAGATTAGAGACATGCAGTTTAACGGGCCAGTTAAGGGGACCCCCATTACTTATGATGGAGA : 5669
FZ99       : AAATGTCTTGACATTTGGGCATTCTAAATTTACACAATTGGATGAGATTAGGGATATGCAATTTAATGCGCCAGCTAAGGGGACCCCCATTACTTATGATGGAGA : 5668
             AAATGT TTgACATTTGGgCATTCTAAATTtACACAATTgGATGAGATTAGgGA ATgCAaTT AAtGC CCAGcTAAGGGGACCCCCATTACTTATGATGGAGA

                      5680         *        5700         *        5720         *        5740         *        5760         *
CH60       : ACCCACTGATCTCCAGTTATTGGAATGTGATATACCCCATCAGTTCAAAGATGTGTCTAAATTGATTGCCACGGAGGACTATCGCGGTAATGGTTGGTTGGTTTG : 5773
A66        : ACCCACTGATCTCCAGTTATTGGAATGTGATATACCCCATCAGTTCAAAGATGTGTCTAAATTGATTGCCACGGAGGACTATCGCGGTAATGGTTGGTTGGTTTG : 5773
C80        : ACCCACTGATCTCCAGTTATTGGAATGTGATATACCCCATCAGTTCAAAGATGTGTCTAAATTGATTGCCACGGAGGACTATCGCGGTAATGGTTGGTTGGTTTG : 5772
MY         : ACCCACTGATCTCCAGTTATTGGAATGTGACATACCCCATCAGTTCAAAGATGTGTCTAAATTGATTGCCACGGAGGACTATCGCGGTAATGGTTGGTTGGTTTG : 5773
FC64       : ACCCACTGATCTCCAGTTATTGGAATGTGATATACCCCATCAGTTCAAAGATGTGTCTAAATTGATTGCCACGGAGGACTATCGCGGTAATGGTTGGTTGGTTTG : 5773
03D        : ACCCACTGATCTCCAGTTGTTGGAATGTGACATACCCCATCAGTTTAAAGATGTGTCTAAATTGATTGCCACAGAGGACTATCGCGGCAATGGCTGGTTGGTTTG : 5770
ZJ         : ACCCACTGATCTCCAGTTGTTGGAATGTGACATACCCCATCAGTTTAAAGATGTGTCTAAATTGATTGCCACAGAGGACTATCGCGGCAATGGCTGGTTGGTTTG : 5770
NA         : ACCCACTGATCTCCAGTTGTTGGAATGTGACATACCCCATCAGTTTAAAGATGTGTCTAAATTGATTGCCACAGAGGACTATCGCGGCAATGGCTGGTTGGTTTG : 5773
C-XNH      : ACCCACTGATCTCCAGTTGTTGGAATGTGACATACCCCATCAGTTTAAAGATGTGTCTAAATTGATTGCCACAGAGGACTATCGCGGCAATGGCTGGTTGGTTTG : 5772
LSD/090830 : ACCCACTGATCTCCAGTTGTTGGAATGTGACATACCCCATCAGTTTAAAGATGTGTCTAAATTGATTGCCACAGAGGATTATCGCGGCAATGGCTGGTTGGTTTG : 5774
FZ99       : ACCCACTGATCTCCAGTTGTTGGAATGTGACATACCCCATCAGTTTAAAGATGTGTCTAAATTGATTGCCACAGAGGACTATCGCGGTAATGGCTGGTTGGTTTG : 5773
             ACCCACTGATCTCCAGTT TTGGAATGTGA ATACCCCATCAGTT AAAGATGTGTCTAAATTGATTGCCAC GAGGAcTATCGCGG AATGG TGGTTGGTTTG

                5780         *        5800         *        5820         *        5840         *        5860         *        5880
CH60       : GAAAGATCAGGACCAGTATATGGTGCAAGATGTGACAAAGATTAGGCCTTTTGGTTCCACAACAACTGCATCAGGCACAACATCATGTCAAACATATATATATAA : 5878
A66        : GAAAGATCAGGACCAGTATATGGTGCAAGATGTGACAAAGATTAGGCCTTTTGGTTCCACAACAACTGCATCAGGCACAACATCATGTCAAACATATATATATAA : 5878
C80        : GAAAGATCAGGACCAGTATATGGTGCAAGATGTGACAAAGATTAGGCCTTTTGGTTCCACAACAACTGCATCAGGCACAACATCATGTCAAACATATATATATAA : 5877
MY         : GAAAGATCAGGACCAGTATATGGTGCAAGATGTGACAAAGATTAGGCCTTTTGGTTCCACAACAACTGCATCAGGCACAACATCATGTCAAACATATATATATAA : 5878
FC64       : GAAAGATCAGGACCAGTATATGGTGCAAGATGTGACAAAGATTAGGCCTTTTGGTTCCACAACAACTGCATCAGGCACAACATCATGTCAAACATATATATATAA : 5878
03D        : GAAAGATCAGGACCAGTATATGGTGCAAGATGTGACAAAGATTAGGCCTTTTGGTTCCACAACAACTGCATCAGGCACGACATCATGTCAAACATACATATATAA : 5875
ZJ         : GAAAGATCAGGACCAGTATATGGTGCAAGATGTGACAAAGATTAGGCCTTTTGGTTCCACAACAACTGCATCAGGCACGACATCATGTCAAACATACATATACAA : 5875
NA         : GAAAGATCAGGACCAGTATATGGTGCAAGATGTGACAAAGATTAGGCCTTTTGGTTCCACAACAACTGCATCAGGCACGACATCATGTCAAACATACATATATAA : 5878
C-XNH      : GAAAGATCAGGACCAGTATATGGTACAAGATGTGACAAAGATTAGGCCTTTTGGTTCCACGACAACTGCATCAGGCACGACATCATGTCAAACATACATATATAA : 5877
LSD/090830 : GAAAGATCAGGACCAGTACATGGTGCAAGATGTGACAAAGATTAGGCCTTTTGGTTCCACAACAACTGCATCAGGCACGACATCATGTCAAACATACATATATAA : 5879
FZ99       : GAAAGATCAGGACCAGTATATGGTGCAAGATGTGACAAAGATTAGGCCTTTTGGTTCCACAACAACTGCATCAGGCACGACATCATGTCAAACATACATATATAA : 5878
             GAAAGATCAGGACCAGTAtATGGTgCAAGATGTGACAAAGATTAGGCCTTTTGGTTCCACaACAACTGCATCAGGCAC ACATCATGTCAAACATA ATATAtAA

                       *        5900         *        5920         *        5940         *        5960         *        5980
CH60       : TTGCAAGACTGGGCCTGGCTCCTGTGGTGGTGTGCTTGTAGCTCAAATTGGAGGAAATTTGAAGATCCTTGGGATACATACAAGTGGCAATGGCACTATGGGAGC : 5983
A66        : TTGCAAGACTGGGCCTGGCTCCTGTGGTGGCGTGCTTGTAGCTCAAATTGGAGGAAATTTGAAGATCCTTGGGATACATACAAGTGGCAATGGCACTATGGGAGC : 5983
C80        : TTGCAAGACTGGGCCTGGCTCCTGTGGTGGTGTGCTTGTAGCTCAAATTGGAGGAAATTTGAAGATCCTTGGGATACATACAAGTGGCAATGGCACTATGGGAGC : 5982
MY         : TTGCAAGACTGGGCCTGGCTCCTGTGGTGGTGTGCTTGTAGCTCAAATTGGAGGAAATTTGAAGATCCTTGGGATACATACAAGTGGCAATGGCACTATGGGAGC : 5983
FC64       : TTGCAAGACTGGGCCTGGCTCCTGTGGTGGTGTGCTTGTAGCTCAAATTGGAGGAAATTTGAAGATCCTTGGGATACATACAAGTGGCAATGGCACTATGGGAGC : 5983
03D        : TTGCAAGACTGGGCCTGGTTCCTGTGGTGGTGTGCTTGTAGCTCAAATTGGAGGAAATTTGAAGATCCTTGGGATACACACAAGTGGCAATGGCACTATGGGAGC : 5980
ZJ         : TTGCAAGACTGGGCCTGGTTCTTGTGGTGGTGTGCTTGTGGCTCAAATTGGAGGAAATTTGAAGATCCTTGGGATACACACAAGTGGCAATGGCACTATGGGAGC : 5980
NA         : CTGCAAGACTGGGCCTGGTTCTTGTGGTGGTGTGCTTGTGGCTCAAATTGGAGGAAATTTGAAGATCCTTGGGATACACACAAGTGGCAATGGCACTATGGGAGC : 5983
C-XNH      : TTGCAAGACTGGGCCTGGTTCCTGTGGTGGTGTGCTTGTAGCTCAAATTGGAGGAAATTTGAAGATCCTTGGGATACATACAAGTGGCAATGGTACTATGGGAGC : 5982
LSD/090830 : TTGCAAGACTGGGCCTGGTTCCTGTGGTGGTGTGCTTGTAGCTCAAATTGGAGGAAATTTGAAGATCCTTGGGATACACACAAGTGGCAATGGCACTATGGGAGC : 5984
FZ99       : TTGCAAGACTGGGCCTGGTTCCTGTGGTGGTGTGCTTGTAGCTCAAATTGGAGGAAATTTGAAGATCCTTGGGATACACACAAGTGGCAATGGCACTATGGGAGC : 5983
             tTGCAAGACTGGGCCTGG TCcTGTGGTGGtGTGCTTGTaGCTCAAATTGGAGGAAATTTGAAGATCCTTGGGATACA ACAAGTGGCAATGGcACTATGGGAGC

                 *        6000         *        6020         *        6040         *        6060         *        6080         *
CH60       : AGCCAATAGGGTCTTTCCAGTTTTTAATCAAGGGAAAGTAGTGAGCAAGCAATATGCAGGTAAGATTCTGTACCATCAACCCCGAAAAACTGCATATATGAAGTC : 6088
A66        : AGCCAATAGGGTCTTTCCAGTTTTTAATCAAGGGAAAGTAGTGAGCAAACTATATGCAGGTAAGATTCTGTACCATCAACCCCGAAAAACTGCATATATGAAGTC : 6088
C80        : AGCCAATAGGGTCTTTCCAGTTTTTAATCAAGGGAAAGTAGTGAGCAAGCAATATGCAGGTAAGATTCTGTACCATCAACCCCGAAAAACTGCATATATGAAGTC : 6087
MY         : AGCCAATAGGGTCTTTCCAGTTTTTAATCAAGGGAAAGTAGTGAGCAAGCAATATGCAGGTAAGATTCTGTACCATCAACCCCGAAAAACTGCATATATGAAGTC : 6088
FC64       : AGCCAATAGGGTCTTTCCAGTTTTTAATCAAGGGAAAGTAGTGAGCAAGCAATATGCAGGTAAGATTCTGTACCATCAACCCCGAAAAACTGCATATATGAAGTC : 6088
03D        : AGCCAATAGGGTCTTTCCAGTTTTTAATCAAGGCAAAGTAGTGAGCAAGCAATATGCAGGGAAGATTCTGTACCATCAACCCCGAAAAACTGCATATATGAAGTC : 6085
ZJ         : AGCCAATAGGGTCTTTCCAGTTTTTAATCAAGGCAAAGTAGTGAGTAAGCAATATGCAGGGAAGATTCTGTACCATCAACCCCGAAAAACTGCATATATGAAGTC : 6085
NA         : AGCCAATAGGGTCTTTCCAGTTTTTAATCAAGGCAAAGTAGTGAGTAAGCAATATGCAGGGAAAATTCTGTACCATCAACCCCGAAAAACTGCATATATGAAGTC : 6088
```

```
C-XNH      : AGCCAATAGGGTCTTCCCAGTTTTTAATCAAGGCAAAGTAGTGAGTAAGCAATATGCAGGGAAGATCCTGTACCATCAACCCCGGAAAACCGCATATATGAAGTC : 6087
LSD/090830 : AGCCAATAGGGTCTTTCCAGTTTTTAATCAAGGTAAAGTAGTGAGCAAGCAATATGCAGGTAAGATTCTGTACCATCAACCCCGAAAAACTGCATATATGAAGTC : 6089
FZ99       : AGCCAATAGGGTCTTTCCAGTTTTTAATCAAGGCAAAGTAGTGAGTAAGCAATATGCAGGGAAGATTCTGTACCATCAACCCCGAAAAACTGCATATATGAAGTC : 6088
             AGCCAATAGGGTCTTtCCAGTTTTTAATCAAGG AAAGTAGTGAG AAgCaATATGCAGG AAgAtTCTGTACCATCAACCCCGaAAAACtGCATATATGAAGTC

                     6100         *        6120         *        6140         *        6160         *        6180         *
CH60       : ACCTGTCTATGAGGACTCACCTTATGAGCCTGCAGTTCTTTCCATCAATGACCAGCGACTTGAGGTGAAAATTGAAGACATGGCTAAGAAAGCATCTGACAAGTA : 6193
A66        : ACCTGTCTATGAGGACTCACCTTATGAGCCTGCAGTTCTTTCCATCAATGACCAGCGACTTGAGGTGAAAATTGAAGACATGGCTAAGAAAGCATCTGACAAGTA : 6193
C80        : ACCTGTCTATGAGGACTCACCTTATGAGCCTGCAGTTCTTTCCATCAATGACCAGCGACTTGAGGTGAAAATTGAAGACATGGCTAAGAAAGCATCTGACAAGTA : 6192
MY         : ACCTGTCTATGAGGACTCACCTTATGAGCCTGCAGTTCTTTCCATCAATGACCAGCGACTTGAGGTGAAAATTGAAGACATGGCTAAGAAAGCATCTGACAAGTA : 6193
FC64       : ACCTGTCTATGAGGACTCACCTTATGAGCCTGCAGTTCTTTCCATCAATGACCAGCGACTTGAGGTGAAAATTGAAGACATGGCTAAGAAAGCATCTGACAAGTA : 6193
03D        : ACCTGTCTATGAGGACTCACCCTATGAGCCTGCTGTCCTTTCCATCAATGACCAGCGACTTGGAGTGAAGATTGAAGACATGGCTAAGAAAGCATCTGACAAGTA : 6190
ZJ         : ACCTGTCTATGAGGACTCACCCTATGAGCCTGCTGTCCTTTCTATCAATGACCAGCGACTTGGAGTGAAGATTGAAGACATGGCTAAGAAAGCATCTGACAAGTA : 6190
NA         : ACCTGTCTATGAGGACTCACCCTATGAGCCTGCTGTCCTTTCCATCAATGACCAGCGACTTGGAGTGAAGATTGAAGACATGGCTAAGAAAGCATCTGACAAGTA : 6193
C-XNH      : ACCTGTCTATGAGGACTCACCCTATGAGCCCGCTGTCCTTTCCATCAATGACCAGCGACTTGGAGTGAAGATTGAAGACATGGCTAAGAAAGCATCTGACAAGTA : 6192
LSD/090830 : ACCTGTCTATGAGGACTCACCCTATGAGCCTGCTGTCCTTTCCATTAATGACCAGCGACTTGGAGTGAAGATTGAAGACATGGCTAAGAAAGCATCTGACAAGTA : 6194
FZ99       : ACCTGTCTATGAGGACTCACCCTATGAGCCTGCTGTCCTTTCCATCAATGACCAGCGACTTGGAGTGAAGATTGAAGACATGGCTAAGAAAGCATCTGACAAGTA : 6193
             ACCTGTCTATGAGGACTCACC TATGAGCCtGC GT CTTTCcATcAATGACCAGCGACTTG  GTGAA ATTGAAGACATGGCTAAGAAAGCATCTGACAAGTA

                 6200         *        6220         *        6240         *        6260         *        6280         *        6300
CH60       : TATTGGCAATGTGTTTCAGCCTCCTCCTGAGGCTTTTCAACTTGCAAAGACACATGTTGCCGAAAAACTGTCCAGAGTCTTGGGAAGCCATGAAACTATCTCATA : 6298
A66        : TATTGGCAATGTGTTTCAGCCTCCTCCTGAGGCTTTTCAACTTGCAAAGACACATGTTGCCGAAAAACTGTCCAGAGTCTTGGGAAGCCATGAAACTATCTCATA : 6298
C80        : TATTGGCAATGTGTTTCAGCCTCCTCCTGAGGCTTTTCAACTTGCAAAGACACATGTTGCCGAAAAACTGTCCAGAGTCTTGGGAAGCCATGAAACTATCTCATA : 6297
MY         : TATTGGCAATGTGTTTCAGCCTCCTCCTGAGGCTTTTCAACTTGCAAAGACACATGTTGCCGAAAAACTGTCCAGAGTCTTGGGAAGCCATGAAACTATCTCATA : 6298
FC64       : TATTGGCAATGTGTTTCAGCCTCCTCCTGAGGCTTTTCAACTTGCAAAGACACATGTTGCCGAAAAACTGTCCAGAGTCTTGGGAAGCCATGAAACTATCTCATA : 6298
03D        : TATTGGCAATGTGTTTCAGCCTCCTCCTGAGGCTTTCCAACTTGCAAAGACACATGTTGCCGAAAAGCTGTCTAGAGTATTGGGATGCCATGATACTATCTCATA : 6295
ZJ         : TATTGGCAATGTGTTTCAGCCTCCTCCTGAGGCTTTCCAACTTGCAAAGACACATGTTGCTGAAAAACTGTCCAGAGTCTTGGGATGCCATGATATTATCTCATA : 6295
NA         : TATTGGCAATGTGTTTCAGCCTCCTCCTGAGGCTTTCCAACTTGCAAAGACACATGTTGCTGAAAAACTGTCCAGAGTCTTGGGATGCCATGATATTATCTCATA : 6298
C-XNH      : TATTGGCAATGTGTTTCAGCCTCCTCCTGAGGCTTTCCAACTTGCAAAGACACATGTTGCCGAAAAGCTATCCAGAGTCTTGGGATGCCATGATATTATCTCATA : 6297
LSD/090830 : TATTGGCAATGTGTTTCAGCCTCCTCCTGAGGCTTTCCAACTTGCAAAGACACATGTTGCCGAAAAGCTGTCCAGAGTCTTGGGATGCCATGATACTATATCATA : 6299
FZ99       : TATTGGCAATGTGTTTCAGCCTCCTCCTGAGGCTTTCCAACTTGCAAAGACACATGTTGCCGAAAAGCTGTCCAGAGTCTTGGGATGCCATGATATTATCTCATA : 6298
             TATTGGCAATGTGTTTCAGCCTCCTCCTGAGGCTTT CAACTTGCAAAGACACATGTTGCcGAAAA CTgTCcAGAGTcTTGGGA GCCATGA A TATcTCATA

                     *        6320         *        6340         *        6360         *        6380         *        6400
CH60       : TGAGTCAGCTGTGAGTAGTGATGTTATACCTATGAACTGGGATACTTCACCTGGCATTAAGTATAAGGGGGGAGACCAAACGACAACTGGTGCTGAAGTCCTCATT : 6403
A66        : TGAGTCAGCTGTGAGTAGTGATGTTATACCTATGAACTGGGATACTTCACCTGGCATTAAGTATAAGGGGGGAGACCAAACGACAACTGGTGCTGAAGTCCTCATT : 6403
C80        : TGAGTCAGCTGTGAGTAGTGATGTTATACCTATGAACTGGGATACTTCACCTGGCATTAAGTATAAGGGGGGAGACCAAACGACAACTGGTGCTGAAGTCCTCATT : 6402
MY         : TGAGTCAGCTGTGAGTAGTGATGTTATACCTATGAACTGGGATACTTCACCTGGCATTAAGTATAAGGGGGGAGACCAAACGACAACTGGTGCTGAAGTCCTCATT : 6403
FC64       : TGAGTCAGCTGTGAGTAGTGATGTTATACCTATGAACTGGGATACTTCACCTGGCATTAAGTATAAGGGGGGAGACCAAACGACAACTGGTGCTGAAGTCCTCATT : 6403
03D        : TGAGTCAGCTGTGAGTAGTGATGTTATACCTATGAACTGGGACACTTCACCTGGCATTAAGTATAAGGGGGGAGACTAAACGACAACTGGTGCTGAAGTCCTCATT : 6400
ZJ         : TGAGTCAGCTGTGAGTAGTGATGTTATACCTATGAACTGGGACACTTCGCCTGGCATTAAGTATAAGGGGGGAGACTAAACGGCAACTGGTGCTGAAGTCCTCATT : 6400
NA         : TGAGTCAGCTGTGAGTAGTGATGTTATACCTATGAACTGGGACACTTCGCCTGGCATTAAGTATAAGGGGGGAGACTAAACGGCAATTGGTGCTGAAGTCCTCATT : 6403
C-XNH      : TGAGTCAGCTGTGAGTAGTGATGTTATACCTATGAACTGGGACACTTCGCCTGGCATTAAGTATAAGGGGGGAGACTAAGCGGCAATTGGTGCTGAAGTCCTCATT : 6402
LSD/090830 : TGAGTCAGCTGTGAGTAGTGATGTTATACCTATGAACTGGGACACTTCGCCTGGCATCAAGTATAAGGGGGGAGACCAAACGACAACTGGTGCTGAAGTCCTCGTT : 6404
FZ99       : TGAGTCAGCTGTGAGTAGTGATGTTATACCTATGAACTGGGACACTTCGCCTGGCATTAAGTATAAGGGGGGAGACTAAACGGCAATTGGTGCTGAAGTCCTCATT : 6403
             TGAGTCAGCTGTGAGTAGTGATGTTATACCTATGAACTGGGA ACTTC CCTGGCATtAAGTATAAGGGGGGAGAC AAaCG CAA TGGTGCTGAAGTCCTCaTT

                 *        6420         *        6440         *        6460         *        6480         *        6500         *
CH60       : CAGACAGGATGTCATGGAGCAACTACAGAGTCCATCCACTGTTTTCACTTGTTATCTGAAGGATGAACTCCGAAAGAAGGAAAAAATCAAGGAAGGGAAAACAAG : 6508
A66        : CAGACAGGATGTCATGGAGCAACTACAGAGTCCATCCACTGTTTTCACTTGTTATCTGAAGGATGAACTCCGAAAGAAGGAAAAAATCAAGGAAGGGAAAACAAG : 6508
C80        : CAGACAGGATGTCATGGAGCAACTACAGAGTCCATCCACTGTTTTCACTTGTTATCTGAAGGATGAACTCCGAAAGAAGGAAAAAATCAAGGAAGGGAAAACAAG : 6507
MY         : CAGACAGGATGTCATGGAGCAACTACAGAGTCCATCCACTGTTTTCACTTGTTATCTGAAGGATGAACTCCGAAAGAAGGAAAAAGTCAAGGAAGGGAAAACAAG : 6508
FC64       : CAGACAGGATGTCATGGAGCAACTACAGAGTCCATCCACTGTTTTCACTTGTTATCTGAAGGATGAACTCCGAAAGAAGGAAAAAATCAAGGAAGGGAAAACAAG : 6508
03D        : CAGACAGGATGTTCTGAAGCAACTACAGAGTCCATCCACTGTTTTCACTTGTTATCTGAAGGATGAACTCCGAAAGAAGGAGAAAATCAAGGAAGGGAAAACAAG : 6505
ZJ         : CAGACAGGATGTTCTGGAACAACTACAGAGTCCATCCACTGTTTTCACTTGTTATCTGAAGGATGAACTCCGAAAGAAGGAGAAAATCAAAGAAGGGAAAACAAG : 6505
NA         : CAGACAGGATGTTCTGGAACAACTACAGAGTCCATCCACTGTTTTCACTTGTTATCTGAAGGATGAACTCCGAAAGAAGGAGAAAATCAAAGAAGGGAAAACAAG : 6508
C-XNH      : CAGACAGGATGTTTTGGAACAACTACAGAGTCCATCCACTGTTTTCACTTGTTATCTGAAGGATGAACTCCGAAAGAAGGAGAAAATCAAGGAAGGGAAAACAAG : 6507
LSD/090830 : TAGACAGGATGTTCTGGAGCAATTGCAGAGTCCATCCACTGTTTTCACTTGTTATCTGAAGGATGAACTTCGAAAGAAGGAGAAAATCAAGGAAGGGAAAACAAG : 6509
FZ99       : CAGACAAGATGTTCTGGAACAACTACAGAGTCCATCCACTGTTTTCACTTGTTATCTGAAGGATGAACTCCGAAAGAAGGAGAAAATCAAGGAAGGGAAAACAAG : 6508
             cAGACAgGATGT  TGgA CAAcTaCAGAGTCCATCCACTGTTTTCACTTGTTATCTGAAGGATGAACTcCGAAAGAAGGA AAAaTCAAgGAAGGGAAAACAAG

                     6520         *        6540         *        6560         *        6580         *        6600         *
CH60       : AGGCATTGAGGCTTGTAATTTTGATCACACCGTGGCTTATAGAATGGTGATGGGTGATATTTTTTCTAACATCTATGATGATTCTTTTATTATGTCTGGCTGTGC : 6613
A66        : AGGCATTGAGGCTTGTAATTTTGATCACACCGTGGCTTATAGAATGGTGATGGGTGATATTTTTTCTAACATCTATGATGATTCTTTTATTATGTCTGGCTGTGC : 6613
C80        : AGGCATTGAGGCTTGTAATTTTGATCACACCGTGGCTTATAGAATGGTGATGGGTGATATTTTTTCTAACATCTATGATGATTCTTTTATTATGTCTGGCTGTGC : 6612
MY         : AGGCATTGAGGCTTGTAATTTTGATCACACCGTGGCTTATAGAATGGTGATGGGTGATATTTTTTCTAACATCTATGATGATTCTTTTATTATGTCTGGCTGTGC : 6613
FC64       : AGGCATTGAGGCTTGTAATTTTGATCACACCGTGGCTTATAGAATGGTGATGGGTGATATTTTTTCTAACATCTATGATGATTCTTTTATTATGTCTGGCTGTGC : 6613
03D        : AGGCATTGAGGCTTGTAACTTTGATCACACTGTGGCTTATAGAATGGTGATGGGTGACATTTTTTCTAATATCTATGATGATTCTTTTATTATGTCTGGCTGTGC : 6610
ZJ         : AGGCATTGAGGCTTGTAATTTTGATCACACCGTGGCTTATAGAATGGTGATGGGTGACATTTTTTCTAACATCTATGATGATTCTTTTATTATGTCTGGCTGTGC : 6610
NA         : AGGCATTGAGGCTTGTAATTTTGATCACACCGTGGCTTATAGAATGGTGATGGGTGACATTTTTTCTAACATCTATGATGATTCTTTTATTATGTCTGGCTGTGC : 6613
C-XNH      : AGGTATTGAGGCTTGTAATTTTGATCACACCGTGGCTTATAGAATGGTGATGGGTGACATTTTTTCCAACATCTATGATGATTCTTTTATTGTGTCTGGCTGTGC : 6612
LSD/090830 : AGGTATTGAGGCTTGTAATTTTGATCACACTGTGGCTTATAGAATGGTGATGGGTGACATTTTTTCTAACATTTATGATGATTCTTTTATTATGTCTGGCTGTGC : 6614
FZ99       : AGGCATTGAGGCTTGTAATTTTGATCACACCGTGGCTTATAGAATGGTGATGGGTGACATTTTTTCTAACATCTATGATGATTCTTTTATTATGTCTGGCTGTGC : 6613
             AGGcATTGAGGCTTGTAAtTTTGATCACACcGTGGCTTATAGAATGGTGATGGGTGA ATTTTTTCtAAcATcTATGATGATTCTTTTATTaTGTCTGGCTGTGC

                 6620         *        6640         *        6660         *        6680         *        6700         *        6720
CH60       : TGTTGGAATTAATCCTTTTTGTGAGTGGGACAATCTGTTGGCAAACCTCCAACCTTATAATTTGTGTCTTGACTTTTCTGGATTCGATGGCTCTCTGAGTGCCCA : 6718
A66        : TGTTGGAATTAATCCTTTTTGTGAGTGGGACAATCTGTTGGCAAACCTCCAACCTTATAATTTGTGTCTTGACTTTTCTGGATTCGATGGCTCTCTGAGTGCCCA : 6718
C80        : TGTTGGAATTAATCCTTTTTGTGAGTGGGACAATCTGTTGGCAAACCTCCAACCTTATAATTTGTGTCTTGACTTTTCTGGATTCGATGGCTCTCTGAGTGCCCA : 6717
MY         : TGTTGGAATTAATCCTTTTTGTGAGTGGGACAATCTGTTGGCAAACCTCCAACCTTATAATTTGTGTCTTGACTTTTCTGGATTTGATGGCTCTCTGAGTGCCCA : 6718
FC64       : TGTTGGAATTAATCCTTTTTGTGAGTGGGACAATCTGTTGGCAAACCTCCAACCTTATAATTTGTGTCTTGACTTTTCTGGATTCGATGGCTCTCTGAGTGCCCA : 6718
03D        : TGTTGGAATTAATCCTTTTTGTGAGTGGGACAATTTGTTGGCAAACCTCCAACCTTATAATTTGTGTCTTGACTTTTCTGGATTCGATGGCTCTCTGAGTGCCCA : 6715
ZJ         : TGTTGGAATTAATCCTTTTTGTGAGTGGGACAATCTGTTGGCAAACCTCCAACCTTATAATTTGTGTCTTGATTTTTCTGGATTCGATGGCTCTCTGAGTGCCCA : 6715
NA         : TGTTGGAATTAATCCTTCTTGTGAGTGGGACAATCTGTTGGCAAACCTCCAACCTTATAATTTGTGTCTTGATTTTTCTGGATTCGATGGCTCTCTGAGTGCCCA : 6718
C-XNH      : TGTTGGAATTAATCCTTTTTGTGAGTGGGACAATCTGTTGGCAAACCTCCAACCTTATAATCTGTGTCTTGACTTTTCTGGATTCGATGGCTCTCTGAGTGCCCA : 6717
LSD/090830 : TGTTGGAATTAATCCTTTTTGTGAGTGGGACAATCTGTTGGCAAATCTCCAACCTTATAACTTGTGTCTTGACTTTTCTGGATTCGATGGCTCTCTGAGTGTCCA : 6719
FZ99       : TGTTGGAATTAATCCTTTTTGTGAGTGGGACAATCTGTTGGCAAACCTCCAACCTTATAATTTGTGTCTTGACTTTTCTGGATTCGATGGCTCTCTGAGTGCCCA : 6718
             TGTTGGAATTAATCCTTtTTGTGAGTGGGACAATcTGTTGGCAAAcCTCCAACCTTATAAttTGTGTCTTGAcTTTTCTGGATTcGATGGCTCTCTGAGTGcCCA

                     *        6740         *        6760         *        6780         *        6800         *        6820
CH60       : AATTCTTGAAGAAGCAGTGGATGTCTTGTCTTATTTCCACAATGACCCAGCCTTAGTGAAAAGAATTCATGCACCAACTATCTATTCAACTCATTATGTAACTGA : 6823
A66        : AATTCTTGAAGAAGCAGTGGATGTCTTGTCTTATTTCCACAATGACCCAGCCTTAGTGAAAAGAATTCATGCACCAACTATCTATTCAACTCATTATGTAACTGA : 6823
C80        : AATTCTTGAAGAAGCAGTGGATGTCTTGTCTTATTTCCACAATGACCCAGCCTTAGTGAAAAGAATTCATGCACCAACTATCTATTCAACTCATTATGTAACTGA : 6822
MY         : AATTCTTGAAGAAGCAGTGGATGTCTTGTCTTATTTCCACAGTGACCCAGCCTTAGTGAAAAGAATTCATGCACCAACTATCTATTCAACTCATTATGTAACTGA : 6823
FC64       : AATTCTTGAAGAAGCAGTGGATGTCTTGTCTTATTTCCACAATGACCCAGCCTTAGTGAAAAGAATTCATGCACCAACTATCTATTCAACTCATTATGTAACTGA : 6823
03D        : AATTCTTGAAGAAGCAGTTGATGTATTGTCTTATTTTCACAATGACCCAGCCTTAGTGAAAAGAATTCATGCACCAACTATCTATTCAACTCATTATGTAACTGA : 6820
ZJ         : AATTCTTGAAGAAGCAGTTGATGTATTGTCTTATTTTCACAATGACCCAGCCTTAGTGAAAAGAATTCATGCACCAACTATTTATTCAACTCATTATGTAACTGA : 6820
NA         : AATTCTTGAAGAAGCAGTTGATGTATTGTCTTATTTTCACAACGACCCAGCCTTAGTGAAAAGAATTCATGCACCAACTATTTATTCAACTCATTATGTAACTGA : 6823
C-XNH      : AATCCTTGAAGAAGCAGTTGATATATTGTCTTATTTCCACAATGACCCAGCCTTAGTGAAAAGAATTCATGCACCAACTATTTATTCAACTCATTATGTAACTGA : 6822
LSD/090830 : AATTCTTGAAGAAGCAGTGGATGTATTGTCATATTTCCACAATGACCCAGCCTTAGTGAAAAGAATTCATGCACCAACTATCTATTCAACTCATTATGTAACTGA : 6824
FZ99       : AATTCTTGAAGAAGCAGTTGATGTATTGTCTTATTTCCACAATGACCCAGCCTTAGTGAAAAGAATTCATGCACCAACTATTTATTCAACTCATTATGTAACTGA : 6823
```

```
            AATtCTTGAAGAAGCAGT GATgT TTGTCtTATTT CACAatGACCCAGCCTTAGTGAAAAGAATTCATGCACCAACTAT TATTCAACTCATTATGTAACTGA

                  *       6840          *         6860          *         6880          *         6900          *         6920          *
CH60       : TGAGATATGGCAGGTAGAAGGAGGGATGTGTTCAGGATCCCCATGTACTACCGTGGTCAATTCTATTGTGAATCAGCTTGCGTGCTACACCATTTTAGCTGTATT : 6928
A66        : TGAGATATGGCAGGTAGAAGGAGGGATGTGTTCAGGATCCCCATGTACTACCGTGGTCAATTCTATTGTGAATCAGCTTGCGTGCTACACCATTTTAGCTGTATT : 6928
C80        : TGAGATATGGCAGGTAGAAGGAGGGATGTGTTCAGGATCCCCATGTACTACCGTGGTCAATTCTATTGTGAATCAGCTTGCGTGCTACACCATTTTAGCTGTATT : 6927
MY         : TGAGATATGGCAGGTAGAAGGAGGGATGTGTTCAGGATCCCCATGTACTACCGTGGTCAATTCTATTGTGAATCAGCTTGCGTGCTACACCATTTTAGCTGTATT : 6928
FC64       : TGAGATATGGCAGGTAGAAGGAGGGATGTGTTCAGGATCCCCATGTACTACCGTGGTCAATTCTATTGTGAATCAGCTTGCGTGCTACACCATTTTAGCTGTATT : 6928
03D        : TGAGATATGGCAGGTAGAAGGAGGGATGTGTTCAGGATCTCCATGTACCACTGTGGTCAATTCTATTGTGAATCAGCTTGCATGTTACACCATTTTAGCTGTCTT : 6925
ZJ         : TGAGATATGGCAGGTAGAAGGAGGGATGTGTTCAGGATCTCCATGTACCACTGTGGTCAATTCTATTGTAAATCAGCTTGCTTGTTATACCATCTTAGCTGTCTT : 6925
NA         : TGAGATATGGCAGGTAGAAGGAGGGATGTGTTCAGGATCTCCATGTACCACTGTGGTCAATTCTATTGTAAATCAGCTTGCTTGTTATACCATCTTAGCTGTCTT : 6928
C-XNH      : TGAGATATGGCAGGTAGAAGGAGGGATGTGTTCAGGATCTCCATGTACCACTGTGATTAATTCTATTGTAAACCAGCTTGCTTGTTACACCATTTTAGCTGTCTT : 6927
LSD/090830 : TGAGATATGGCAGGTAGAAGGAGGGATGTGTTCAGGATCCCCATGTACCACTGTGGTCAATTCTATTGTAAATCAGCTTGCATGTTACACTATTTTAGCTGTCTT : 6929
FZ99       : TGAGATATGGCAGGTAGAAGGAGGGATGTGTTCAGGATCTCCATGTACCACTGTGGTCAATTCTATTGTAAATCAGCTTGCTTGTTACACCATTTTAGCTGTCTT : 6928
             TGAGATATGGCAGGTAGAAGGAGGGATGTGTTCAGGATC CCATGTAC AC GTGgTcAATTCTATTGT AAtCAGCTTGC TG TAcACcATtTTAGCTGT TT

                    6940          *         6960          *         6980          *         7000          *         7020          *
CH60       : GGGCTATGACATCAACCAATGTTATGTCGTTAGTTATGGGGATGACTGTGTTCTGTCAGTGCCAGAGGTGCGCGATATCTCAAAACTATCGCACTATTTCAAGCT : 7033
A66        : GGGCTATGACATCAACCAATGTTATGTCGTTAGTTATGGGGATGACTGTGTTCTGTCAGTGCCAGAGGTGCGCGACATCTCAAAACTATCGCACTATTTCAAGCT : 7033
C80        : GGGCTATGACATCAACCAATGTTATGTCGTTAGTTATGGGGATGACTGTGTTCTGTCAGTGCCAGAGGTGCGCGATATCTCAAAACTATCGCACTATTTCAAGCT : 7032
MY         : GGGCTATGACATCAACCAATGTTATGTCGTTAGTTATGGGGATGACTGTGTTCTGTCAGTGCCAGAGGTGCGCGATATCTCAAAACTATCGCACTATTTCAAGCT : 7033
FC64       : GGGCTATGACATCAACCAATGTTATGTCGTTAGTTATGGGGATGACTGTGTTCTGTCAGTGCCAGAGGTGCGCGATATCTCAAAACTATCGCACTATTTCAAGCT : 7033
03D        : GGGCTATGACATCAACCAATGTTATGTCGTTAGTTATGGGGATGATTGTGTTCTGTCAGTGCCAGAGGTGCGCGATATCTCAAAACTATCGCACTATTTTAAGCT : 7030
ZJ         : GGGCTATGACATCAACCAATGTTATGTCGTTAGTTATGGGGATGACTGTGTCCTGTCAGTGCCAGAGGTGCGCGATATTTCAAAACTATCGCACTATTTTAAGCT : 7030
NA         : GGGCTATGACATCAACCAATGTTATGTCGTTAGTTATGGGGATGACTGTGTCCTGTCAGTGCCAGAGGTGCGCGATATTTCAAAACTATCGCACTATTTTAAGCT : 7033
C-XNH      : GGGCTATGACATCAACCAATGTTATGTCGTTAGTTATGGGGATGACTGTGTCCTGTCAGTGCCAGAGGTGCGCGATATTTCAAAACTATCGCACTATTTTAAGCT : 7032
LSD/090830 : GGGCTATGACATCAACCAATGTTATGTCGTTAGTTATGGGGATGACTGTGTTCTGTCAGTGCCAGAGGTGCGCGATATCTCAAAACTATCGCATTATTTCAAGCT : 7034
FZ99       : GGGCTATGACATTAACCAATGTTATGTCGTTAGTTATGGGGATGACTGTGTCCTGTCAGTGCCAGAGGTGCGCGATATCTCAAAACTATCGCACTATTTCAAGCT : 7033
             GGGCTATGACATcAACCAATGTTATGTCGTTAGTTATGGGGATGAcTGTGT CTGTCAGTGCCAGAGGTGCGCGAtAT TCAAAACTATCGCAcTATTT AAGCT

               7040          *         7060          *         7080          *         7100          *         7120          *         7140
CH60       : TTTCTTTGGTATGACAGCAACAGCTAGTGACAAAGAAAGTGACATAACTTGGCTAGCCCCTATGGAGATAGAATTTCTGAAGAGAACTCCTGCCTTCCTCCCAGG : 7138
A66        : TTTCTTTGGTATGACAGCAACAGCTAGTGACAAAGAAAGTGACATAACTTGGCTAGCCCCTATGGAGATAGAATTTCTGAAGAGAACTCCTGCCTTCCTCCCAGG : 7138
C80        : TTTCTTTGGTATGACAGCAACAGCTAGTGACAAAGAAAGTGACATAACTTGGCTAGCCCCTATGGAGATAGAATTTCTGAAGAGAACTCCTGCCTTCCTCCCAGG : 7137
MY         : TTTCTTTGGTATGACAGCAACAGCTAGTGACAAAGAAAGTGACATAACTTGGCTAGCCCCTATGGAGATAGAATTTCTGAAGAGAACTCCTGCCTTCCTCCCAGG : 7138
FC64       : TTTCTTTGGTATGACAGCAACAGCTAGTGACAAAGAAAGTGACATAACTTGGCTAGCCCCTATGGAGATAGAATTTCTGAAGAGAACTCCTGCCTTCCTCCCAGG : 7138
03D        : TTTCTTTGGTATGACAGCAACAGCTAGTGACAAAGAAAGTGACATAACTTGGCTAGCCCCTATGGAGATAGAATTTCTGAAGAGAACTCCTGCCTTCCTCCCAGG : 7135
ZJ         : TTTCTTTGGTATGACAGCAACAGCTAGTGACAAAGAAAGTGACATAACTTGGCTAGCCCCTATGGAGATAGAATTTTTGAAGAGAACTCCTGCCTTCCTCCCAGG : 7135
NA         : TTTCTTTGGTATGACAGCAACAGCTAGTGACAAAGAAAGTGACATAACTTGGCTAGCCCCTATGGAGATAGAATTTTTGAAGAGGACTCCTGCCTTCCTCCCAGG : 7138
C-XNH      : TTTCTTTGGTATGACAGCAACAGCTAGTGACAAAGAAAGTGACATAACTTGGCTAGCCCCTATGGAGATAGAATTTTTGAAGAGAACTCCTGCCTTCCTCCCAGG : 7137
LSD/090830 : CTTCTTTGGTATGACAGCAACAGCTAGTGACAAAGAAAGTGACATAACTTGGCTAGCCCCTATGGAGATAGAATTTCTGAAGAGAACTCCTGCCTTCCTCCCAGG : 7139
FZ99       : TTTCTTTGGTATGACAGCAACAGCTAGTGACAAAGAAAGTGACATAACTTGGCTAGCCCCTATGGAGATAGAATTTTTGAAGAGAACTCCTGCCTTCCTCCCAGG : 7138
             tTTCTTTGGTATGACAGCAACAGCTAGTGACAAAGAAAGTGACATAACTTGGCTAGCCCCTATGGAGATAGAATTT TGAAGAGaACTCCTGCCTTCCTCCCAGG

                       *         7160          *         7180          *         7200          *         7220          *         7240
CH60       : GACTCGCAAAATCATTGGTGTCCTTGATAAGGAAGTTTTGGAAGGGAAGATACAGTGGTGCAAGGGACCAGAAGCATTCAAACAGCAACTTGACTCTTTCTTGCT : 7243
A66        : GACTCGCAAAATCATTGGTGTCCTTGATAAGGAAGTTTTGGAAGGGAAGATACAGTGGTGCAAGGGACCAGAAGCATTCAAACAGCAACTTGACTCTTTCTTGCT : 7243
C80        : GACTCGCAAAATCATTGGTGTCCTTGATAAGGAAGTTTTGGAAGGGAAGATACAGTGGTGCAAGGGACCAGAAGCATTCAAACAGCAACTTGACTCTTTCTTGCT : 7242
MY         : GACTCGCAAAATCATTGGTGTCCTTGATAAGGAAGTTTTGGAAGGGAAGATACAGTGGTGCAAGGGACCAGAAGCATTCAAACAGCAACTTGACTCTTTCTTGCT : 7243
FC64       : GACTCGCAAAATCATTGGTGTCCTTGATAAGGAAGTTTTGGAAGGGAAGATACAGTGGTGCAAGGGACCAGAAGCATTCAAACAGCAACTTGACTCTTTCTTGCT : 7243
03D        : GACCCGCAAAATTATTGGTGTCCTTGACAAAGAAGTCTTGGAAGGGAAGATACAGTGGTGCAAGGGACCAGAAGCATTCAAACAGCAACTTGACTCTTTCTTGCT : 7240
ZJ         : GACCCGCAAAATTATTGGTGTCCTTGACAAGGAAGTCTTGGAAGGGAAGATACAGTGGTGTAAGGGACCAGAAGCATTCAAACAGCAACTTGACTCTTTCTTGCT : 7240
NA         : GACCCGCAAAATTATTGGTGTTCTTGACAAGGAAGTCTTGGAAGGGAAGATACAGTGGTGTAAGGGACCAGAAGCATTCAAACAGCAACTTGACTCTTTCTTGCT : 7243
C-XNH      : GACCCGCAAAATTATTGGTGTCCTTGACAAGGAAGTCTTGGAAGGGAAGATACAGTGGTGCAAGGGACCAGAAGCATTCAAACAGCAACTTGACTCTTTCTTGCT : 7242
LSD/090830 : GACCCGCAAAATTATTGGTGTTCTTGACAAGGAAGTTCTGGAAGGGAAGATACAGTGGTGCAAGGGACCAGAAGCATTCAAACAGCAACTTGACTCTTTCTTGCT : 7244
FZ99       : GACCCGCAAAATTATTGGTGTCCTTGACAAGGAAGTCTTGGAAGGGAAGATACAGTGGTGCAAGGGACCAGAAGCATTCAAACAGCAACTTGACTCTTTCTTGCT : 7243
             GAC CGCAAAAT ATTGGTGTcCTTGA AAgGAAGT tTGGAAGGGAAGATACAGTGGTGcAAGGGACCAGAAGCATTCAAACAGCAACTTGACTCTTTCTTGCT

                  *       7260          *         7280          *         7300          *         7320          *         7340          *
CH60       : GGAAGCAGCTCTGCATGGAGAAGAATACTACCATGAGGTGACATCTAAGCTTAAGGCCCGGTGCCCCGTTTTTGACATTCAACCTTGGGGTGTCGCGAAACTCAG : 7348
A66        : GGAAGCAGCTCTGCACGGAGAAGAATACTACCATGAGGTGACATCTAAGCTTAAGGCCCGGTGCCCCGTTTTTGACATTCAACCTTGGGGTGTCGCGAAACTCAG : 7348
C80        : GGAAGCAGCTCTGCATGGAGAAGAATACTACCATGAGGTGACATCTAAGCTTAAGGCCCGGTGCCCCGTTTTTGACATTCAACCTTGGGGTGTCGCGAAACTCAG : 7347
MY         : GGAAGCAGCTCTGCATGGAGAAGAATACTACCATGAGGTGACATCTAAGCTTAAGGCCCGGTGCCCCGTTTTTGACATTCAACCTTGGGGTGTCGCGAAACTCAG : 7348
FC64       : GGAAGCAGCTCTGCATGGAGAAGAATACTACCATGAGGTGACATCTAAGCTTAAGGCCCGGTGCCCCGTTTTTGACATTCAACCTTGGGGTGTCGCGAAACTCAG : 7348
03D        : GGAAGCAGCTCTGCATGGAGAAGAATACTACCATGAGGTGACATCTAAGCTTAAGGCCCGGTGCCCCGTTCTTGACATTCAACCTTGGGGTGTCGCGAAACTCAG : 7345
ZJ         : GGAAGCAGCTCTGCATGGGGAAGAATACTACCATGAGGTGACATCTAAGCTTAAGGCCCGGTGCCCCGTTCTTGACATTCAACCTTGGGGTGTCGCGAAACTCAG : 7345
NA         : GGAAGCAGCTCTGCATGGGGAAGAATACTACCATGAGGTGACATCTAAGCTTAAGGCCCGGTGCCCCGTTCTTGACATTCAACCTTGGGGTGTCGCGAAACTCAG : 7348
C-XNH      : GGAAGCAGCTCTGCATGGGGAAGAATACTACCATGAGGTGACATCTAAGCTTAAAGCCCGGTGCCCCGTTCTTGACATTCAACCTTGGGGTGTTGCAAAACTCAG : 7347
LSD/090830 : GGAAGCAGCTCTACATGGAGAAGAATACTACCATGAGGTGACATCTAAGCTTAAGGCCCGGTGCCCCGTTCTTGACATTCAACCTTGGGGTGTCGCAAAACTCAG : 7349
FZ99       : GGAAGCAGCTCTGCATGGGGAAGAATACTACCATGAGGTGACATCTAAGCTTAAGACCCGGTGCCCCGTTCTTGACATTCAACCTTGGGGTGTCGCGAAACTCAG : 7348
             GGAAGCAGCTCTgCAtGG GAAGAATACTACCATGAGGTGACATCTAAGCTTAAggCCCGGTGCCCCGTT TTGACATTCAACCTTGGGGTGTcGCgAAACTCAG

                    7360          *         7380          *         7400          *         7420          *         7440          *
CH60       : AGCATACACAGCTTGCATGATGATCTGAACTGTTGGTCCGCAGGTACCATAAACCTGTT-TTT-CCTGGGTTTTAGTGTTGTGGGATACCCAGGAGTACACGTAA : 7451
A66        : AGCATACACAGCTTGCATGATGATCTGAACTGTTGGTCCGCAGGTACCATAAACCTGTT-TTT-CCTGGGTTTTAGTGTTGTGGGATACCCAGGAGTACACGTAA : 7451
C80        : AGCATACACAGCTTGCATGATGATCTGAACTGTTGGTCCGCAGGTACCATAAACCTGTT-TTT-CCTGGGTTTTAGTGTTGTGGGATACCCAGGAGTACACGTAA : 7450
MY         : AGCATACACAGCTTGCATGATGATCTGAACTGTTGGTCCGCAGGTACCATAAACCTGTT-TTT-CCTGGGTTTTAGTGTTGTGGGATACCCAGGAGTACACGTAA : 7451
FC64       : AGCATACACAGCTTGCATGATGATCTGAACTGTTGGTCCGCAGGTACCATAAACCTGTTCTTT-CCTGGGTTTTAGTGTTGTGGGATACCCAGGAGTACACATAA : 7452
03D        : AGCATATACAGCTTGCATGATGATCTGAACTTTTGGTCTGCAGGTACCATAAACCTGTTTTT--CCTGGGTTTTAGTGTTGTGGGATACCCAGGAGTACACGTAA : 7448
ZJ         : AGCATATACAGCTTGTATGATGATCTGAACTGTTGGTCTGCAGGTACCATAAACCTGTTTTT--CCTGGGTTTTAGTGTTGTGGGATACCCAGGAGTACACGTAA : 7448
NA         : AGCATATACAGCTTGTATGATGATCTGAATTGTTGGTCTGCAGGTACCATAAACCTGTTTTT--CCTGGGTTTTAGTGTTGTGGGATACCCAGGAGTACACGTAA : 7451
C-XNH      : AGCATATACAGCTTGCATGATGATCTGAACTGTTGGTCTGCAGGTACCATAAACCTGTTTTT--CCTGGGTTTTAGTGTTGTGGGATACCCAGGAGTACACGTAA : 7450
LSD/090830 : AGCATATACAGCTTGCATGATGATTTGAACTGTTGGTTTGCAGGTACCACAAACCTGTTTTTTTCCTGGGTTTTAGTGTTGTGGGATACCCAGGAGTACACGTAA : 7454
FZ99       : AGCATATACAGCTTGCATGATGATCTGAACTGTTGGTCTGCAGGTACCATAAACCTGTTTTT--CCTGGGTTTTAGTGTTGTGGGATACCCAGGAGTACACGTAA : 7451
             AGCATA ACAGCTTGcATGATGATcTGAAcTgTTGGTc GCAGGTACCAtAAACCTGTT TT  CCTGGGTTTTAGTGTTGTGGGATACCCAGGAGTACACgTAA

               7460          *         7480          *         7500          *         7520          *         7540          *         7560
CH60       : AACACCCACTGGCTTTGGAGCTGTGCCACATGACACCTCCCAGCTTGACAGTGCTGTCAGAAACTGTTGTATAGGAATAGGCTAAAACTAATCAATTTACCCCCA : 7556
A66        : AACACCCACTGGCTTTGGAGCTGTGCCACATGACACCTCCCAGCTTGACAGTGCTGTCAGAAACTGTTGTATAGGAATAGGCTAAAACTAATCAATTTACCCCCA : 7556
C80        : AACACCCACTGGCTTTGGAGCTGTGCCACATGACACCTCCCAGCTTGACAGTGCTGTCAGAAACTGTTGTATAGGAATAGGCTAAAACTAATCAATTTACCCCCA : 7555
MY         : AACACCCACTGGCTTTGGAGCTGTGCCACATGACACCTCCCAGCTTGACAGTGCTGTCAGAAACTGTTGTATAGGAATAGGCTAAAACTAATCAATTTACCCCCA : 7556
FC64       : AACACCCACTGGCTTTGGAGCTGTGCCACATGACACCTCCCAGCTTGACAGTGCTGTCAGAAACTGTTGTATAGGAATAGGCTAAAACTAATCAATTTACCCCCA : 7557
03D        : AACACCCACTGGCTTTGGAGCTGTGCCACATGACACCTCCCAGCTTGACAGTGCTGTCAGAAACTGTTATATAGGAATAGGCTAAAACTAATCAATTTACCCCCA : 7553
ZJ         : AACACCCACTGGCTTTGGAGCTGTGCCACATGACGCCTCCCAGCTTGACAGTGCTGTCAGAAACTGTTATTTAGGAATAGGCTAAAACTAATCAATTTACCCCCA : 7553
NA         : AACACCCACTGGCTTTGGAGCTGTGCCACATGACGCCTCCCAGCTTGACAGTGCTGTTAGAAACTGTTATTTAGGAATAGGCTAAAACTAATCAATTTACCCCCA : 7556
C-XNH      : AACACCCACTGGCTTTGGAGCTGTGCCACAAGACACCTCCCGGCTTGACAGTGCTGTCAGAAACTGTTATTTAGGAATAGGCTAAAACTAATCAATTTACCCCCA : 7555
LSD/090830 : AACACCCACTGGCTTTGGAGCTGTGCCATATGACACCTCCCAGCTTGACAGTGCTGTCAGAAACTGTTATATAGGAATAGGCTAAAACTAATCAATTTACCCCCA : 7559
FZ99       : AACACCCACTGGCTTTGGAGCTGTGCCACATGACACCTCCCAGCTTGACAGTGCTGTCAGAAACTGTTATTTAGGAATAGGCTAAAACTAATCAATTTACCCCCA : 7556
             AACACCCACTGGCTTTGGAGCTGTGCCAcAtGACaCCTCCCaGCTTGACAGTGCTGTcAGAAACTGTT T TAGGAATAGGCTAAAACTAATCAATTTACCCCCA

                       *         7580          *         7600          *         7620          *         7640          *         7660
```

```
CH60       : TGATTGTTTAGGTATTAGTTGTTAGTATTTAGTATAAGACATTTGGTGATATAAAGACCTCACACAATCTCCACTTTCCGTGAGGACCCTAAGCCAAATGACTTC : 7661
A66        : TGATTGTTTAGGTATTAGTTGTTAGTATTTAGTATAAGACATTTGGTGATATAAAGACCTCACACAATCTCCACTTTCCGTGAGGACCCTAAGCCAAATGACTTC : 7661
C80        : TGATTGTTTAGGTATTAGTTGTTAGTATTTAGTATAAGACATTTGGTGATATAAAGACCTCACACAATCTCCACTTTCCGTGAGGACCCTAAGCCAAATGACTTC : 7660
MY         : TGATTGTTTAGGTATTAGTTGTTAGTATTTAGTATAAGACATTTGGTGATATAAAGACCTCACACAATCTCCACTTTCCGTGAGGACCCTAAGCCAAATGACTTC : 7661
FC64       : TGATTGTTTAGGTATTAGTTGTTAGTATTTAGTATAAGACATTTGGTGATATAAAGACCTCACACAATCTCCACTTTCCGTGAGGACCCTAAGCCAAATGACTTC : 7662
03D        : TGATTGTTTAGGTATTAGTTGTTAGTATTTAGTATAAGACATTTGGTGATATAAAGACCTCACACAATCTCCACTTTCCGTGAGGACCCTAAGCCAAATGACTTC : 7658
ZJ         : TGATTGTTTAGGTATTAGTTGTTAGTATTTAGTATAAGACATTTGGTGATATAAAGACCTCACACAATCTCCACTTTCCGTGAGGACCCTAAGCCAAATGACTTC : 7658
NA         : TGATTGTTTAGGTATTAGTTGTTAGTATTTAGTATAAGACATTTGGTGATATAAAGACCTCACACAATCTCCACTTTCCGTGAGGACCCTAAGCCAAATGACTTC : 7661
C-XNH      : TGATTGTTTAGGTGTTAGTTGTTAGTATTTAGTTTAAGACATTTGGTGATATAAAGACCTCACACAATCTCCACTTTCCGTGAGGACCCTAAGCCAAATGACTTC : 7660
LSD/090830 : TGATTGTTTAGGTATTAGTTGTTAGTATTTAGTATAAGACATTTGGTGATATAAAGACCTCACACAATCTCCACTTCCCGTGAGGACCCTAAGCCAAATGACTTC : 7664
FZ99       : TGATTGTTTAGGTGTTAGTTGTTAGTATTTAGTATAAGACATTTGGTGATATAAAGACCTCACACAATCTCCACTTTCCGTGAGGACCCTAAGCCAAATGACTTC : 7661
             TGATTGTTTAGGTaTTAGTTGTTAGTATTTAGTaTAAGACATTTGGTGATATAAAGACCTCACACAATCTCCACTTtCCGTGAGGACCCTAAGCCAAATGACTTC

                 *      7680         *      7700         *
CH60       : TTTTTACTTTACTATTCCCTACCCTACCT--------------------- : 7690
A66        : TTTTTGCTTTACTATTCCCTACCCTACCTAAAAAAAAAAAAA-------- : 7703
C80        : TTTTTACTTTACTATTCCCTACCCTACCTAAAAAAAAAAAAAAAAAA--- : 7707
MY         : TTTTTACTTTACTATTCCCTACCCTACCTAA------------------- : 7692
FC64       : TTTTTACTTTACTATTCCCTACCCTACCTAAAAA---------------- : 7696
03D        : TTTTTACTTTACTATTCCCTACCTTACCT--------------------- : 7687
ZJ         : TTTTTACTTTACTATTCCCTACCCTACCTAAAAAAAAAAAAAAAAAAA-- : 7706
NA         : TTTTTACTTTACTATTCCCTACCTTACCTAA------------------- : 7692
C-XNH      : TTTTTACTTTACTATTCCCTACCCTACCT--------------------- : 7689
LSD/090830 : TTTTTACTTTACTATTCCCTACCCTACCTAAAAAAAAAAAA--------- : 7705
FZ99       : TTTTTACTTTACTATTCCCTACCCTACCCAAAAAAAAAAACCTATAGTGG : 7711
             TTTTTaCTTTACTATTCCCTACCcTACCt
```

Supplementary data.2 Protein sequence alignment between virulent and attenuated strains

```
                              *        20         *        40         *        60         *        80         *       100
CH60       : MDTLTKNIEDATVNIIGSCAEKVEEAISGLGAVESVASTNSAIATANATTTQTILDPTEGSTDDFYSCSYEVGAQGDNISRLVHLVTGQWVPNDDYYACLRWLAT :  105
A66        : MDTLTKNIEDATVNIIGSCAEKVEEAISGLGAVESVASTNSAIASANATTTQTILDPTEGSTDDFYSCSYEVGAQGDNISRLVHLVTGQWVPNDDYYACLRWLAT :  105
C80        : MDTLTKNIENATVNIIGSCAEKVEEAISGLGAVESVASTNSAIATANATTTQTILDPTEGSTDDFYSCSYEVGAQGDNISRLVHLVTGQWVPNDDYYACLRWLAT :  105
MY         : MDTLTKNIEDATVNIIGSCAEKVEEAISGLGAVESVASTNSAIATANATTTQTILDPTEGSTDDFYSCSYEVGAQGDNISRLVHLVTGQWVPNDDYYACLRWLAT :  105
FC64       : MDTLTKNIEDATVNIIGSCAEKVEEAISGLGAVESVASTNSAIATANATTTQTILDPTEGSTDDFYSCSYEVGAQGDNISRLVHLVTGQWVPNDDYYACLRWLAT :  105
03D        : MDTLTKNIEDATVNIIGSCAEKVEEAISGLGAVESVASTNSAIATANATTTQTIPDPTEGSTDDFYSCSYEVGAQGDNISRLVHLVTGQWVPNDDYYACLRWLAT :  105
ZJ         : MDTLTKNIEDATVNIIGSCAEKVEEAISGLGAVESVASTNSAIATANATTTQTIPDPTEGSTDDFYSCSYEVGAQGDNISRLVHLVTGQWIPNDDYYACLRWLAT :  105
NA         : MDTLTKNIEDATVNIIGSCAEKVEEAISGLGAVESVASTNSAIATANATTTQTIPDPTEGSTDDFYSCSYEVGAQGDNISRLVHLVTGQWIPNDDYYACLRWLAT :  105
C-XNH      : MDTLTKNIEDATVNIIGSCAEKVEEAISGLGAVESVASTNSAIATANATTTQTIPDPTEGSTDDFYSCSYEVGAQGDNISRLVHLVTGQWIPNDDYYACLRWLAT :  105
LSD/090830 : MDTLTKNIEDATVNIIGSCAEKVEEAISGLGAVESVASTNSAIATANATTTQTIPDPTEGSTDDFYSCSYEVGAQGDNISRLVHLVTGQWVPNDDYYVCLRWLAT :  105
FZ99       : MDTLTKNIEDATVNIIGSCAEKVEEAISGLGAVESVASTNSAIATTNATTTQTIPDPTEGSTDDFYSCSYEVGAQGDNISRLVHLVTGQWIPNDDYYACLRWLAT :  105
             MDTLTKNIE1ATVNIIGSCAEKVEEAISGLGAVESVASTNSAIA3aNATTTQTI DPTEGSTDDFYSCSYEVGAQGDNISRLVHLVTGQW6PNDDYYaCLRWLAT

                    *       120         *       140         *       160         *       180         *       200         *
CH60       : PACFFQNNTQPAYGQTRYFRFIRCGFHFRLLVNAPSGSAGALMLVWMPYPYCRVLSGANQIHTNVERRSLMNLPYAILDLRTNTEIDLVVPYVNYRNYVEITTSD :  210
A66        : PACFFQNNTQPAYGQTRYFRFIRCGFHFRLLVNAPSGSAGALMLVWMPYPYCRVLSGANQIHTNVERRSLMNLPYAILDLRTNTEIDLVVPYVNYRNYVEITTSD :  210
C80        : PACFFQNNTQPAYGQTRYFRFIRCGFHFRLLVNAPSGSAGALMLVWMPYPYCRVLSGANQIHTNVERRSLMNLPYAILDLRTNTEIDLVVPYVNYRNYVEITTSD :  210
MY         : PACFFQNNTQPAYGQTRYFRFIRCGFHFRLLVNAPSGSAGALMLVWMPYPYCRVLSGANQIHTNVERRSLMNLPYAILDLRTNTEIDLVVPYVNYRNYVEITTSD :  210
FC64       : PACFFQNNTQPAYGQTRYFRFIRCGFHFRLLVNAPSGSAGALMLVWMPYPYCRVLSGDNQIHTNVERRSLMNLPYAILDLRTNTEIDLVVPYVNYRNYVEITTSD :  210
03D        : PACFFQNNTQPAYGQTRYFRFIRCGFHFRLLVNAPSGSAGALMLVWMPYPYCRVLSGTNQIHANVERRSLMNLPYAILDLRTNTEIDLVVPYVNYRNYVEITTSD :  210
ZJ         : PACFFQNNTQPAYGQTRYFRFIRCGFHFRLLVNAPSGSAGALMLVWMPYPYCRVLSGTNQIHVNVERRSLMNLPYAILDLRTNTEIDLVVPYVNYRNYVEITTSD :  210
NA         : PACFFQNNTQPAYGQTRYFRFIRCGFHFRLLVNAPSGSAGALMLVWMPYPYCRVLSGTNQIHANVERRSLMNLPYAILDLRTNTEIDLVVPYVNYRNYVEITTSD :  210
C-XNH      : PACFFQNNTQPAYGQTRYFRFIRCGFHFRLLVNAPSGSAGALMLVWMPYPYCRVLSGSNQIHANVERRSLMNLPYAILDLRTNTEIDLVVPYVNYRNYVEITTSD :  210
LSD/090830 : PACFFQNNTQPAYGQTRYFRFIRCGFHFRLLVNAPSGSAGALMLVWMPYPYCRVLSGTNQIHANVERRSLMNLPYAILDLRTNTEIDLVVPYVNYRNYVEITTSD :  210
FZ99       : PACFFQNNTQPAYGQTRYFRFIRCGFHFRLLVNAPSGSAGALMLVWMPYPYCRVLSGTNQIHANVERRSLMNLPYAILDLRANTEIDLVVPYVNYRNYVEITTSD :  210
             PACFFQNNTQPAYGQTRYFRFIRCGFHFRLLVNAPSGSAGALMLVWMPYPYCRVLSG NQIH NVERRSLMNLPYAILDLRtNTEIDLVVPYVNYRNYVEITTSD

                     220         *       240         *       260         *       280         *       300         *
CH60       : TTGGAICVIVLGKYRHGNGTSNTVDFTLFGELLETDLQCPRPFDNQGKRKPCRRPIHKPKNPPQEPRVIIQPGPGAANLSNSSVIAMAESTALANEGTAVDYSTA :  315
A66        : TTGGAICVIVLGKYRHGNGTSNTVDFTLFGELLETDLQCPRPFDNQGKRKPCRRPIHKPKNPPQEPRVIIQPGPGAANLSNSSVIAMAESTALANEGTAVDYSTA :  315
C80        : TTGGAICVIVLGKYRHGNGTSNTVDFTLFGELLETDLQCPRPFDNQGKRKPCRRPIHKPKNPPQEPRVIIQPGPGAANLSNSSVIAMAESTALANEGTAVDYSTA :  315
MY         : TTGGAICVIVLGKYRHGNGTSNTVDFTLFGELLETDLQCPRPFDNQGKRKPCRRPIHKPKNPPQEPRVIIQPGPGAANLSNSSVIAMAESTALANEGTAVDYSTA :  315
FC64       : TTGGAICVIVLGKYRHGNGTSNTVDFTLFGELLETDLQCPRPFDNQGKRKPRRRPIHKPKNPPQEPRVIIQPGPGAANLSNSSAIAMAESTALANEGTAVDYSIA :  315
03D        : TTGGAICVVVLGKYRHGNGTSNTVDFTLFGELLETDLQCPRPFDNQGKRKPRRRPIHKPKNPPQEPRVIIQPGPGAANLSNSSVIAMAESTALANEGTAVDYSTA :  315
ZJ         : ATGGAICVIVLGKYRHGNGTSNTVDFTLFGELLETDLQCPRPFDNQGKRKPRRRPIHNPKNPPQEPRVIIQPGPGAANLSNSSVIAMAESTALANEGTAVDYSTA :  315
NA         : ATGGAICVIVLGKYQHGNGTSNTVDFTLFGELLETDLQCPRPFDNQGKRKPRRRPIHNPKNPPQEPRVIIQPGPGAANLSNSSVIAMAESTALANEGTAVDYSTA :  315
C-XNH      : TTGGAICVIVLGKYRHGNGTSNTVDFTLFGELLETDLQCPRPFDNQGKRKPRRRPIHKPKNPPQEPRVIIQPGPGAANLSNSSVIAMAESTALANEGTAVDYSTA :  315
LSD/090830 : TTGGAICVIVLGKYRHGNGTSNTVDFTLFGELLETDLQCPRPFDNQGKRKPRRRPIHKPKNPPQEPRVIIQPGPGAANLSNSSVIAMAESTALANEGTAVDYSTA :  315
FZ99       : ATGGAICVIVLGKYRHGNGTSNTVDFTLFGELLETDLQCPRPFDNQGKRKPRRRPIHKPKNPPQEPRVIIQPGPGAANLSNSSVIAMAESTALANEGTAVDYSTA :  315
              TGGAICV6VLGKYrHGNGTSNTVDFTLFGELLETDLQCPRPFDNQGKRKP RRPIHkPKNPPQEPRVIIQPGPGAANLSNSSvIAMAESTALANEGTAVDYStA

               320         *       340         *       360         *       380         *       400         *       420
CH60       : GCASSVDDVIMVLRRWQILASFQWQSTLTPSARINRYQLIFRNIPTFSLFFDKFQYWRGSLEVKFMTFGSQFNTGRYQMSWYPIADGEQSLAQCQNSVFVTGDVC :  420
A66        : GCASSVDDVIMVLRRWQIPASFQWQSTLTPNARINRYQLIFRNIPTFSLFFDKFQYWRGSLEVKFMTFGSQFNTGRYQMSWYPIADGEQSLAQCQNSVFVTGDVC :  420
C80        : GCASSVDDVIMVLRRWQILASFQWQSTLTPNARINRYQLIFRNIPTFSLFFDKFQYWRGSLEVKFMTFGSQFNTGRYQMSWYPIADGEQSLAQCQNSVFVTGDVC :  420
MY         : GCASSVDDVIMVLRRWQILASFQWQSTLTPSDRINRYQLIFRNIPTFSLFFDKFQYWRGSLEVKFMTFGSQFNTGRYQMSWYPIADGEQSLAQCQNSVFVTGDVC :  420
FC64       : GCASSVDDVIMVLRRWQILASFQWQSTLTPSARINRYQLIFRNIPTFSLFFDKFQYWRGSLEVKFMTFGSQFNTGRYQMSWYPIADGEQSLAQCQNSVFVTGDVC :  420
03D        : GCASSVDDVIMVLRRWQILASFQWQSTLTPSVRINRYQLIFRNIPTFSLFFDKFQYWRGSLEVKFMTFGSQFNTGRYQMSWYPIADGEQSLAQCQNSVFVTGDVC :  420
ZJ         : GCASSVDDVIMVLRRWQILASFQWQSTQTPSTRINRYLLIFRNIPTFSLFFDKFQYWRGSLEVKFMTFGSQFNTGRYQMSWYPIADGEQSLAQCQNSVFVTGDVC :  420
NA         : GCASSVDDVIMVLRRWQILASFQWQSTQTPSTRINRYQLIFRNIPTFSLFFDKFQYWRGSLEVKFMTFGSQFNTGRYQMSWYPIADGEQSLAQCQNSVFVTGDVC :  420
C-XNH      : GCASSVDDVIMVLRRWQILASFQWQSTQTPSTRINRYQLIFRNIPTFSLFFDKFQYWRGSLEVKFMTFGSQFNTGRYQMSWYPIADGEQSLAQCQNSVFVTGDVC :  420
LSD/090830 : GCASSVDDVIMVLRRWQILASFQWQSTLTPSARINRYQLIFRNIPTFSLFFDKFQYWRGSLEVKFMTFGSQFNTGRYQMSWYPIADGEQSLAQCQNSVFVTGDVC :  420
FZ99       : GCASSVDDVIMVLRRWQILASFQWQSTQTPSTRINRYQLIFRNIPTFTLFFDKFQYWRGSLEVKFMTFGSQFNTGRYQMSWYPIADGEQSLAQCQNSVFVTGDVC :  420
             GCASSVDDVIMVLRRWQIlASFQWQST TPs RINRYqLIFRNIPTF3LFFDKFQYWRGSLEVKFMTFGSQFNTGRYQMSWYPIADGEQSLAQCQNSVFVTGDVC

                      *       440         *       460         *       480         *       500         *       520
CH60       : ATPVTLTLPFTSTTWRKSTRDPYGYLMWHVVNRLTVNSSAPSNIDCTVLLRVGKDFQFTAPLYGNLQMATNNQGDTNQLGDDEPVCFLNFETANVPIQGESHTLV :  525
A66        : ATPVTLTLPFTSTTWRKSTRDPYGYLMWHVVNRLTVNSSAPSNIDCTVLLRVGKDFQFTAPLYGNLQMATNNQGDTNQLGDDEPVCFLNFETANVPIQGESHTLV :  525
C80        : ATPVTLTLPFTSTTWRKSTRDPYGYLMWHVVNRLTVNSSAPSNIDCTVLLRVGKDFQFTAPLYGNLQMATNNQGDTNQLGDDEPVCFLNFETANVPIQGESHTLV :  525
MY         : ATPVTLTLPFTSTTWRKSTRDPYGYLMWHVVNRLTVNSSAPSNIDCTVLLRVGKDFQFTAPLYGNLQMATNNQGDTNQLGDDEPVCFLNFETANVPIQGESHTLV :  525
FC64       : ATPVTLTLPFTSTTWRKSTRDPYGYLMWHVVNRLTVNSSAPSNIDCTVLLRVGKDFQFTAPLYGNLQMATNNQGDTNQLGDDEPVCFLNFETANVPIQGESHTLV :  525
03D        : ATPVTLTLPFTSTTWRKSTRDPYGYLMWHVVNRLTVNSSAPSNIDCTVLLRVGKDFQFTAPLYGNLQMATNNQGDSNQLGDDEPVCFLNFETANVPIQGESHTLV :  525
ZJ         : ATPVTLTLPFTSTTWRKSTRDPYGYLMWHVVNRLTVNSSAPSNIDCTVLLRVGKDFQFTAPLYGNLQMATNNQGDSNQLGDDEPVCFLNFETANVPIQGESHTLV :  525
NA         : ATPVTLTLPFTSTTWRKSTRDPYGYLMWHVVNRLTVNSSAPSNIDCTVLLRVGKDFQFTAPLYGNLQMATNNQGDSNQLGDDEPVCFLNFETANVPIQGESHTLV :  525
C-XNH      : ATPVTLTLPFTSTTWRKSTRDPYGYLMWHVVNRLTVNSSAPSNIDCTVLLRVGKDFQFTAPLYGNLQMATNNQGDSNQLGDDEPVCFLNFETANVPIQGESHTLV :  525
LSD/090830 : ATPVTLTLPFTSTTWRKSTRDPYGYLMWHVVNRLTVNSSAPSNIDCTVLLRVGKDFQFTAPLYGNLQMATNNQGDSNQLGDDEPVCFLNFETANVPIQGESHTLV :  525
FZ99       : ATPVTLTLPFTSTTWRKSTRDPYGYLMWHVVNRLTVNSSAPSNIDCTVLLRVGKDFQFTAPLYGNLQMATNNQGDSNQLGDDEPVCFLNFETANVPIQGESHTLV :  525
             ATPVTLTLPFTSTTWRKSTRDPYGYLMWHVVNRLTVNSSAPSNIDCTVLLRVGKDFQFTAPLYGNLQMATNNQGD3NQLGDDEPVCFLNFETANVPIQGESHTLV

                    *       540         *       560         *       580         *       600         *       620         *
CH60       : KHLFGRQWLVRTVQHASTVQELDLQVPDRGHASLIRFFAYFSGEIILTIVNNGTTPAMVAHSYSMDDLSSEYAVTAMGGVMIPANSAKNISVPFYSVTPLRPTRP :  630
A66        : KHLSGRQWLVRTVQHASTVQELDLQVPDRGHASLIRFFAYFSGEIILTIVNNGTTPAMVAHSYSMDDLSSEYAVTAMGGVMIPANSAKNISVPFYSVTPLRPTRP :  630
C80        : KHLFGRQWLVRTVQHASTVQELDLQVPDRGHASLIRFFAYFSGEIILTIVNNGTTPAMVAHSYSMDDLSSEYAVTAMGGVMIPANSAKNISVPFYSVTPLRPTRP :  630
MY         : KHLFGRQWLVRTVQHASTVQELDLQVPDRGHASLIRFFAYFSGEIILTIVNNGTTPAMVAHSYSMDDLSSEYAVTAMGGVMIPANSAKNISVPFYSVTPLRPTRP :  630
FC64       : KHLFGRQWLVRTVQHASTVQELDLQVPDRGHASLIRFFAYFSGEIILTIVNNGTTPAMVAHSYSMDDLSSEYAVTAMGGVMIPANSAKNISVPFYSVTPLRPTRP :  630
03D        : KHLFGRQWLVRTVQHAPTVQELDLQVPDRGHASLIRFFAYFSGEIILTIVNNGTTPAMVAHSYSMDDLSSEYAVTAMGGVMIPANSAKNISVPFYSVTPLRPTRP :  630
ZJ         : KHLFGRQWLVRTVQHTSTVQELDLQVPDRGHASLIRFFAYFSGEIILTIVNNGTTPAMVAHSYSMDDLTSEYAVTAMGGVMIPANSAKNISVPFYSVTPLRPTRP :  630
NA         : KHLFGRQWLVRTVQHASTVQELDLQVPDRGHASLIRFFAYFSGEIILTIVNNGTTPAMVAHSYSMDDLTSEYAVTAMGGVMIPANSAKNISVPFYSVTPLRPTRP :  630
C-XNH      : KHLFGRQWLVRTVQHASTVQELDLQVPDRGHASPIRFFAYFSGEIILTIVNNGTTPAMVAHSYSMDDLSSEYAVTAMGGVMIPANSAKNISVPFYSVTPLRPTRP :  630
LSD/090830 : KHLFGRQWLVRTVQHALTVQELDLQVPDRGHASLIRFFAYFSGEIILTIVNNGTTPAMVAHSYSMDDLSSEYAVTAMGGVMIPANSAKNISVPFYSVTPLRPTRP :  630
FZ99       : KHLFGRQWLVRTVQHTSTVQELDLQVPDRGHASLIRFFAYFSGEIILTIVNNGTTPAMVAHSYSMDDLSSEYAVTAMGGVMIPANSAKNISVPFYSVTPLRPTRP :  630
             KHLfGRQWLVRTVQHasTVQELDLQVPDRGHASlIRFFAYFSGEIILTIVNNGTTPAMVAHSYSMDDL3SEYAVTAMGGVMIPANSAKNISVPFYSVTPLRPTRP

                     640         *       660         *       680         *       700         *       720         *
CH60       : IPGTSEATFGRLFMWTQSGSLSVFMGLKKPALFFPLPAPTSTTLSRGSNSVIPTLDQSGDEVDCHFCKICSKMKRMWKPKGHFRFCLRLKTLAFELDLEIESDQI :  735
A66        : IPGTSEATFGRLFMWTQSGSLSVFMGLKKPALFFPLPAPTSTTLSRGSNGVIPTLDQSGDEVDCHFCKICSKMKRMWKPKGHFRFCLRLKTLAFELDLEIESDQI :  735
C80        : IPGTSEATFGRLFMWTQSGSLSVFMGLKKPALFFPLPAPTSTTLSRGSNGVIPTLDQSGDEVDCHFCKICSKMKRMWKPKGHFRFCLRLKTLAFELDLEIESDQI :  735
MY         : IPGTSEATFGRLFMWTQSGSLSVFMGLKKPALFFPLPAPTSTTLSRGSNSVIPTLDQSGDEVDCHFCKICSKMKRMWKPKGHFRFCLRLKTLAFELDLEIESDQI :  735
FC64       : IPGTSEATFGRLFMWTQSGSLSVFMGLKKPALFFPLPAPTSTTLSRGSNGFIPTLDQSGDEVDCHFCKICSKMKRMWKPKGYFRFCLRLKTLAFELDLEIESDQI :  735
03D        : IPGTSEATFGRLFMWTQSGSLSVFMGLKKPALFFPLPAPTSTTTSRGSNGVIPTLDQSGDEVDCHFCEICSKMKRMWKPRGHFRFCLRLKTLAFELNLEIESDQI :  735
ZJ         : IPGTSEATFGRLFMWTQSGSLSVFMGLKKPALFFPLPAPTSTTLSRKSNDVIPTSDQSGDEVDCHFCEICSKMKRMWKPRGHFRFCLRLKTLAFELNLEIESDQI :  735
NA         : IPGTSEATFGRLFMWTQSGSLSVFMGLKKPALFFPLPAPTSTTLARKSNDVIPTSDQSGDEVDCHFCEICSKMKRMWKPRGHFRFCLRLKTLAFELNLEIESDQI :  735
C-XNH      : IPGTSEATFGRLFMWTQSGSLSVFMGLKKPALFFPLPAPTSTTLSRKSNDVIPTLDQSGDEVDCHFCKICSKMKRMWKPRGHFRFCLRLKTLAFELNLEIESDQI :  735
LSD/090830 : IPGTSEATFGRLFMWTQSGSLSVFMGLKKPALFFPLPAPTSTTLSRKSNDVIPTLDQSGDEVDCHFCEICSKMKRRMWKPRGRFRFCLRLKTLAFELNLELESDQI :  735
FZ99       : IPGTSEATFGRLFMWTQSGSLSVFMGLKKPALFFPLPAPTSTTLSRKSNDVIPTLDQSGDEVDCHFCEICSKMKRMWKPRGHFRFCLRLKTLAFELNLEIESDQI :  735
             IPGTSEATFGRLFMWTQSGSLSVFMGLKKPALFFPLPAPTSTTlsR SN vIPTlDQSGDEVDCHFC ICSKM4RMWKP4GhFRFCLRLKTLAFEL1LE6ESDQI

               740         *       760         *       780         *       800         *       820         *       840
CH60       : RNKKDLTTEGVEPNPGPILVVGKSGSGKSRLCNVLAGVELFESKLSPRSVTMVHQVETIDIEGKKVTIVDSPETPNYDGPISAFFYLIEAGRFTAEDKEYIQMMR :  840
```

```
A66        : RNKKDLTTEGVEPNPGPILVVGKSGSGKSRLCNVLAGVELFESKLSPRSVTMVHQVETIDIEGKKVTIVDSPETPNYDGPISAFFYLIEAGRFTAEDKEYIQMMR :  840
C80        : RNKKDLTTEGVEPNPGPILVVGKSGSGKSRLCNVLAGVELFESKLSPRSVTMVHQVETIDIEGKKVTIVDSPETPNYDGPISAFFYLIEAGRFTAEDKEYIQMMR :  840
MY         : RNKKDLTTEGVEPNPGPILVVGKSGSGKSRLCNVLAGVELFESKLSPRSVTMVHQVETIDIEGKKVTIVDSPETPNYDGPISAFFYLIEAGRFTAEDKEYIQMMR :  840
FC64       : RNKKDLTTEGVEPNPGPILVVGKSGSGKSRLCNVLAGVELFGSKLSPRSVTMVHQVETIDIEGKKVTIVDSPETPNYDGPISAFFYLIEAGRFTAEDKEYIQMMR :  840
03D        : RNKKDLTTEGVEPNPGPILVVGKSGSGKSRLCNVLAGVELFESKLSPRSVTMVHQVETIDIEGKKVTIVDSPETPNYDGPISAFFYLIEAGRFTAEDKEYIQMMR :  840
ZJ         : RNKKDLTTEGVEPNPGPILVVGKSGSGKSRLCNVLAGVELFESKLSPRSVTMVHQVETIDVEGKKVTIVDSPETPNYDGPISAFFYLIEAGRFTAEDKEYIQMMR :  840
NA         : RNKKDLTTEGVEPNPGPILVVGKSGSGKSRLCNVLAGVELFESKLSPRSVTMIHQVETIDVEGKKVTIVDSPETPNYDGPISAFFYLIEAGRFTAEDKEYIQMMR :  840
C-XNH      : RNKKDLTTEGVEPNPGPILVVGKSGSGKSRLCNVLAGVELFESKLSPRSVTMVHQVETIDVEGKKVTIVDSPETPNYDGPISAFFYLIEAGRFTVEDKEYVQMMR :  840
LSD/090830 : RNKKDLTTEGVEPNPGPILVVGKSGSGKSRLCNVLAGVELFESKLSPCSVTMVHQVETIDVEGKKVTIVDSPETPNYDGPISAFFYLIEAGRFTAEDKEYIQMMR :  840
FZ99       : RNKKDLTTEGVEPNPGPILVVGKSGSGKSRLCNVLAGVELFESKLSPRSVTMVHQVETIDVEGKKVTIVDSPETPNYDGPISAFFYLIEAGRFTAEDKEYIQMMR :  840
             RNKKDLTTEGVEPNPGPILVVGKSGSGKSRLCNVLAGVELFeSKLSPrSVTM6HQVETID6EGKKVTIVDSPETPNYDGPISAFFYLIEAGRFTaEDKEY6QMMR

                    *        860       *        880       *        900       *        920       *        940
CH60       : KRFPSFEKSTILILNRADELKTDDDIRNWIKTSGELDSLVRACGGRIAKFHRYKINRAKLLEKAATLPEFVSHLPRLVYKDRKMYRHYGVQCGSTVFHMDSENIL :  945
A66        : KRFPSFEKSTILILNRADELKTDDDIRNWIKTSGELDSLVRACGGRIAKFHRYKINRAKLLEKAATLPEFVSHLPRLVYKDRKMYRHYGVQCGSTVFHMDSENIL :  945
C80        : KRFPSFEKSTILILNRADELKTDDDIRNWIKTSGELDSLVRACGGRIAKFHRYKINRAKLLEKAATLPEFVSHLPRLVYKDRKMYRHYGVQCGSTVFHMDSENIL :  945
MY         : KRFPSFEKSTILILNRADELKTDDDIRNWIKTSGELDSLVRACGGRIAKFHRYKINRAKLLEKAATLPEFVSHLPRLVYKDRKMYRHYGVQCGSTVFHMDSENIL :  945
FC64       : KRFPSFEKSTILILNRADELKTDDDIRNWIKTSGELDSLVRACGGRIAKFHRYKINRAKLLEKAATLPEFVSHLPRLVYKDRKMYRHYGVQCGSTVFHMDSENIL :  945
03D        : KRFPGFEKSTILILNRADELKTDDDIRNWVKTSGELDSLVRACGGRIAKFHRHKINRAKLLEKAATLPEFVSHLPRLVYKDRKMYRHYGVQCGSTVFHMDSENIL :  945
ZJ         : KRFPGFEKSTILILNRADELKTDDDIRKWIKTSGELDSLVRACGGRIAKLYRHKINRTKLLEKAATLPEFVSHLPRLVYKDRRMYRHYGVQCGSTVFHMDSENIL :  945
NA         : KRFPGFEKSTILILNRADELKTDDDIRKWVKTSGELDSLVRACGGRIAKFHRHKINRTKLLEKAATLPEFVSHLPRLVYKDRRMYRHYGVQCGSTVFHMDSENIL :  945
C-XNH      : KRFPGFEKSTILILNRASELKTDDDIRKWVKTSGELDSLVRACGGRIAKFYWHKINRTKLLEKAATLPEFVSHLPRLVYKDRRIYRHYGVQCGSTVFHMDSENIL :  945
LSD/090830 : KRFPGFEKSTILILNRADELKTDNDIRNWVKTSGELDSLVRACGGRIAKFHRHKINRAKLLEKAATLPEFVSHLPRLVYKDRKMYRHYGVQCGSTVFHMDSENIL :  945
FZ99       : KRFPGFEKSTILILNRADELKTDDDIRKWVKTSGELDSLVRACGGRIAKFYRHKINRAKLLEKAATLPEFVSHLPRLVYKDRRMYRHYGVQCGSTVFHMDSENIL :  945
             KRFP FEKSTILILNRAdELKTD1DIR W6KTSGELDSLVRACGGRIAKf r KINR KLLEKAATLPEFVSHLPRLVYKDR46YRHYGVQCGSTVFHMDSENIL

                  *        960       *        980       *       1000       *       1020       *       1040       *
CH60       : ESALNGEVTIKQEKWNGNWKPAGDHMQSTASMYLKSQTMPKFTFSIDENCETWARHLLGDYGETQGQIFKERLMWAVALGFFMTMKITTDQSFPGKDAIHTVLTK : 1050
A66        : ESALNGEVTIKQEKWNGNWKPAGDHMQSTASMYLKSQTMPKFTFSIDENCETWARHLLGDYGETQGQIFKERLMWAVALGFFMTMKITTDQSFPGKDAIHTVLTK : 1050
C80        : ESALNGEVTIKQEKWNGNWKPAGDHMQSTASMYLKSQTMPKFTFSIDENCETWARHLLGDYGETQGQIFKERLMWAVALGFFMTMKITTDQSFPGKDAIHTVLTK : 1050
MY         : ESALNGEVTIKQEKWNGNWKPAGDHMQSTASMYLKSQTMPKFTFSIDENCETWARHLLGDYGETQGQIFKERLMWAVALGFFMTMKITTDQSFPGKDAIHTVLTK : 1050
FC64       : ESALNGEVTIKQEKWNGNWKPAGDHMQSTASMYLKSQTMPKFTFSIDENCETWARHLLGDYGETQGQIFKERLMWAVALGFFMTMKITTDQSFPGKDAIHTVLTK : 1050
03D        : ESALNGEVTIKQEKWNGNWKPAGDHMQSTASMYLKSQTMPKFTFSIDENCETWARHLLGDYGETQGQIFKERLMWAAALGFFMTMKITTDQSFPGKDAIHTVLTK : 1050
ZJ         : ESALNGEVTIKQEKWNGNWKPAGDHMQSTASMYLKSQTMPKFTFSIDENCETWARHLLGDYGETQGQIFKERLMWAAALGFFMTMKITTDQSFPGKDAIHTVLTK : 1050
NA         : ESALNGEVTIKQEKWNGNWKPAGDHMQSTASMYLKSQTMPKFTFSIDENCETWARHLLGDYGETQGQIFKERLMWAAALGFFMTMKITTDQSFPGKDAIHTVLTK : 1050
C-XNH      : ESALNGEVTIKQEKWNGNWKPAGDHMQSTASMYLKSQTMPKFTFSIDENCETWARHLLGDYGETQGQIFKERLMWAAALGFFMTMKITTDQSFPGKDAIHTVLTK : 1050
LSD/090830 : ESALNGEVTIKQEKWNGNWKPAGDHMQSTASMYLKSQTMPKFAFSIDENCETWARHLLGDYGETQGQILKERLMWAAALGFFMTMKITTDQSFPGKDAIHTVLTK : 1050
FZ99       : ESALNGEVTIKQEKWNGNWKPAGDHMQSTASMYLKSQTMPKFTFSIDENCETWARHLLGDYGETQGQIFKERLMWAVALGFFMTMKITTDQSFPGKDAIHTVLTK : 1050
             ESALNGEVTIKQEKWNGNWKPAGDHMQSTASMYLKSQTMPKFtFSIDENCETWARHLLGDYGETQGQIfKERLMWA ALGFFMTMKITTDQSFPGKDAIHTVLTK

                   1060       *       1080       *       1100       *       1120       *       1140       *
CH60       : ISNFIFGGLENEVVRVVIRTVIRIVCYLILYIHSPNIVTTGTLVALLALDATSMSMDQGLKTLCMSLVDGDFGKFCSALLEKIQTVDEADLKKTIPKFNDMLEDQ : 1155
A66        : ISNFIFGGLENEVVRVVIRTVIRIVCYLILYIHSPNIVTTGTLVALLALDATSMSMDQGLKTLCMSLVDGDFGKFCSALLEKIQTVDEADLKKTIPKFNDMLEDQ : 1155
C80        : ISNFIFGGLENEVVRVVIRTVIRIVCYLILYIHSPNIVTTGTLVALLALDATSMSMDQGLKTLCMSLVDGDFGKFCSALLEKIQTVDEADLKKTIPKFNDMLEDQ : 1155
MY         : ISNFIFGGLENEVVRVVIRTVIRIVCYLILYIHSPNIVTTGTLVALLALDATSMSMDQGLKTLCMSLVDGDFGKFCSALLEKIQTVDEADLKKTIPKFNDMLEDQ : 1155
FC64       : ISNFIFGGLENEVVRVVIRTVIRIVCYLILYIHSPNIVTTGTLVALLALDATSMSMDQGLKTLCMSLVDGDFGKFCSALLEKIQTVDEADLKKTIPKFNDMLEDQ : 1155
03D        : ISNFIFGGLENEVVRVVIRTVIRIVCYLILYIHSPNIVTTGTLVALLALDATSMSMDQGLKTLCMSLVDGDFGKFCSALLEKIQIVDEADLKKTIPKFNDMLEDQ : 1155
ZJ         : ISNFIFGGLENEVVRVVIRTVIRIVCYLILYIHSPNIVTTGTLVALLALDATSMSMDQGLKTLCMSLVDGDFGKFCSALLEKIQTVDEADLKKTIPKFNDMLEDQ : 1155
NA         : ISNFIFGGLENEVVRIVIRTVIRIVCYLILYIHSPNIVTTGTLVALLALDATSMSMDQGLKTLCMSLVDGDFGKFCSALLEKIQTVDEADLKKTIPKFNDMLEDQ : 1155
C-XNH      : ISNFIFGGLENEVVRVVIRTVIRIVCYLILYIHSPNIVTTGTLVALLALDATSMSMDQGLKTLCMSLVDGDFGKFCSALLEKIQTVDEADLKKTIPKFNDMLEDQ : 1155
LSD/090830 : ISNFIFGGLENEVVRVVIRTVIRIVCYLILYIHSPNIVTTGTLVALLALDATSMSMDQGLKTLCMSLVDGDFGKFCSALLEKIQTVDEADLKKTIPKFNGMLEDQ : 1155
FZ99       : ISNFIFGGLENEVVRVVIRTVIRIVCYLILYIHSPNIVTTGTLVALLALDATSMSMDQGLKTLCMSLVDGDFGKFCSALLEKIQTVDEADLKKTIPKFNDMLEDQ : 1155
             ISNFIFGGLENEVVR6VIRTVIRIVCYLILYIHSPNIVTTGTLVALLALDATSMSMDQGLKTLCMSLVDGDFGKFCSALLEKIQtVDEADLKKTIPKFNdMLEDQ

               1160       *       1180       *       1200       *       1220       *       1240       *       1260
CH60       : SGKTTSPKSFNDWTTCAKNVQWWLESFVKVVNWLKEKIFPSKTDPTLQWLQDHEEHIAIMLALCDEHLCMLRTEKDYICEHNTRPKHQCLVEMVSGTLNQLQGIS : 1260
A66        : SGKTTSPKSFNDWTTCAKNVQWWLESFVKVVNWLKEKIFPSKTDPTLQWLQDHEEHIAIMLALCDEHLCMLRTEKDYICEHNTRPKHQCLVEMVSGTLNQLQGIS : 1260
C80        : SGKTTSPKSFNDWTTCAKNVQWWLESFVKVVNWLKEKIFPSKTDPTLQWLQDHEEHIAIMLALCDEHLCMLRTEKDYICEHNTRPKHQCLVEMVSGTLNQLQGIS : 1260
MY         : SGKTTSPKSFNDWTTCAKNVQWWLESFVKVVNWLKEKIFPSKTDPTLQWLQDHEEHIAIMLALCDEHLCMLRTEKDYICEHNTRPKHQCLVEMVSGTLNQLQGIS : 1260
FC64       : SGKTTSPKSFNDWTTCAKNVQWWLESFVKVVNWLKEKIFPSKTDPTLQWLQDHEEHIAIMLALCDEHLCMLRTEKDYICEHNTRPKHQCLVEMVSGTLNQLQGIS : 1260
03D        : SGKATSPKSFNDWTTCAKNVQWWLESFVKVVNWLKEKVFPSKTDPTLQWLQDHEEHIAIMLALCDEHLCMLRTEKDYICEHNTRPKHQRLVEMVSGTLNQLQGIS : 1260
ZJ         : SGKATSPKSFNDWTTCAKNVQWWLESFVKVVNWLKEKVFPSKTDPTLQWLQDHEEHIAIMLALCDEHLCMLRTEKDYICEHNTRPKHQRLVEMVSGTLNQLQGIS : 1260
NA         : SGKATSPKSFNDWTTCAKNVQWWLESFVKVVNWLKEKVFPSKTDPTLQWLQDHEEHIAIMLALCDEHLCMLRTEKDYICEHNTRPKHQRLVEMVSGTLNQLQGIS : 1260
C-XNH      : SGKTTSPKSFNDWTTCAKNVQWWLESFVKVVNWLKEKVFPSKTDPTLQWLQDHEEHIAIMLALCDEHLCMLRTEKDYICEHNTRPKHQRLVEMVSGTLNQLQGIS : 1260
LSD/090830 : SGKATSPKSFNDWTTCAKNVQWWLESFVKVVNWLKEKVFPSKTDPTLQWLQDHEEHIAIMLALCDEHLCMLRTEKDYICEHNTRPKHQRLVEMVSGTLNQLQGIS : 1260
FZ99       : SGKATSPKSFNDWTTCAKNVQWWLESFVKVVNWLKEKVFPSKTDPTLQWLQDHEEHIAIMLALCDEHLCMLRTEKDYICEHNTRPKHQRLVEMVSGTLNQLQGIS : 1260
             SGK TSPKSFNDWTTCAKNVQWWLESFVKVVNWLKEK6FPSKTDPTLQWLQDHEEHIAIMLALCDEHLCMLRTEKDYICEHNTRPKHQ LVEMVSGTLNQLQGIS

                    *       1280       *       1300       *       1320       *       1340       *       1360
CH60       : SARELAARLQHVLNKLHQVNFEPELEWTHRPEPLGIWISGGPGVGKSFLSNYIVKEIAKLKHWKSYANPTGSKHMDGYVSQEIHVFDDFGQNREEEDYSLICNLI : 1365
A66        : SARELAARLQHVLNKLHQVNFEPELEWTHRPEPLGIWISGGPGVGKSFLSNYIVKEIAKLKHWKSYANPTGSKHMDGYVSQEIHVFDDFGQNREEEDYSLICDLI : 1365
C80        : SARELAARLQHVLNKLHQVNFEPELEWTHRPEPLGIWISGGPGVGKSFLSNYIVKEIAKLKHWKSYANPTGSKHMDGYVSQEIHVFDDFGQNREEEDYSLICNLI : 1365
MY         : SARELAARLQHVLNKLHQVNFEPELEWTHRPEPLGIWISGGPGVGKSFLSNYIVKEIAKLKHWKSYANPTGSKHMDGYVSQEIHVFDDFGQNREEEDYSLICNLI : 1365
FC64       : SARELAARLQHVLNKLHQVNFEPELEWTHRPEPLGIWISGGPGVGKSFLSNYIVKEIAKLKHWKSYANPTGSKHMDGYVSQEIHVFDDFGQNREEEDYSLICNLI : 1365
03D        : SARELAARLQHVLNKLHQVNFEPELEWTHRPEPLGIWISGGPGVGKSFLSNYIVKEIAKLKHWKSYANPTGSKHMDGYVSQEIHVFDDFGQNREEEDYSLICNLI : 1365
ZJ         : SARELAARLQHVLNKLHQVNFEPELEWTHRPEPLGIWISGGPGVGKSFLSNYIVKEIAKLKHWKSYANPTGSKHMDGYVSQEIHVFDDFGQNREEEDYSLICNLI : 1365
NA         : SARELAARLQHVLNKLHQVNFEPELEWTHRPEPLGIWISGGPGVGKSFLSNYIVKEIAKLKHWKSYANPTGSKHMDGYVSQEIHVFDDFGQNREEEDYSLICNLI : 1365
C-XNH      : SARELAARLQHVLNKLHQVNFEPELEWTHRPEPLGIWISGGPGVGKSFLSNYIVKEIAKLKHWKSYANPTGSKHMDGYVSQEIHVFDDFGQNREEEDYSLICNLI : 1365
LSD/090830 : SARELAARLQHVLNKLHQVNFEPELEWTHRPEPLGIWISGGPGVGKSFLSNYIVKEIAKLKHWKSYANPTGSKHMDGYVSQEIHVFDDFGQNREEEDYSLICNLI : 1365
FZ99       : SARELAARLQHVLNKLHQVNFEPELEWTHRPEPLGIWISGGPGVGKSFLSNYIVKEIAKLKHWKSYANPTGSKHMDGYVSQEIHVFDDFGQNREEEDYSLICNLI : 1365
             SARELAARLQHVLNKLHQVNFEPELEWTHRPEPLGIWISGGPGVGKSFLSNYIVKEIAKLKHWKSYANPTGSKHMDGYVSQEIHVFDDFGQNREEEDYSLIC1LI

                  *       1380       *       1400       *       1420       *       1440       *       1460       *
CH60       : SSVPFITPKASVEAKGTQYRGRLVVVTTNRRDFTSCKLTDPDALERRFPIRLNIRPLQKYNHKGRLDVATAMRDGSLQGGACWERDIGQLGLECWNPINGQTLID : 1470
A66        : SSVPFITPKASVEAKGTQYRGRLVVVTTNRRGFTSCKLTDPEALERRFPIRLNIRPLQKYNHKGRLDVATAMRDGSLQGGACWERDIGQLGLECWNPINGQTLID : 1470
C80        : SSVPFITPKASVEAKGTQYRGRLVVVTTNRRDFTSCKLTDPDALERRFPIRLNIRPLQKYNHKGRLDVATAMRDGSLQGGACWERDIGQLGLECWNPINGQTLID : 1470
MY         : SSVPFITPKASVEAKGTQYRGRLVVVTTNRRDFTSCKLTDPDALERRFPIRLNIRPLQKYNHKGRLDVATAMRDGSLQGGACWERDIGQLGLECWNPINGQTLID : 1470
FC64       : SSVPFITPKASVEAKGTQYRGRLVVVTTNRRDFTSCKLTDPDALERRFPIRLNIRPLQKYNHKGRLDVATAMRDGSLQGGACWERDIGQLGLECWNPINGQTLID : 1470
03D        : SSVPFITPKASVEAKGTQYRGRLVVVTTNRRDFTSCKLTDPDALERRFPIRLNIRPLQKYNHKGRLDVATAMRDGSLQGGTCWERDIGQLGLECWNPINGQILID : 1470
ZJ         : SSVPFITPKASVEAKGTQYRGRLVVVTTNRRDFTSCKLTDPDALERRFPIRLNIRPLQKYNHKGRLDVATAMRDGSLQGGTCWERDIGQLGLECWNPINGQTLID : 1470
NA         : SSVPFITPKASVEAKGTQYRGRLVVVTTNRRDFTSCKLTDPDALERRFPIRLNIRPLQKYNHKGRLDVATAMRDGSLQGGTCWERDIGQLGLECWNPINGQTLID : 1470
C-XNH      : SSVPFITPKASVEAKGTQYRGRLVVVTTNRRDFTSCKLTDPDALERRFPIRLNIRPLQKYNHKGRLDVATAMRDGSLQGGTCWERDIGQLGLECWNPINGQTLID : 1470
LSD/090830 : SSVPFITPKASVEAKGTQYRGRLVVVTTNRRDFTSCKLTDPDALERRFPFRLNIRPLQKYNHKGRLDVATAMRDGSLQGGTCWERDIGQLGLECWNPINGQTLID : 1470
FZ99       : SSVPFITPKASVEAKGTQYRGRLVVVTTNRRDFTSCKLTDPDALERRFPIRLNIRPLQKYNHKGRLDVATAMRDGSLQGGTCWVRDIGQLGLECWNPINGQTLID : 1470
             SSVPFITPKASVEAKGTQYRGRLVVVTTNRRdFTSCKLTDPdALERRFPiRLNIRPLQKYNHKGRLDVATAMRDGSLQGG CWeRDIGQLGLECWNPINGQtLID

                   1480       *       1500       *       1520       *       1540       *       1560       *
CH60       : EIMSELQVRQEVASFMNQSKVRRFSDPETLFSDLEDLKLEFDFDQLEQQAKLFAKPKEGKISKFRAWVRDCTGKIKGFLERNRAWILGIGTLGTIMSLVTMCVPL : 1575
A66        : EIMSELQVRQEVASFMNQSKVRRSSDPETLFSDLEDLKLEFDFDQLEQQAKLFAKPKEGKISKFRAWVRDCTGKIKGFLERNRAWILGIGTLGTIKSLVTMCVPL : 1575
C80        : EIMSELQVRQEVASFMNQSKVRRFSDPETLFSDLEDLKLEFDFDQLEQQAKLFAKPKEGKISKFRAWVRDCTGKIKGFLERNRAWILGIGTLGTIMSLVTMCVPL : 1575
MY         : EIMSELQVRQEVASFMNQSKARRFSDPETLFSDLEDLKLEFDFDQLEQQAKLFAKPKEGKISKFRAWVRDCTGKIKGFLERNRAWILGIGTLGTIMSLVTMCVPL : 1575
```

```
FC64       : EIMSELQVRQEVASFMNQSNVRRFSDPETLFSDLEDLKLEFDFDQLEQQAKLFAKPKEGKISKFRAWVRDCTGKIKGFLERNRAWILGIGTLGTIMSLVTMCVPL : 1575
03D        : EIMSELQVRQEVASFMNQSKVRRFSDPETLFSDLEDLKLEFDFDQLEQQAKLFAKPKEGKISKFRAWVRDCTGKIKGFLERNRAWILGIGTLGTIMSLVTMCVPL : 1575
ZJ         : EIMSELQVRQEVASFMNQSKVRRFSDPETLFSDLEDLKLEFDFDQLEQQAKLFAKPKEGKISKFRAWVRDCTGKIKGFLERNRAWILGIGTLGTIMSLVTMCVPL : 1575
NA         : EIMSELQVRQEVASFMNQSKVRRFSDPETLFSDLEDLKLEFDFDQLEQQAKLFAKPKEGKISKFRAWVRDCTGKIKGFLERNRAWILGIGTLGTIMSLVTMCVPL : 1575
C-XNH      : EIMSELQVRQEVASFMNQSKVRRFSDPETLFSDLEDLKLEFDFDQLEQQAKLFAKPKEGKISKFRAWVRDCTGKIKGFLERNRAWILGIGTLGTIMSLVTMCVPL : 1575
LSD/090830 : EIMSELQVRQEVASFMNQSKVRRFSDPETLFSDLEDLKLEFDFDQLEQQAKLFAKPKEGKISKFRAWVRDCTGKIKGFLERNRAWILGIGTLGTIMSLVTMCVPL : 1575
FZ99       : EIMSELQVRQEVASFMNQSKVRRFSDPETLFSDLEDLKLEFDFDQLEQQAKLFAKPKEGKISKFRAWVKDCTGKIKGFLERNRAWILGIGTLGTIMSLVTMCVPL : 1575
             EIMSELQVRQEVASFMNQSkvRRfSDPETLFSDLEDLKLEFDFDQLEQQAKLFAKPKEGKISKFRAWV4DCTGKIKGFLERNRAWILGIGTLGTImSLVTMCVPL

               1580         *        1600         *        1620         *        1640         *        1660         *        1680
CH60       : ARRFAQSIYSQQPVAKTLPKDFKVAVKKHIEKLETGLEDQSGRVNFRHICNRLVNVSNEDEVATGLAIGGKNVLTFGHSKFTQLDEIRDMQFNAPAKGTPITYDG : 1680
A66        : ARRFAQSIYSQQPVAKTLPKDFKVAVKKHIEKLETGLEDQSGRVNFRHICNRLVNVSNEDEVATGLAIGGKNVLTFGHSKFTQLDEIRDMQFNAPAKGTPITYDG : 1680
C80        : ARRFAQSIYSQQPVAKTLPKDFKVAVKKHIEKLETGLEDQSGRVNFRHICNRLVNVSNEDEVATGLAIGGKNVLTFGHSKFTQLDEIRDMQFNAPAKGTPITYDG : 1680
MY         : ARRFAQSIYSQQPVAKTLPKDFKVAVKKHIEKLETGLEDQSGRVNFRHICNRLVNVSNEDEVATGLAIGGKNVLTFGHSKFTQLDEIRDMQFNAPAKGTPITYDG : 1680
FC64       : ARRFAQSIYSQQPVAKTLPKDFKVAVKKHIEKLETGLEDQSGRVNFRHICNRLVNVSNEDEVATGLAIGGKNVLTFGHSKFTQLDEIRDMQFNAPAKGTPITYDG : 1680
03D        : ARRFAQSIYSQQPVAKTLPKDFKVAVKKHIEKLETELEDQSGRVNFRHICNRLVNVSNEDEVATGLAIGGKNVLTFGHSKFTQLDEIRDIQFNAPAKGTPITYDG : 1680
ZJ         : ARRFAQSIYSQQPVAKTLPKDFKVAVKKHIDKLETELEDQSGRVNFRHICNRLVNVSNENEVATGLAIGGKNVLTFGHSKFTQLDEIRDMQFNAPAKGTPITYDG : 1680
NA         : ARRFAQSIYSQQPVAKTLPKDFKVAVKKHIDKLETELEDQSGRVNFRHICNRLVNVSNENEVATGLAIGGKNVLTFGHSKFTQLDEIRDMQFNAPAKGTPITYDG : 1680
C-XNH      : ARRFAQSIYSQQPVAKTLPKDFKVAVKKHIDKLETELEDQSGRVNFRHICNRLVNVSNENEVATGLAIGGKNVLTFGHSKFTQLDEIRDMQFNAPAKGTPITYDG : 1680
LSD/090830 : ARRFAQSIYSQQPVAKTLPKDFKVAVKKHIEKLETGLEDQSGRVNFRHICNRLVNVSNEDEVATGLAIGGKNVLTFGHSKFTQLDEIRDMQFNAPVKGTPITYDG : 1680
FZ99       : ARRFAQSIYSQQPVAKTLPKDFKVAVKKHIDKLETELEDQSGRVNFRHICNRLVNVSNENEVATGLAIGGKNVLTFGHSKFTQLDEIRDMQFNAPAKGTPITYDG : 1680
             ARRFAQSIYSQQPVAKTLPKDFKVAVKKHI KLET LEDQSGRVNFRHICNRLVNVSNE1EVATGLAIGGKNVLTFGHSKFTQLDEIRD6QFNAPaKGTPITYDG

                        *        1700         *        1720         *        1740         *        1760         *        1780
CH60       : EPTDLQLLECDIPHQFKDVSKLIATEDYRGNGWLVWKDQDQYMVQDVTKIRPFGSTTTASGTTSCQTYIYNCKTGPGSCGGVLVAQIGGNLKILGIHTSGNGTMG : 1785
A66        : EPTDLQLLECDIPHQFKDVSKLIATEDYRGNGWLVWKDQDQYMVQDVTKIRPFGSTTTASGTTSCQTYIYNCKTGPGSCGGVLVAQIGGNLKILGIHTSGNGTMG : 1785
C80        : EPTDLQLLECDIPHQFKDVSKLIATEDYRGNGWLVWKDQDQYMVQDVTKIRPFGSTTTASGTTSCQTYIYNCKTGPGSCGGVLVAQIGGNLKILGIHTSGNGTMG : 1785
MY         : EPTDLQLLECDIPHQFKDVSKLIATEDYRGNGWLVWKDQDQYMVQDVTKIRPFGSTTTASGTTSCQTYIYNCKTGPGSCGGVLVAQIGGNLKILGIHTSGNGTMG : 1785
FC64       : EPTDLQLLECDIPHQFKDVSKLIATEDYRGNGWLVWKDQDQYMVQDVTKIRPFGSTTTASGTTSCQTYIYNCKTGPGSCGGVLVAQIGGNLKILGIHTSGNGTMG : 1785
03D        : EPTDLQLLECDIPHQFKDVSKLIATEDYRGNGWLVWKDQDQYMVQDVTKIRPFGSTTTASGTTSCQTYIYNCKTGPGSCGGVLVAQIGGNLKILGIHTSGNGTMG : 1785
ZJ         : EPTDLQLLECDIPHQFKDVSKLIATEDYRGNGWLVWKDQDQYMVQDVTKIRPFGSTTTASGTTSCQTYIYNCKTGPGSCGGVLVAQIGGNLKILGIHTSGNGTMG : 1785
NA         : EPTDLQLLECDIPHQFKDVSKLIATEDYRGNGWLVWKDQDQYMVQDVTKIRPFGSTTTASGTTSCQTYIYNCKTGPGSCGGVLVAQIGGNLKILGIHTSGNGTMG : 1785
C-XNH      : EPTDLQLLECDIPHQFKDVSKLIATEDYRGNGWLVWKDQDQYMVQDVTKIRPFGSTTTASGTTSCQTYIYNCKTGPGSCGGVLVAQIGGNLKILGIHTSGNGTMG : 1785
LSD/090830 : EPTDLQLLECDIPHQFKDVSKLIATEDYRGNGWLVWKDQDQYMVQDVTKIRPFGSTTTASGTTSCQTYIYNCKTGPGSCGGVLVAQIGGNLKILGIHTSGNGTMG : 1785
FZ99       : EPTDLQLLECDIPHQFKDVSKLIATEDYRGNGWLVWKDQDQYMVQDVTKIRPFGSTTTASGTTSCQTYIYNCKTGPGSCGGVLVAQIGGNLKILGIHTSGNGTMG : 1785
             EPTDLQLLECDIPHQFKDVSKLIATEDYRGNGWLVWKDQDQYMVQDVTKIRPFGSTTTASGTTSCQTYIYNCKTGPGSCGGVLVAQIGGNLKILGIHTSGNGTMG

                  *        1800         *        1820         *        1840         *        1860         *        1880         *
CH60       : AANRVFPVFNQGKVVSKQYAGKILYHQPRKTAYMKSPVYEDSPYEPAVLSINDQRLEVKIEDMAKKASDKYIGNVFQPPPEAFQLAKTHVAEKLSRVLGSHETIS : 1890
A66        : AANRVFPVFNQGKVVSKLYAGKILYHQPRKTAYMKSPVYEDSPYEPAVLSINDQRLEVKIEDMAKKASDKYIGNVFQPPPEAFQLAKTHVAEKLSRVLGSHETIS : 1890
C80        : AANRVFPVFNQGKVVSKQYAGKILYHQPRKTAYMKSPVYEDSPYEPAVLSINDQRLEVKIEDMAKKASDKYIGNVFQPPPEAFQLAKTHVAEKLSRVLGSHETIS : 1890
MY         : AANRVFPVFNQGKVVSKQYAGKILYHQPRKTAYMKSPVYEDSPYEPAVLSINDQRLEVKIEDMAKKASDKYIGNVFQPPPEAFQLAKTHVAEKLSRVLGSHETIS : 1890
FC64       : AANRVFPVFNQGKVVSKQYAGKILYHQPRKTAYMKSPVYEDSPYEPAVLSINDQRLEVKIEDMAKKASDKYIGNVFQPPPEAFQLAKTHVAEKLSRVLGSHETIS : 1890
03D        : AANRVFPVFNQGKVVSKQYAGKILYHQPRKTAYMKSPVYEDSPYEPAVLSINDQRLGVKIEDMAKKASDKYIGNVFQPPPEAFQLAKTHVAEKLSRVLGCHDTIS : 1890
ZJ         : AANRVFPVFNQGKVVSKQYAGKILYHQPRKTAYMKSPVYEDSPYEPAVLSINDQRLGVKIEDMAKKASDKYIGNVFQPPPEAFQLAKTHVAEKLSRVLGCHDIIS : 1890
NA         : AANRVFPVFNQGKVVSKQYAGKILYHQPRKTAYMKSPVYEDSPYEPAVLSINDQRLGVKIEDMAKKASDKYIGNVFQPPPEAFQLAKTHVAEKLSRVLGCHDIIS : 1890
C-XNH      : AANRVFPVFNQGKVVSKQYAGKILYHQPRKTAYMKSPVYEDSPYEPAVLSINDQRLGVKIEDMAKKASDKYIGNVFQPPPEAFQLAKTHVAEKLSRVLGCHDIIS : 1890
LSD/090830 : AANRVFPVFNQGKVVSKQYAGKILYHQPRKTAYMKSPVYEDSPYEPAVLSINDQRLGVKIEDMAKKASDKYIGNVFQPPPEAFQLAKTHVAEKLSRVLGCHDTIS : 1890
FZ99       : AANRVFPVFNQGKVVSKQYAGKILYHQPRKTAYMKSPVYEDSPYEPAVLSINDQRLGVKIEDMAKKASDKYIGNVFQPPPEAFQLAKTHVAEKLSRVLGCHDIIS : 1890
             AANRVFPVFNQGKVVSKqYAGKILYHQPRKTAYMKSPVYEDSPYEPAVLSINDQRL VKIEDMAKKASDKYIGNVFQPPPEAFQLAKTHVAEKLSRVLG H  IS

                    1900         *        1920         *        1940         *        1960         *        1980         *
CH60       : YESAVSSDVIPMNWDTSPGIKYKGETKRQLVLKSSFRQDVMEQLQSPSTVFTCYLKDELRKKEKIKEGKTRGIEACNFDHTVAYRMVMGDIFSNIYDDSFIMSGC : 1995
A66        : YESAVSSDVIPMNWDTSPGIKYKGETKRQLVLKSSFRQDVMEQLQSPSTVFTCYLKDELRKKEKIKEGKTRGIEACNFDHTVAYRMVMGDIFSNIYDDSFIMSGC : 1995
C80        : YESAVSSDVIPMNWDTSPGIKYKGETKRQLVLKSSFRQDVMEQLQSPSTVFTCYLKDELRKKEKIKEGKTRGIEACNFDHTVAYRMVMGDIFSNIYDDSFIMSGC : 1995
MY         : YESAVSSDVIPMNWDTSPGIKYKGETKRQLVLKSSFRQDVMEQLQSPSTVFTCYLKDELRKKEKVKEGKTRGIEACNFDHTVAYRMVMGDIFSNIYDDSFIMSGC : 1995
FC64       : YESAVSSDVIPMNWDTSPGIKYKGETKRQLVLKSSFRQDVMEQLQSPSTVFTCYLKDELRKKEKIKEGKTRGIEACNFDHTVAYRMVMGDIFSNIYDDSFIMSGC : 1995
03D        : YESAVSSDVIPMNWDTSPGIKYKGETKRQLVLKSSFRQDVLKQLQSPSTVFTCYLKDELRKKEKIKEGKTRGIEACNFDHTVAYRMVMGDIFSNIYDDSFIMSGC : 1995
ZJ         : YESAVSSDVIPMNWDTSPGIKYKGETKRQLVLKSSFRQDVLEQLQSPSTVFTCYLKDELRKKEKIKEGKTRGIEACNFDHTVAYRMVMGDIFSNIYDDSFIMSGC : 1995
NA         : YESAVSSDVIPMNWDTSPGIKYKGETKRQLVLKSSFRQDVLEQLQSPSTVFTCYLKDELRKKEKIKEGKTRGIEACNFDHTVAYRMVMGDIFSNIYDDSFIMSGC : 1995
C-XNH      : YESAVSSDVIPMNWDTSPGIKYKGETKRQLVLKSSFRQDVLEQLQSPSTVFTCYLKDELRKKEKIKEGKTRGIEACNFDHTVAYRMVMGDIFSNIYDDSFIVSGC : 1995
LSD/090830 : YESAVSSDVIPMNWDTSPGIKYKGETKRQLVLKSSFRQDVLEQLQSPSTVFTCYLKDELRKKEKIKEGKTRGIEACNFDHTVAYRMVMGDIFSNIYDDSFIMSGC : 1995
FZ99       : YESAVSSDVIPMNWDTSPGIKYKGETKRQLVLKSSFRQDVLEQLQSPSTVFTCYLKDELRKKEKIKEGKTRGIEACNFDHTVAYRMVMGDIFSNIYDDSFIMSGC : 1995
             YESAVSSDVIPMNWDTSPGIKYKGETKRQLVLKSSFRQDV6eQLQSPSTVFTCYLKDELRKKEK6KEGKTRGIEACNFDHTVAYRMVMGDIFSNIYDDSFI6SGC

               2000         *        2020         *        2040         *        2060         *        2080         *        2100
CH60       : AVGINPFCEWDNLLANLQPYNLCLDFSGFDGSLSAQILEEAVDVLSYFHNDPALVKRIHAPTIYSTHYVTDEIWQVEGGMCSGSPCTTVVNSIVNQLACYTILAV : 2100
A66        : AVGINPFCEWDNLLANLQPYNLCLDFSGFDGSLSAQILEEAVDVLSYFHNDPALVKRIHAPTIYSTHYVTDEIWQVEGGMCSGSPCTTVVNSIVNQLACYTILAV : 2100
C80        : AVGINPFCEWDNLLANLQPYNLCLDFSGFDGSLSAQILEEAVDVLSYFHNDPALVKRIHAPTIYSTHYVTDEIWQVEGGMCSGSPCTTVVNSIVNQLACYTILAV : 2100
MY         : AVGINPFCEWDNLLANLQPYNLCLDFSGFDGSLSAQILEEAVDVLSYFHSDPALVKRIHAPTIYSTHYVTDEIWQVEGGMCSGSPCTTVVNSIVNQLACYTILAV : 2100
FC64       : AVGINPFCEWDNLLANLQPYNLCLDFSGFDGSLSAQILEEAVDVLSYFHNDPALVKRIHAPTIYSTHYVTDEIWQVEGGMCSGSPCTTVVNSIVNQLACYTILAV : 2100
03D        : AVGINPFCEWDNLLANLQPYNLCLDFSGFDGSLSAQILEEAVDVLSYFHNDPALVKRIHAPTIYSTHYVTDEIWQVEGGMCSGSPCTTVVNSIVNQLACYTILAV : 2100
ZJ         : AVGINPFCEWDNLLANLQPYNLCLDFSGFDGSLSAQILEEAVDVLSYFHNDPALVKRIHAPTIYSTHYVTDEIWQVEGGMCSGSPCTTVVNSIVNQLACYTILAV : 2100
NA         : AVGINPSCEWDNLLANLQPYNLCLDFSGFDGSLSAQILEEAVDVLSYFHNDPALVKRIHAPTIYSTHYVTDEIWQVEGGMCSGSPCTTVVNSIVNQLACYTILAV : 2100
C-XNH      : AVGINPFCEWDNLLANLQPYNLCLDFSGFDGSLSAQILEEAVDILSYFHNDPALVKRIHAPTIYSTHYVTDEIWQVEGGMCSGSPCTTVINSIVNQLACYTILAV : 2100
LSD/090830 : AVGINPFCEWDNLLANLQPYNLCLDFSGFDGSLSVQILEEAVDVLSYFHNDPALVKRIHAPTIYSTHYVTDEIWQVEGGMCSGSPCTTVVNSIVNQLACYTILAV : 2100
FZ99       : AVGINPFCEWDNLLANLQPYNLCLDFSGFDGSLSAQILEEAVDVLSYFHNDPALVKRIHAPTIYSTHYVTDEIWQVEGGMCSGSPCTTVVNSIVNQLACYTILAV : 2100
             AVGINPfCEWDNLLANLQPYNLCLDFSGFDGSLSaQILEEAVD6LSYFHnDPALVKRIHAPTIYSTHYVTDEIWQVEGGMCSGSPCTTV6NSIVNQLACYTILAV

                        *        2120         *        2140         *        2160         *        2180         *        2200
CH60       : LGYDINQCYVVSYGDDCVLSVPEVRDISKLSHYFKLFFGMTATASDKESDITWLAPMEIEFLKRTPAFLPGTRKIIGVLDKEVLEGKIQWCKGPEAFKQQLDSFL : 2205
A66        : LGYDINQCYVVSYGDDCVLSVPEVRDISKLSHYFKLFFGMTATASDKESDITWLAPMEIEFLKRTPAFLPGTRKIIGVLDKEVLEGKIQWCKGPEAFKQQLDSFL : 2205
C80        : LGYDINQCYVVSYGDDCVLSVPEVRDISKLSHYFKLFFGMTATASDKESDITWLAPMEIEFLKRTPAFLPGTRKIIGVLDKEVLEGKIQWCKGPEAFKQQLDSFL : 2205
MY         : LGYDINQCYVVSYGDDCVLSVPEVRDISKLSHYFKLFFGMTATASDKESDITWLAPMEIEFLKRTPAFLPGTRKIIGVLDKEVLEGKIQWCKGPEAFKQQLDSFL : 2205
FC64       : LGYDINQCYVVSYGDDCVLSVPEVRDISKLSHYFKLFFGMTATASDKESDITWLAPMEIEFLKRTPAFLPGTRKIIGVLDKEVLEGKIQWCKGPEAFKQQLDSFL : 2205
03D        : LGYDINQCYVVSYGDDCVLSVPEVRDISKLSHYFKLFFGMTATASDKESDITWLAPMEIEFLKRTPAFLPGTRKIIGVLDKEVLEGKIQWCKGPEAFKQQLDSFL : 2205
ZJ         : LGYDINQCYVVSYGDDCVLSVPEVRDISKLSHYFKLFFGMTATASDKESDITWLAPMEIEFLKRTPAFLPGTRKIIGVLDKEVLEGKIQWCKGPEAFKQQLDSFL : 2205
NA         : LGYDINQCYVVSYGDDCVLSVPEVRDISKLSHYFKLFFGMTATASDKESDITWLAPMEIEFLKRTPAFLPGTRKIIGVLDKEVLEGKIQWCKGPEAFKQQLDSFL : 2205
C-XNH      : LGYDINQCYVVSYGDDCVLSVPEVRDISKLSHYFKLFFGMTATASDKESDITWLAPMEIEFLKRTPAFLPGTRKIIGVLDKEVLEGKIQWCKGPEAFKQQLDSFL : 2205
LSD/090830 : LGYDINQCYVVSYGDDCVLSVPEVRDISKLSHYFKLFFGMTATASDKESDITWLAPMEIEFLKRTPAFLPGTRKIIGVLDKEVLEGKIQWCKGPEAFKQQLDSFL : 2205
FZ99       : LGYDINQCYVVSYGDDCVLSVPEVRDISKLSHYFKLFFGMTATASDKESDITWLAPMEIEFLKRTPAFLPGTRKIIGVLDKEVLEGKIQWCKGPEAFKQQLDSFL : 2205
             LGYDINQCYVVSYGDDCVLSVPEVRDISKLSHYFKLFFGMTATASDKESDITWLAPMEIEFLKRTPAFLPGTRKIIGVLDKEVLEGKIQWCKGPEAFKQQLDSFL

                  *        2220         *        2240         *
CH60       : LEAALHGEEYYHEVTSKLKARCPVFDIQPWGVAKLRAYTACMMI* : 2249
A66        : LEAALHGEEYYHEVTSKLKARCPVFDIQPWGVAKLRAYTACMMI* : 2249
C80        : LEAALHGEEYYHEVTSKLKARCPVFDIQPWGVAKLRAYTACMMI* : 2249
MY         : LEAALHGEEYYHEVTSKLKARCPVFDIQPWGVAKLRAYTACMMI* : 2249
FC64       : LEAALHGEEYYHEVTSKLKARCPVFDIQPWGVAKLRAYTACMMI* : 2249
03D        : LEAALHGEEYYHEVTSKLKARCPVLDIQPWGVAKLRAYTACMMI* : 2249
ZJ         : LEAALHGEEYYHEVTSKLKARCPVLDIQPWGVAKLRAYTACMMI* : 2249
```

```
NA         : LEAALHGEEYYHEVTSKLKARCPVLDIQPWGVAKLRAYTACMMI* : 2249
C-XNH      : LEAALHGEEYYHEVTSKLKARCPVLDIQPWGVAKLRAYTACMMI* : 2249
LSD/090830 : LEAALHGEEYYHEVTSKLKARCPVLDIQPWGVAKLRAYTACMMI* : 2249
FZ99       : LEAALHGEEYYHEVTSKLKTRCPVLDIQPWGVAKLRAYTACMMI* : 2249
             LEAALHGEEYYHEVTSKLKaRCPV DIQPWGVAKLRAYTACMMI
```

Supplementary data.3 Constructed viral ORFs (nucleotides and protein) between virulent and attenuated strains

> Conserved attenuated ORF

ATGGATACTCTTACCAAAAATATTGAAGATGCAACAGTCAACATCATTGGATCTTGTGCAGAAAAGGTG
GAGGAAGCAATTTCAGGCCTAGGGGCAGTGGAAAGTGTGGCATCCACTAATTCAGCCATTGCCACT
GCCAATGCAACAACTACACAGACAATACTAGACCCAACGGAGGGTTCTACTGATGATTTCTACTCCTG
TTCTTATGAAGTAGGAGCTCAAGGGGATAATATTTCTAGATTGGTACATCTGGTTACAGGACAGTGGG
TTCCAAATGATGATTATTATGCCTGCCTACGCTGGTTAGCAACACCTGCTTGTTTTTCCAAAATAACA
CACAACCAGCATATGGCCAGACACGATATTTTAGGTTTATCAGATGTGGCTTCCATTTTAGGTTGCTTG
TGAATGCCCCCTCTGGCTCTGCTGGAGCACTTATGCTAGTTTGGATGCCCTACCCCTATTGTCGGGT
CTTATCTGGTGCTAATCAGATCCATACAAATGTTGAGAGAAGGAGTCTAATGAACCTGCCCTATGCCAT
CTTGGATCTCCGCACCAACACAGAAATTGACCTTGTAGTTCCATATGTCAACTACCGGAACTATGTAG
AGATTACAACCAGTGATACAACTGGTGGTGCCATCTGTGTCATTGTGTTAGGCAAGTACCGACATGG
CAATGGAACCTCCAACACTGTAGATTTCACATTATTTGGAGAACTCCTTGAAACTGATTTGCAGTGTC
CCCGACCATTTGACAATCAGGGAAAGAGAAAACCATGCAGGAGGCCCATTCACAAACCCAAGAATC
CACCACAAGAGCCAAGGGTCATCATTCAACCAGGACCTGGGGCAGCAAACCTTTCAAATTCTAGTGT
TATAGCAATGGCTGAAAGCACAGCACTTGCTAATGAAGGGACAGCTGTTGACTATTCCACAGCTGGA
TGTGCATCATCTGTTGATGATGTAATTATGGTCCTGCGCCGATGGCAGATTCTAGCAAGTTTCCAGTG
GCAGTCAACCCTAACTCCCAGTGCTCGCATTAATCGCTATCAATTGATTTTCAGAAACATCCCAACATT
TTCTCTTTTTTTTGACAAATTTCAGTACTGGAGAGGGTCATTGGAGGTTAAATTTATGACATTTGGCAG
CCAATTTAACACTGGGAGATATCAAATGTCTTGGTATCCAATTGCTGATGGTGAACAGAGCTTGGCAC
AATGCCAGAATTCGGTATTTGTAACTGGGGATGTGTGTGCCACACCTGTGACCCTAACTCTGCCATTT
ACATCAACCACCTGGCGCAAAAGCACGAGAGACCCTTACGGGTATTTGATGTGGCATGTTGTCAATC
GACTCACTGTGAATTCATCAGCCCCATCCAATATTGACTGCACAGTGTTGCTCCGCGTGGGCAAAGA
TTTTCAGTTTACCGCCCCACTCTATGGAAATTTGCAGATGGCAACTAACAATCAAGGTGATACCAACC
AGCTAGGGGATGATGAGCCAGTTTGCTTCCTGAATTTTGAGACAGCTAATGTCCCAATACAAGGTGAA
TCTCACACTCTTGTTAAACACCTGTTTGGGAGGCAATGGTTAGTTAGGACTGTGCAACATGCTTCAAC
TGTGCAAGAGTTGGACCTCCAAGTCCCAGATAGAGGACATGCCTCTCTCATTCGGTTCTTTGCCTATT
TTTCTGGAGAGATCATTCTTACCATTGTTAACAACGGCACTACACCAGCGATGGTGGCACATTCCTAT
TCTATGGATGACCTCAGTTCAGAGTATGCTGTTACAGCAATGGGAGGTGTGATGATCCCTGCCAATAG
TGCCAAAAATATCTCTGTACCATTCTACTCTGTGACACCACTCAGGCCAACTCGACCAATTCCTGGCA
CATCAGAGGCAACTTTTGGCAGGCTGTTCATGTGGACTCAATCAGGAAGTCTTTCAGTTTTTATGGGT
CTCAAAAAGCCAGCTCTCTTCTTTCCACTCCCTGCTCCCACTTCCACAACACTATCACGGGGATCCA
ATGGTGTTATTCCCACATTGGATCAGTCTGGAGATGAAGTAGATTGTCACTTCTGCAAAATTTGTTCTA
AAATGAAGAGGATGTGGAAGCCAAAAGGGCACTTCAGATTTTGCCTTAGACTCAAAACACTAGCATTT
GAACTCGATCTGGAAATTGAATCTGACCAAATTAGAAACAAGAAAGATCTCACTACTGAAGGAGTGGA
ACCGAACCCAGGACCTATCTTAGTTGTTGGCAAATCAGGAAGTGGCAAGAGCCGACTTTGCAACGTT
CTAGCTGGTGTGGAGCTTTTTGAATCAAAACTGTCACCACGTTCAGTTACAATGGTTCATCAGGTTGA
GACAATTGACATTGAAGGGAAGAAGGTGACAATAGTGGATTCACCCGAAACACCAAATTATGATGGC
CCCATTTCTGCCTTTTTTTACTTGATTGAGGCAGGCAGGTTCACAGCTGAAGACAAAGAGTACATCCA
AATGATGAGAAAGCGCTTTCCAAGCTTTGAGAAATCCACTATTTTGATTTTGAACCGAGCAGATGAATT

GAAAACTGATGATGATATAAGAAATTGGATTAAGACATCTGGAGAGTTGGACTCTTTAGTGAGAGCCT
GTGGGGGGCGTATAGCTAAATTTCACCGGTATAAAATAAACAGGGCTAAGTTGTTAGAGAAAGCAGCT
ACCCTTCCTGAGTTTGTCTCTCATTTGCCAAGACTTGTCTACAAGGATAGAAAAATGTACCGCCATTAT
GGTGTGCAGTGTGGTTCCACAGTTTTCCATATGGACTCAGAGAACATCCTTGAGTCAGCATTAAATG
GTGAAGTCACAATCAAGCAAGAGAAGTGGAATGGCAATTGGAAACCAGCAGGTGATCACATGCAGT
CAACAGCTAGCATGTATCTTAAATCACAGACTATGCCAAAATTCACTTTTTCAATTGATGAAAACTGTG
AAACCTGGGCTAGACACTTGTTGGGCGACTATGGGGAAACACAAGGTCAGATTTTTAAGGAAAGGCT
TATGTGGGCTGTAGCCCTTGGTTTTTTTATGACAATGAAAATCACTACAGACCAATCATTTCCAGGTAA
AGATGCAATACACACAGTTCTCACCAAAATTTCAAATTTTATTTTTGGGGGCTTGGAAAATGAAGTTGT
CCGTGTTGTCATCAGGACTGTGATTCGCATTGTTTGTTATCTCATTTTGTATATACATTCTCCCAACATA
GTCACAACAGGGACTTTGGTTGCTTTGCTAGCTTTAGATGCCACCAGCATGTCAATGGATCAAGGAC
TCAAAACTCTCTGCATGAGCCTAGTGGATGGTGATTTTGGCAAATTTTGTTCAGCTTTGTTAGAAAAA
ATACAAACTGTTGACGAGGCTGATCTAAAGAAAACCATTCCAAAATTCAATGACATGTTAGAGGATCAA
TCTGGCAAAACCACCTCTCCTAAATCTTTTAATGATTGGACAACCTGTGCCAAAAATGTACAATGGTG
GCTTGAATCATTTGTCAAAGTTGTAAATTGGCTTAAAGAAAAAATATTTCCTTCAAAAACTGACCCAAC
TCTTCAGTGGCTCCAGGACCACGAAGAACATATAGCTATCATGTTGGCATTGTGTGATGAGCACCTTT
GTATGCTAAGAACTGAAAAAGATTACATTTGTGAGCACAATACTCGCCCAAAACATCAGTGTTTGGTT
GAAATGGTCTCTGGCACATTGAACCAACTCCAAGGTATTTCAAGTGCTCGTGAATTGGCAGCTAGAC
TACAGCATGTGTTGAACAAACTCCACCAAGTCAATTTTGAGCCTGAACTGGAGTGGACACACCGTCC
TGAACCTCTTGGTATATGGATATCTGGTGGCCCTGGTGTTGGGAAGAGCTTTTTATCAAATTACATTGT
TAAAGAAATAGCCAAATTAAAGCATTGGAAGAGTTATGCCAACCCAACTGGGAGTAAGCATATGGATG
GGTATGTGTCACAGGAGATTCATGTTTTTGATGATTTTGGTCAAAATCGGGAAGAGGAAGACTATTCT
TTAATTTGTAACCTTATTTCTAGTGTTCCATTCATTACTCCAAAGGCAAGTGTAGAGGCCAAGGGCACC
CAGTACAGGGGACGCCTTGTTGTTGTCACAACCAATAGACGGGATTTCACTTCTTGCAAGTTGACTG
ACCCAGATGCATTGGAAAGGCGTTTCCCTATTCGCCTTAATATTCGGCCATTGCAAAAATATAACCACA
AGGGCCGTTTGGATGTGGCAACAGCTATGAGAGATGGAAGCTTGCAGGGAGGAGCTTGCTGGGAA
AGGGATATAGGCCAATTAGGCCTTGAATGTTGGAATCCTATAAATGGCCAAACCCTGATTGATGAAATT
ATGAGTGAGTTGCAGGTTAGGCAAGAAGTAGCTTCCTTTATGAACCAGTCTAAGGTGAGGCGTTTCT
CTGACCCGGAGACTCTTTTTAGTGATTTGGAAGATCTAAAATTGGAATTTGATTTTGATCAACTCGAGC
AACAAGCTAAACTTTTTGCAAAACCAAAAGAAGGAAAAATCTCTAAATTTAGGGCTTGGGTTAGAGAC
TGCACAGGGAAGATTAAGGGTTTTCTGGAGCGGAATCGGGCTTGGATTCTTGGTATAGGAACCCTTG
GAACCATCATGTCACTTGTGACCATGTGTGTCCCATTAGCTAGACGCTTTGCTCAGTCAATCTATTCA
CAACAACCTGTGGCCAAAACACTCCCTAAAGACTTTAAGGTAGCTGTTAAGAAACACATTGAGAAATT
AGAAACTGGATTGGAAGACCAGAGCGGGCGGGTGAATTTCAGACATATTTGCAATAGACTCGTCAAT
GTGAGTAATGAGGATGAAGTAGCCACTGGTTTAGCCATTGGTGGGAAAAATGTCTTGACATTTGGGC
ATTCTAAATTTACACAATTGGATGAGATTAGGGACATGCAATTCAATGCACCAGCTAAGGGGACCCCC
ATTACTTATGATGGAGAACCCACTGATCTCCAGTTATTGGAATGTGATATACCCCATCAGTTCAAAGAT
GTGTCTAAATTGATTGCCACGGAGGACTATCGCGGTAATGGTTGGTTGGTTTGGAAAGATCAGGACC
AGTATATGGTGCAAGATGTGACAAAGATTAGGCCTTTTGGTTCCACAACAACTGCATCAGGCACAACA
TCATGTCAAACATATATATATATAATTGCAAGACTGGGCCTGGCTCCTGTGGTGGTGTGCTTGTAGCTCA
AATTGGAGGAAATTTGAAGATCCTTGGGATACATACAAGTGGCAATGGCACTATGGGAGCAGCCAATA
GGGTCTTTCCAGTTTTTAATCAAGGGAAAGTAGTGAGCAAGCAATATGCAGGTAAGATTCTGTACCAT
CAACCCCGAAAAACTGCATATATGAAGTCACCTGTCTATGAGGACTCACCTTATGAGCCTGCAGTTCT

TTCCATCAATGACCAGCGACTTGAGGTGAAAATTGAAGACATGGCTAAGAAAGCATCTGACAAGTATA
TTGGCAATGTGTTTCAGCCTCCTCCTGAGGCTTTTCAACTTGCAAAGACACATGTTGCCGAAAAACT
GTCCAGAGTCTTGGGAAGCCATGAAACTATCTCATATGAGTCAGCTGTGAGTAGTGATGTTATACCTA
TGAACTGGGATACTTCACCTGGCATTAAGTATAAGGGGGAGACCAAACGACAACTGGTGCTGAAGTC
CTCATTCAGACAGGATGTCATGGAGCAACTACAGAGTCCATCCACTGTTTTCACTTGTTATCTGAAGG
ATGAACTCCGAAAGAAGGAAAAAATCAAGGAAGGGAAAACAAGAGGCATTGAGGCTTGTAATTTTGA
TCACACCGTGGCTTATAGAATGGTGATGGGTGATATTTTTTCTAACATCTATGATGATTCTTTTATTATGT
CTGGCTGTGCTGTTGGAATTAATCCTTTTTGTGAGTGGGACAATCTGTTGGCAAACCTCCAACCTTAT
AATTTGTGTCTTGACTTTTCTGGATTCGATGGCTCTCTGAGTGCCCAAATTCTTGAAGAAGCAGTGGA
TGTCTTGTCTTATTTCCACAATGACCCAGCCTTAGTGAAAAGAATTCATGCACCAACTATCTATTCAAC
TCATTATGTAACTGATGAGATATGGCAGGTAGAAGGAGGGATGTGTTCAGGATCCCCATGTACTACCG
TGGTCAATTCTATTGTGAATCAGCTTGCGTGCTACACCATTTTAGCTGTATTGGGCTATGACATCAACC
AATGTTATGTCGTTAGTTATGGGGATGACTGTGTTCTGTCAGTGCCAGAGGTGCGCGATATCTCAAAA
CTATCGCACTATTTCAAGCTTTTCTTTGGTATGACAGCAACAGCTAGTGACAAAGAAAGTGACATAACT
TGGCTAGCCCCTATGGAGATAGAATTTCTGAAGAGAACTCCTGCCTTCCTCCCAGGGACTCGCAAAA
TCATTGGTGTCCTTGATAAGGAAGTTTTGGAAGGGAAGATACAGTGGTGCAAGGGACCAGAAGCATT
CAAACAGCAACTTGACTCTTTCTTGCTGGAAGCAGCTCTGCATGGAGAAGAATACTACCATGAGGTG
ACATCTAAGCTTAAGGCCCGGTGCCCCGTTTTTGACATTCAACCTTGGGGTGTCGCGAAACTCAGAG
CATACACAGCTTGCATGATGATCTGA

>Conserved virulent ORF

ATGGATACTCTTACCAAAAACATTGAAGATGCAACAGTCAACATCATTGGATCTTGTGCAGAAAAGGT
GGAGGAAGCAATTTCAGGCCTAGGGGCAGTGGAAAGTGTGGCATCCACCAACTCAGCCATTGCCAC
TGCCAATGCAACAACTACACAGACAATACCAGACCCAACGGAGGGTTCCACTGATGATTTCTACTCTT
GTTCTTATGAAGTAGGAGCTCAAGGGGATAACATTTCTAGATTGGTACATCTGGTTACAGGACAGTGG
GTTCCAAATGATGATTATTATGCCTGCCTACGCTGGTTAGCAACACCTGCTTGTTTTTTTCAAAATAAC
ACACAACCAGCATATGGCCAGACACGATATTTTAGGTTTATTAGATGTGGCTTCCATTTTAGGTTGCTT
GTGAATGCCCCCTCTGGCTCTGCTGGAGCGCTTATGCTAGTTTGGATGCCCTACCCCTATTGTCGGG
TCTTATCTGGTACTAATCAGATCCATGCAAATGTTGAGAGAAGGAGTCTAATGAACCTGCCCTATGCC
ATCTTGGATCTCCGCACCAACACAGAAATTGACCTTGTGGTTCCATATGTCAACTACCGGAACTATGT
GGAGATTACAACCAGTGACACAACTGGTGGTGCCATCTGTGTGATTGTGTTAGGCAAGTACCGACAT
GGCAATGGAACCTCCAACACTGTGGATTTCACATTATTTGGAGAACTCCTTGAAACTGATTTGCAGTG
CCCCCGACCATTTGACAATCAGGGAAAGAGAAAACCACGCAGGAGGCCCATTCACAAACCCAAGAA
TCCACCACAGGAGCCAAGGGTTATTATTCAACCAGGACCTGGGGCAGCAAACCTTTCAAACTCCAGT
GTTATAGCAATGGCTGAAAGCACAGCACTTGCTAATGAAGGGACAGCTGTTGACTATTCCACAGCTG
GATGTGCATCATCTGTTGATGATGTAATTATGGTCCTGCGCCGCTGGCAGATTTTAGCAAGTTTCCAG
TGGCAGTCAACCCTAACTCCCAGTGCTCGCATTAATCGCTATCAATTGATTTTTAGAAATATCCCAACA
TTCTCTCTCTTTTTTGACAAATTTCAGTACTGGAGAGGGTCATTGGAGGTTAAATTCATGACATTTGGC
AGCCAATTTAACACTGGGAGATATCAAATGTCTTGGTATCCAATTGCTGATGGTGAACAAAGCTTGGC
ACAATGCCAGAATTCAGTGTTTGTAACTGGGGATGTGTGTGCCACACCCGTGACCCTAACTCTGCCA
TTTACATCAACCACCTGGCGCAAGAGCACGAGAGACCCTTACGGGTACTTGATGTGGCATGTTGTCA
ATCGACTCACTGTGAATTCATCAGCCCCATCCAATATTGACTGCACAGTGTTGCTCCGCGTGGGCAA
AGATTTTCAGTTTACTGCCCCACTCTATGGAAATTTGCAGATGGCAACCAATAATCAGGGTGATTCCA

ACCAGTTGGGGGATGATGAGCCAGTTTGCTTTCTGAATTTTGAGACAGCTAATGTCCCAATACAAGGT
GAATCTCACACTCTTGTTAAACACCTGTTTGGGAGGCAATGGTTAGTTAGGACTGTGCAACACGCTT
CAACTGTGCAAGAGTTGGACCTCCAAGTTCCAGACAGAGGACATGCCTCTCTCATTCGGTTCTTTGC
CTATTTTTCTGGAGAGATCATTCTTACCATTGTTAACAATGGCACTACACCAGCAATGGTAGCACACTC
CTATTCTATGGATGACCTCAGTTCAGAGTATGCTGTTACAGCAATGGGAGGTGTGATGATTCCTGCTA
ACAGTGCCAAAAATATTTCTGTACCATTCTACTCTGTGACACCACTCAGGCCAACTCGACCAATTCCT
GGCACATCAGAGGCAACTTTTGGCAGACTGTTCATGTGGACTCAATCAGGAAGTCTTTCAGTTTTTAT
GGGTCTCAAAAAGCCAGCTCTCTTCTTTCCACTCCCTGCTCCCACCTCCACAACACTATCACGGAAA
TCCAATGATGTTATTCCCACATTGAATCAGTCTGGGGATGAAGTAGATTGTCACTTCTGCGAAATTTGT
TCTAAAATGAAGAGGAGGTGGAAGCCAAGAGGGTACTTCAGATTTTGCCTTAGACTCAAAACACTAG
CATTTGAACTCAATCTGGAAATTGAATCTGACCAAATTAGAAATAAGAAAGATCTCACTACTGAAGGAG
TGGAACCAAACCCGGGACCTATCTTAGTTGTTGGCAAATCAGGAAGTGGCAAGAGCCGACTTTGTAA
TGTTTTAGCTGGTGTCGAGCTTTTTGAATCAAAACTGTCACCACGTTCAGTTACAATGGTTCATCAGG
TTGAGACAATTGACATTGAGGGGAAGAAGGTGACAATAGTGGATTCACCTGAAACACCAAATTATGAT
GGCCCCATTTCTGCCTTTTTTTATTTGATTGAGGCAGGCAGGTTCACAGCTGAAGACAAGGAATACAT
CCAAATGATGAGAAAACGCTTTCCAGGCTTTGAGAAATCCACTATTTTGATTCTGAACCGAGCAGATG
AATTGAAAACTGATGATGATATAAGAAATTGGGTTAAGACATCTGGAGAGTTGGACTCTTTAGTGAGA
GCCTGTGGGGGGCGTATAGCTAAATTTCATTGGCACAAAATAAACAGGGCTAAGTTGTTGGAGAAAG
CAGCCACCCTTCCTGAGTTTGTTTCTCATTTGCCAAGACTTGTCTATAAGGATAGAAAAATGTACCGC
CATTATGGTGTGCAGTGTGGTTCCACAGTCTTCCACATGGACTCAGAGAACATCCTTGAGTCAGCAT
TAAATGGTGAAGTCACAATCAAGCAGGAGAAGTGGAATGGCAATTGGAAACCAGCAGGTGATCACAT
GCAGTCAACAGCCAGCATGTATCTTAAATCACAGACTATGCCAAAATTCACTTTTTCAATTGATGAAAA
CTGTGAAACCTGGGCTAGACACTTGTTGGGTGACTATGGGGAAACACAAGGTCAGATTTTTAAGGAG
AGGCTTATGTGGGCTGCAGCCCTTGGGTTTTTTATGACAATGAAAATTACTACAGACCAATCATTTCCA
GGTAAAGATGCAATACACACAGTTCTTACCAAAATTTCAAATTTTATTTTTGGGGGTTTGGAAAATGAA
GTTGTCCGTGTTGTCATTAGGACTGTGATTCGCATTGTCTGTTATCTTATTTTGTATATACATTCTCCCA
ACATAGTCACAACAGGGACTTTGGTTGCTTTGTTAGCTTTAGATGCCACCAGCATGTCAATGGATCAA
GGACTCAAAACTCTCTGCATGAGCCTGGTGGATGGTGATTTTGGCAAATTTTGTTCAGCTTTGTTAGA
AAAAATACAAACTGTTGACGAGGCTGATCTAAAGAAAACTATTCCAAAATTCAATGACATGTTAGAGGA
TCAATCTGGCAAAACCACCTCTCCTAAGTCTTTTAATGATTGGACAACCTGTGCCAAAAATGTACAATG
GTGGCTTGAATCATTTGTTAAAGTTGTAAATTGGCTTAAAGAAAAAGTGTTTCCTTCAAAAACTGACCC
AACTCTTCAGTGGCTCCAGGATCATGAAGAACATATAGCTATTATGTTGGCATTGTGTGATGAGCACC
TTTGTATGCTAAGAACTGAAAAAGATTACATTTGTGAGCACAACACTCGTCCAAAACATCAGCGTTTG
GTTGAAATGGTCTCTGGCACATTGAACCAACTCCAAGGTATTTCAAGTGCTCGTGAATTGGCAGCCA
GACTACAGCATGTGTTGAACAAACTCCACCAAGTCAATTTTGAGCCTGAACTGGAGTGGACACACCG
TCCTGAACCTCTTGGTATATGGATATCTGGTGGCCCTGGTGTTGGGAAGAGCTTTTTGTCAAATTATAT
TGTTAAAGAAATAGCCAAATTAAAGCATTGGAAGAGTTATGCCAACCCAACTGGAAGTAAGCATATGG
ATGGGTATGTGTCACAGGAGATTCATGTTTTTGATGATTTTGGTCAAAATCGGGAAGAGGAAGACTAT
TCTTTAATCTGTAACCTCATTTCTAGTGTTCCATTCATCACTCCAAAGGCAAGTGTAGAGGCTAAGGG
CACCCAATACAGAGGACGCCTTGTTGTTGTCACAACCAATAGACGGGATTTCACTTCTTGTAAGTTGA
CTGACCCAGATGCACTGGAAAGGCGCTTCCCTATTCGCCTTAATATCCGGCCATTGCAAAAATATAAC
CACAAGGGCCGTTTGGATGTGGCAACAGCCATGAGAGATGGAAGCTTGCAGGGAGGAACTTGCTG
GGAAAGGGATATAGGCCAATTAGGCCTTGAATGTTGGAATCCCATAAATGGCCAAACCCTGATTGATG

AAATTATGAGTGAGTTGCAGGTTAGGCAAGAAGTAGCTTCCTTTATGAACCAGTCTAAGGTGAGGCGT
TTCTCTGACCCAGAGACACTTTTTAGTGATTTGGAAGATCTAAAATTGGAATTTGATTTTGATCAATTAG
AACAACAAGCTAAACTTTTTGCAAAACCAAAAGAAGGAAAAATCTCTAAATTTAGGGCCTGGGTTAGA
GACTGCACAGGGAAGATTAAGGGTTTTCTGGAGCGAAATCGGGCTTGGATTCTTGGTATAGGAACCC
TTGGAACCATTATGTCACTTGTGACTATGTGTGTCCCATTAGCTAGACGCTTTGCCCAGTCAATCTATT
CACAACAACCTGTGGCCAAAACACTCCCAAAAGATTTTAAGGTAGCTGTTAAGAAACACATTGAGAAA
CTGGAAACTGAATTGGAAGATCAGAGCGGGCGGGTGAATTTCAGACATATTTGTAACAGACTCGTCA
ATGTGAGTAATGAGGATGAGGTAGCCACTGGTTTAGCCATTGGTGGGAAAAATGTCTTGACATTTGG
GCATTCTAAATTTACACAATTGGATGAGATTAGGGACATGCAATTTAATGCACCAGCTAAGGGGACCC
CCATTACTTATGATGGAGAACCCACTGATCTCCAGTTATTGGAATGTGACATACCCCATCAGTTTAAAG
ATGTGTCTAAATTGATTGCCACAGAGGACTATCGTGGTAATGGCTGGTTGGTTTGGAAAGATCAGGAC
CAGTATATGGTGCAAGATGTGACAAAGATTAGGCCTTTTGGTTCTACAACAACTGCATCAGGCACAAC
ATCATGTCAAACATACATATATAATTGCAAGACTGGACCTGGTTCCTGTGGTGGTGTGCTTGTAGCTCA
AATTGGAGGGAATTTGAAGATCCTTGGGATACACACAAGTGGCAATGGCACTATGGGAGCAGCCAAT
AGGGTCTTTCCAGTTTTTAATCAAGGGAAAGTAGTGAGCAAGCAATATGCAGGTAAGATTCTGTACCA
TCAACCCCGAAAAACCGCATATATGAAGTCACCTGTCTATGAGGACTCACCCTATGAGCCCGCAGTT
CTTTCCATCAATGACCAGCGACTTGGGGTGAAGATTGAAGACATGGCTAAGAAAGCATCTGACAAGT
ATATTGGCAATGTGTTTCAGCCTCCTCCTGAGGCTTTCCAACTTGCAAAGACACATGTTGCCGAAAAA
CTGTCTAGAGTCTTGGGATGCCATGACACTATCTCATATGAGTCAGCTGTGAGTAGTGATGTTATACCT
ATGAACTGGGACACTTCGCCTGGCATTAAGTATAAGGGGGAGACCAAACGACAACTGGTCCTGAAGT
CCTCATTCAGACAGGATGTTATGGAGCAACTACAGAGTCCATCCACTGTTTTCACTTGTTATTTGAAA
GATGAACTCCGAAAGAAGGAAAAAATCAAGGAAGGGAAAACAAGAGGCATTGAGGCTTGTAATTTTG
ATCACACTGTGGCTTATAGAATGGTGATGGGTGACATCTTTTCTAACATCTATGATGATTCTTTCATTAT
ATCTGGTTGTGCTGTTGGAATTAATCCTTTTTGTGAGTGGGACAATCTGTTGGCAAATCTCCAACCTT
ACAATTTGTGTCTTGACTTTTCTGGATTCGATGGCTCTCTGAGTGCCCAAATTCTTGAGGAGGCTGTG
GATGTCTTGTCTTATTTCCACAATGACCCAGCCCTAGTGAAAAGGATTCATGCACCAACTATCTATTCA
ACTCATTATGTAACTGATGAGATATGGCAGGTAGAAGGAGGGATGTGTTCAGGATCTCCATGTACCAC
TGTGGTCAATTCTATTGTGAATCAGCTTGCGTGTTACACCATTTTAGCTGTCTTGGGCTATGACATCAA
CCAATGTTATGTTGTTAGTTATGGGGATGACTGTGTTCTGTCAGTGCCAGAGGTGCGCGATATTTCAA
AACTATCGCACTATTTCAAGCTTTTCTTTGGTATGACAGCAACAGCTAGTGACAAAGAAAGTGACATAA
CTTGGCTAGCCCCTATGGAGATAGAATTTCTGAAGAGAACTCCTGCCTTCCTCCCAGGGACCCGCAA
AATCATTGGTGTCCTTGACAAGGAAGTTTTGGAAGGGAAGATACAGTGGTGCAAGGGACCAGAAGC
ATTCAAACAGCAACTTGACTCTTTCTTGTTGGAAGCAGCTCTGCATGGAGAAGAATACTATCATGAGG

> Conserved protein sequence translated by attenuated ORF
MDTLTKNIEDATVNIIGSCAEKVEEAISGLGAVESVASTNSAIATANATTTQTILDPTEGSTDDFYSCSYEVG
AQGDNISRLVHLVTGQWVPNDDYYACLRWLATPACFFQNNTQPAYGQTRYFRFIRCGFHFRLLVNAPSG
SAGALMLVWMPYPYCRVLSGANQIHTNVERRSLMNLPYAILDLRTNTEIDLVVPYVNYRNYVEITTSDTTG
GAICVIVLGKYRHGNGTSNTVDFTLFGELLETDLQCPRPFDNQGKRKPCRRPIHKPKNPPQEPRVIIQPG
PGAANLSNSSVIAMAESTALANEGTAVDYSTAGCASSVDDVIMVLRRWQILASFQWQSTLTPSARINRYQ
LIFRNIPTFSLFFDKFQYWRGSLEVKFMTFGSQFNTGRYQMSWYPIADGEQSLAQCQNSVFVTGDVCAT
PVTLTLPFTSTTWRKSTRDPYGYLMWHVVNRLTVNSSAPSNIDCTVLLRVGKDFQFTAPLYGNLQMATN
NQGDTNQLGDDEPVCFLNFETANVPIQGESHTLVKHLFGRQWLVRTVQHASTVQELDLQVPDRGHASLI

RFFAYFSGEIILTIVNNGTTPAMVAHSYSMDDLSSEYAVTAMGGVMIPANSAKNISVPFYSVTPLRPTRPIP
GTSEATFGRLFMWTQSGSLSVFMGLKKPALFFPLPAPTSTTLSRGSNGVIPTLDQSGDEVDCHFCKICSK
MKRMWKPKGHFRFCLRLKTLAFELDLEIESDQIRNKKDLTTEGVEPNPGPILVVGKSGSGKSRLCNVLAG
VELFESKLSPRSVTMVHQVETIDIEGKKVTIVDSPETPNYDGPISAFFYLIEAGRFTAEDKEYIQMMRKRFP
SFEKSTILILNRADELKTDDDIRNWIKTSGELDSLVRACGGRIAKFHRYKINRAKLLEKAATLPEFVSHLPRL
VYKDRKMYRHYGVQCGSTVFHMDSENILESALNGEVTIKQEKWNGNWKPAGDHMQSTASMYLKSQTM
PKFTFSIDENCETWARHLLGDYGETQGQIFKERLMWAVALGFFMTMKITTDQSFPGKDAIHTVLTKISNFI
FGGLENEVVRVVIRTVIRIVCYLILYIHSPNIVTTGTLVALLALDATSMSMDQGLKTLCMSLVDGDFGKFCSA
LLEKIQTVDEADLKKTIPKFNDMLEDQSGKTTSPKSFNDWTTCAKNVQWWLESFVKVVNWLKEKIFPSK
TDPTLQWLQDHEEHIAIMLALCDEHLCMLRTEKDYICEHNTRPKHQCLVEMVSGTLNQLQGISSARELAA
RLQHVLNKLHQVNFEPELEWTHRPEPLGIWISGGPGVGKSFLSNYIVKEIAKLKHWKSYANPTGSKHMD
GYVSQEIHVFDDFGQNREEEDYSLICNLISSVPFITPKASVEAKGTQYRGRLVVVTTNRRDFTSCKLTDPD
ALERRFPIRLNIRPLQKYNHKGRLDVATAMRDGSLQGGACWERDIGQLGLECWNPINGQTLIDEIMSELQ
VRQEVASFMNQSKVRRFSDPETLFSDLEDLKLEFDFDQLEQQAKLFAKPKEGKISKFRAWVRDCTGKIK
GFLERNRAWILGIGTLGTIMSLVTMCVPLARRFAQSIYSQQPVAKTLPKDFKVAVKKHIEKLETGLEDQSG
RVNFRHICNRLVNVSNEDEVATGLAIGGKNVLTFGHSKFTQLDEIRDMQFNAPAKGTPITYDGEPTDLQLL
ECDIPHQFKDVSKLIATEDYRGNGWLVWKDQDQYMVQDVTKIRPFGSTTTASGTTSCQTYIYNCKTGPG
SCGGVLVAQIGGNLKILGIHTSGNGTMGAANRVFPVFNQGKVVSKQYAGKILYHQPRKTAYMKSPVYEDS
PYEPAVLSINDQRLEVKIEDMAKKASDKYIGNVFQPPPEAFQLAKTHVAEKLSRVLGSHETISYESAVSSD
VIPMNWDTSPGIKYKGETKRQLVLKSSFRQDVMEQLQSPSTVFTCYLKDELRKKEKIKEGKTRGIEACNF
DHTVAYRMVMGDIFSNIYDDSFIMSGCAVGINPFCEWDNLLANLQPYNLCLDFSGFDGSLSAQILEEAVD
VLSYFHNDPALVKRIHAPTIYSTHYVTDEIWQVEGGMCSGSPCTTVVNSIVNQLACYTILAVLGYDINQCY
VVSYGDDCVLSVPEVRDISKLSHYFKLFFGMTATASDKESDITWLAPMEIEFLKRTPAFLPGTRKIIGVLDK
EVLEGKIQWCKGPEAFKQQLDSFLLEAALHGEEYYHEVTSKLKARCPVFDIQPWGVAKLRAYTACMMI.

> Conserved protein sequence translated by virulent ORF

MDTLTKNIEDATVNIIGSCAEKVEEAISGLGAVESVASTNSAIATANATTTQTIPDPTEGSTDDFYSCSYEVG
AQGDNISRLVHLVTGQWVPNDDYYACLRWLATPACFFQNNTQPAYGQTRYFRFIRCGFHFRLLVNAPSG
SAGALMLVWMPYPYCRVLSGTNQIHANVERRSLMNLPYAILDLRTNTEIDLVVPYVNYRNYVEITTSDTTG
GAICVIVLGKYRHGNGTSNTVDFTLFGELLETDLQCPRPFDNQGKRKPRRRPIHKPKNPPQEPRVIIQPG
PGAANLSNSSVIAMAESTALANEGTAVDYSTAGCASSVDDVIMVLRRWQILASFQWQSTLTPSARINRYQ
LIFRNIPTFSLFFDKFQYWRGSLEVKFMTFGSQFNTGRYQMSWYPIADGEQSLAQCQNSVFVTGDVCAT
PVTLTLPFTSTTWRKSTRDPYGYLMWHVVNRLTVNSSAPSNIDCTVLLRVGKDFQFTAPLYGNLQMATN
NQGDSNQLGDDEPVCFLNFETANVPIQGESHTLVKHLFGRQWLVRTVQHASTVQELDLQVPDRGHASLI
RFFAYFSGEIILTIVNNGTTPAMVAHSYSMDDLSSEYAVTAMGGVMIPANSAKNISVPFYSVTPLRPTRPIP
GTSEATFGRLFMWTQSGSLSVFMGLKKPALFFPLPAPTSTTLSRKSNDVIPTLNQSGDEVDCHFCEICSK
MKRRWKPRGYFRFCLRLKTLAFELNLEIESDQIRNKKDLTTEGVEPNPGPILVVGKSGSGKSRLCNVLAG
VELFESKLSPRSVTMVHQVETIDIEGKKVTIVDSPETPNYDGPISAFFYLIEAGRFTAEDKEYIQMMRKRFP
GFEKSTILILNRADELKTDDDIRNWVKTSGELDSLVRACGGRIAKFHWHKINRAKLLEKAATLPEFVSHLP
RLVYKDRKMYRHYGVQCGSTVFHMDSENILESALNGEVTIKQEKWNGNWKPAGDHMQSTASMYLKSQ
TMPKFTFSIDENCETWARHLLGDYGETQGQIFKERLMWAAALGFFMTMKITTDQSFPGKDAIHTVLTKIS
NFIFGGLENEVVRVVIRTVIRIVCYLILYIHSPNIVTTGTLVALLALDATSMSMDQGLKTLCMSLVDGDFGKF
CSALLEKIQTVDEADLKKTIPKFNDMLEDQSGKTTSPKSFNDWTTCAKNVQWWLESFVKVVNWLKEKVF

PSKTDPTLQWLQDHEEHIAIMLALCDEHLCMLRTEKDYICEHNTRPKHQRLVEMVSGTLNQLQGISSARE
LAARLQHVLNKLHQVNFEPELEWTHRPEPLGIWISGGPGVGKSFLSNYIVKEIAKLKHWKSYANPTGSKH
MDGYVSQEIHVFDDFGQNREEEDYSLICNLISSVPFITPKASVEAKGTQYRGRLVVVTTNRRDFTSCKLT
DPDALERRFPIRLNIRPLQKYNHKGRLDVATAMRDGSLQGGTCWERDIGQLGLECWNPINGQTLIDEIMS
ELQVRQEVASFMNQSKVRRFSDPETLFSDLEDLKLEFDFDQLEQQAKLFAKPKEGKISKFRAWVRDCTG
KIKGFLERNRAWILGIGTLGTIMSLVTMCVPLARRFAQSIYSQQPVAKTLPKDFKVAVKKHIEKLETELEDQ
SGRVNFRHICNRLVNVSNEDEVATGLAIGGKNVLTFGHSKFTQLDEIRDMQFNAPAKGTPITYDGEPTDL
QLLECDIPHQFKDVSKLIATEDYRGNGWLVWKDQDQYMVQDVTKIRPFGSTTTASGTTSCQTYIYNCKT
GPGSCGGVLVAQIGGNLKILGIHTSGNGTMGAANRVFPVFNQGKVVSKQYAGKILYHQPRKTAYMKSPV
YEDSPYEPAVLSINDQRLGVKIEDMAKKASDKYIGNVFQPPPEAFQLAKTHVAEKLSRVLGCHDTISYESA
VSSDVIPMNWDTSPGIKYKGETKRQLVLKSSFRQDVMEQLQSPSTVFTCYLKDELRKKEKIKEGKTRGIE
ACNFDHTVAYRMVMGDIFSNIYDDSFIISGCAVGINPFCEWDNLLANLQPYNLCLDFSGFDGSLSAQILEE
AVDVLSYFHNDPALVKRIHAPTIYSTHYVTDEIWQVEGGMCSGSPCTTVVNSIVNQLACYTILAVLGYDIN
QCYVVSYGDDCVLSVPEVRDISKLSHYFKLFFGMTATASDKESDITWLAPMEIEFLKRTPAFLPGTRKIIGV
LDKEVLEGKIQWCKGPEAFKQQLDSFLLEAALHGEEYYHEVTSKLKARCPVLDIQPWGVAKLRAYTACM
MI.

Supplement Figures

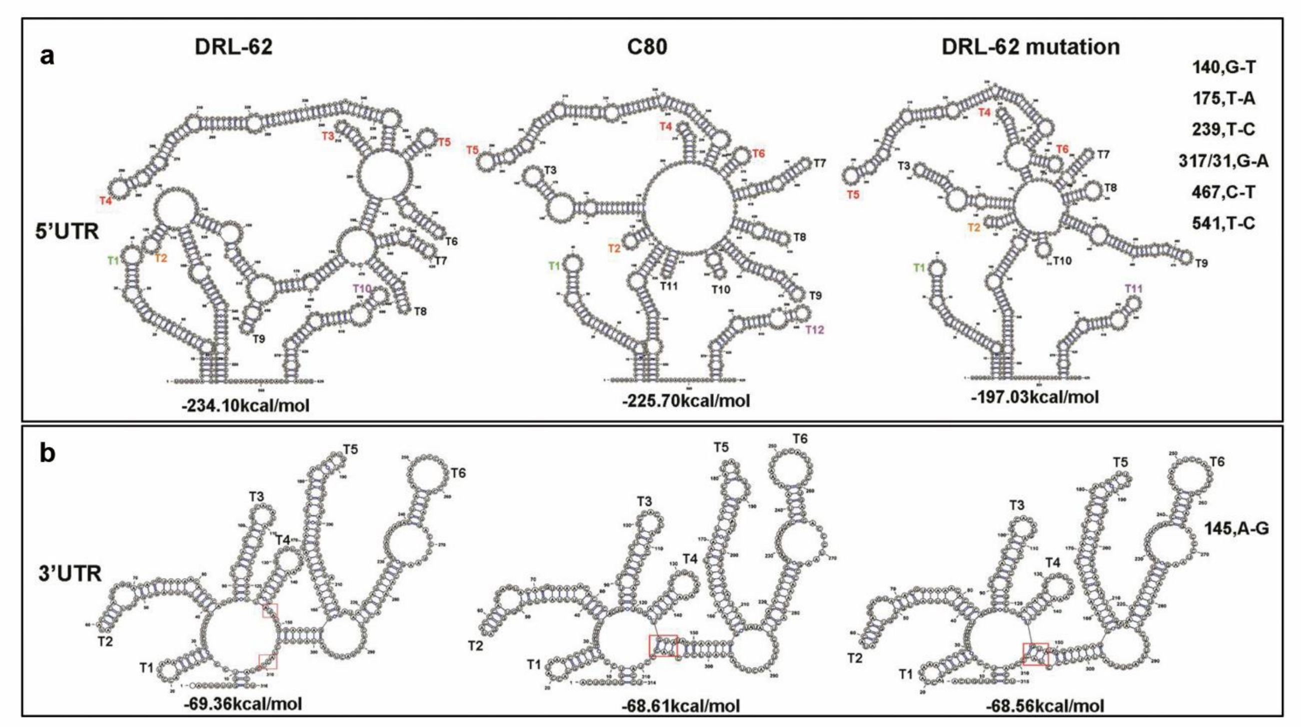

Supplementary Figure 1 Secondary structural variations of 5'UTR and 3'UTR in the process of serial passaging in chick embryos. (a) Online RNAfold web server was used to characteristic the secondary structural variation in chick embryo attenuated strains. In order to understand whether those fixed SNPs could significantly change the secondary structure of virulent strains, the 5’UTR of artificial mutant involved in the Table S2 were also imported into RNAfold web server to predict their secondary structure. (b) The same methods were also used to predict secondary structure of 3’UTR and their mutant. Apparently structural changes in 5’UTR but not in 3’UTR were identified in the DHAV-1 genome.

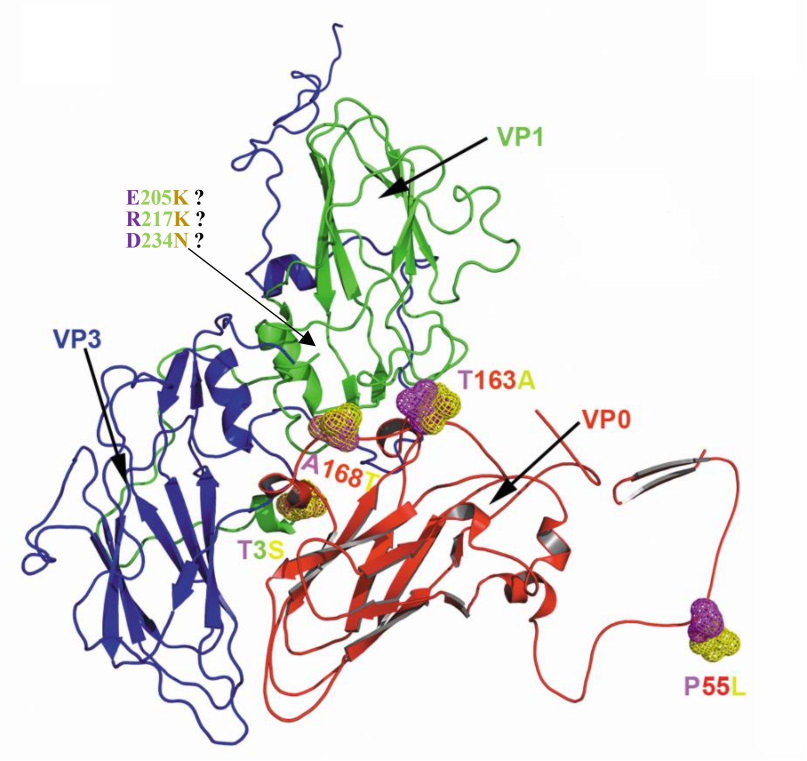

Supplementary Figure 2 Quarternary structural variations in capsid. Non-synonymous substitutes, such as T3S, were displayed using mesh model (purple to yellow) to indicated their spatial position. The color of number indicates where the non-synonymous substitutes take place, VP1(green), VP3(blue) or VP0(red). Due to the sequence difference with the modeling template, except T3S mutation of VP1, the other three C-terminal mutations (E205K, R217K and D234N) in spatial position were not available in this analysis. Three substitutes (P55L, T163A, A168T) of VP0 were also labeled. Those aa substitutes were mainly identified in the interface of VP1/0/3. All those modeling are predicted by SWISS-MODEL and visualized by Pymol software.

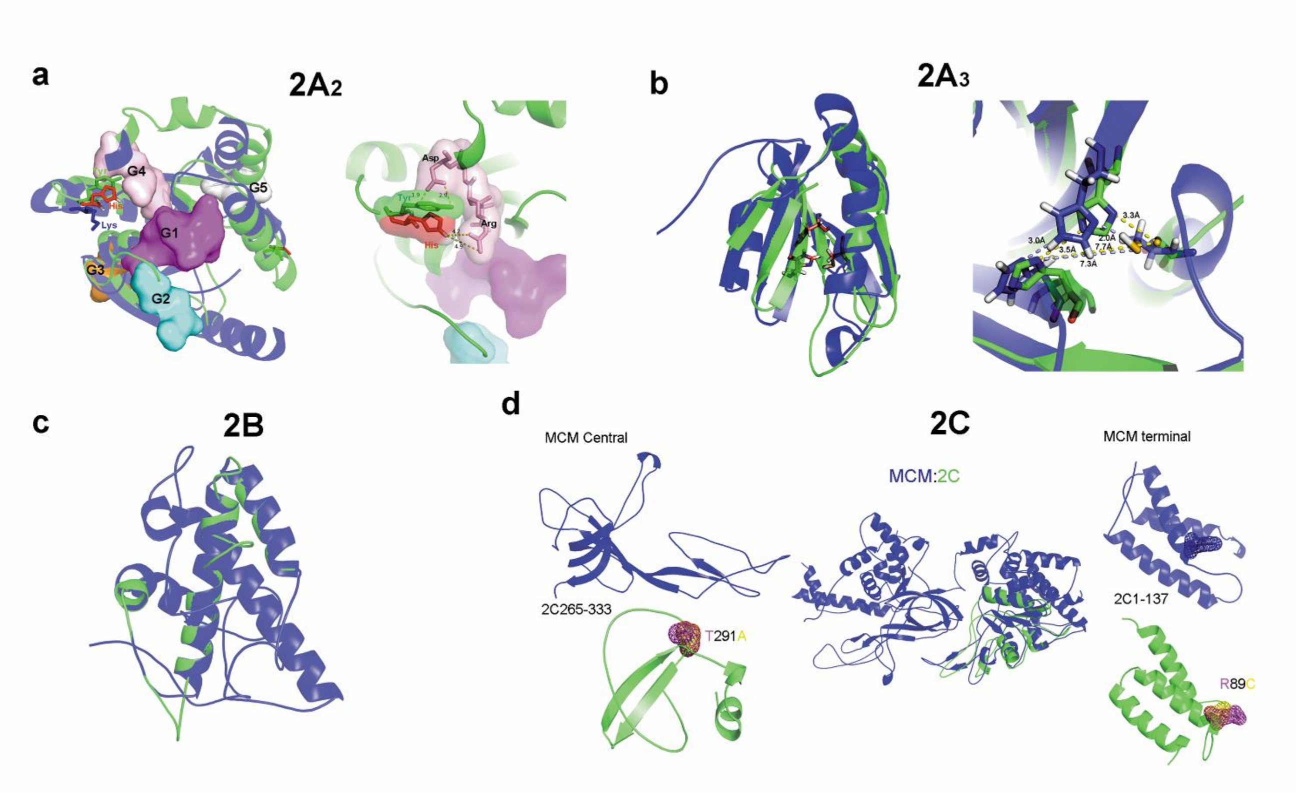

Supplementary Figure 3 Tertiary structural variations in P2-nonstructural proteins. Those nonstructural proteins ($2A_2^{pro}$,$2A_3^{pro}$, $2B^{pro}$ and $2C^{pro}$) coded in this region were well modeled with its template, except for 2C protein. Blue color indicates templates. **a**. Superposition of 2A2 protein with its template (GTPase, PDB: 3zjc.3.A). G1-G5 motif were labeled by different color. The mutation H142Y nearby G4 box changed from a basic aa to an aromatic aa, may attribute to the effect of maladaptive chick embryos. **b**. Superposition of 2A3 protein with Adipose phospholipase A (PDB: 4fa0.1.A). **c**. Superposition of 2B protein with Actin-related protein (PDB: 4jd2.1.E). **d**. Superposition of 2C protein with Minichromosome maintenance protein (MCM)(PDB: 4r7y.1.A). Because 2C protein were not predicted in full length, the DHAV-1 2C protein was truncated as three fragments to build its tertiary structure. Non-synonymous

substitutes were also displayed using mesh model (purple to yellow) to indicated their spatial position. All those modeling are predicted by SWISS-MODEL and visualized by Pymol software.

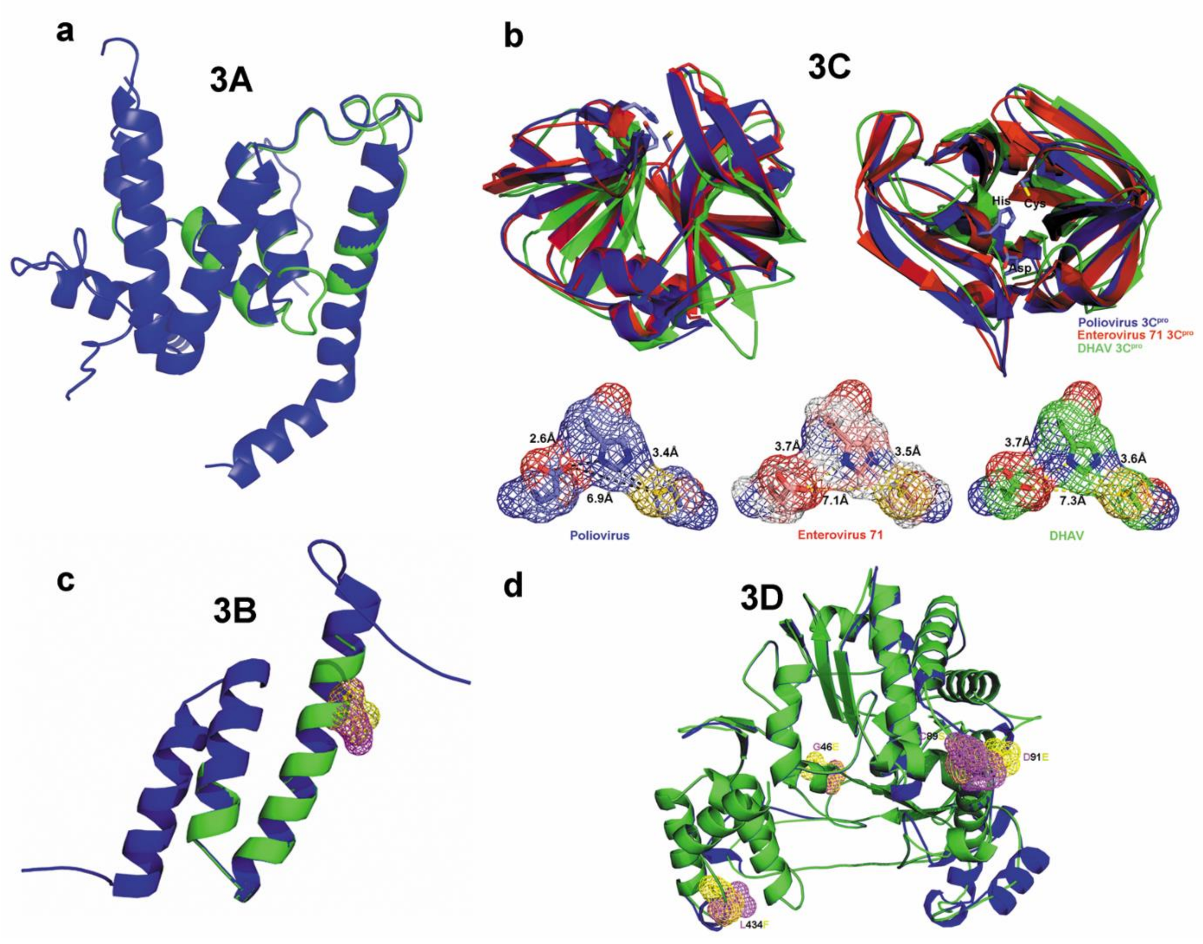

Supplementary Figure 4 Tertiary structural variations in P3 non-structure proteins. Blue color indicates templates. **a**. Superposition of 3A protein with its template (AopB, PDB: 3wxx.2.B). There were no substitutes in 3A protein. **b**. Superposition of 3C protein with Enterovirus 71 3C protein (PDB: 3qzq.4.A). The catalytic resides and their distance were also labeled. There were no substitutes in 3C

protein. **c**. Superposition of 3B protein with iron-regulated surface determinant protein H (PDB: 2lhr.1.A). Non-synonymous substitutes were also displayed using mesh model (purple to yellow) to indicated their spatial position. The mutation of E30G at α-helix was labeled (purple to yellow). **d**. Superposition of 3D protein with Poliovirus 3D polymerase (PDB: 4nlr.1.A). Four substitutes (G46E, C89S, D91E and L434F) were identified on the surface loop of 3D^{pro}. All those modeling are predicted by SWISS-MODEL and visualized by Pymol software.

Supplement Tables

Supplementary Table 1 Representative viral strains in this analyses

Groups	Strains	Accession no.	Comments
Chicken embryo attenuated strains	CH60	KU923754.1	60 passages (china, Sichuan)
	C80	DQ864514	80 passages (china, ?)
	FC64	HQ232302	64 passages (china Fujian)
	A66	DQ886445	66 passages (china, Anhui)
	MY	GU944671.1	86 passages (china, Xichang)
Virulent strains	DRL-62	DQ219396.1	ATCC(Korea)
	R85952	DQ226541	ATCC(Korea)
	03D	DQ249299	China Taiwan
	JX	EU371557	China
	F	EU264072	China Beijing
	5886	DQ249301	USA
	H	DQ249300	UK
	HP-1	EF151312	China Ha'erbin
	ZJ	EU841005	China Zhanjiang
	S	EF417871.1	China Beijing
	CL	EF427899.1	China Shandong
	YZ	EF427900.1	China Yangzhou
	DHV-HS	DQ812094.2	South Korea
	DHV-HSS	DQ812092.1	South Korea
	JH2	EU395435.1	China Shandong
	JH1	EU395436.1	China
	YN	EU395437	China
	FS	EU395438	China Foshan
	ZZ	EU395439.1	China Shandong
	DHAV-1	EU395440.1	China
	GZ	EU888310	China Guangzhou
	HDHV1-BJ	FJ157172.1	China
	HDHV1-JX	FJ157173.1	China
	HDHV1-SC	FJ157174.1	China
	HDHV1-GS	FJ157175.1	China
	HDHV1-HN	FJ157177.1	China
	HDHV1-SH	FJ157178.1	China
	HDHV1-GD	FJ157179.1	China
	HDHV1-HB	FJ157180.1	China
	LY0801	FJ436047	China Shandong
	FFZ05	FJ496340	China Fujian

GFS06	FJ496341.1	China Fujian
GQY07	FJ496342.1	China Guangdong
X	FJ496343.1	China Sichuan
GFS99	FJ496344.1	China Guangdong
SG	FJ971623	China Shandong
NA	GQ130377	China Fuzhou
C-LGJ	GU066819	China
C-XNH	GU066820	China
C-QYD	GU066825	China
SH-1	HQ232303.1	China
SH	HQ265433.1	China Shanghai
Du/CH/LBJ	JF828982	China
Du/CH/LFJ	JF828983	China
duck/CH/LGD/100913	JF828984	China
duck/CH/LGD/100915	JF828985	China
duck/CH/LGD/100916	JF828986	China
duck/CH/LGD/100922	JF828987	China
duck/CH/LGD/100928	JF828988	China
duck/CH/LSD/090830	JF828989	China
duck/CH/LSD/101003	JF828990	China
duck/CH/LSD/101004	JF828991	China
duck/CH/LSD/101006	JF828992	China
duck/CH/LYN/080704	JF828993	China
duck/CH/LBJ/090809	JF828997	China
HB02	JQ031262	China Hebei
Duck/LGD/111238	JQ804521	China
Duck/LGD/111239	JQ804522	China
FZ86	JX390982	China Fuzhou
FZ05	JX390983	China Fuzhou
FZ99	JX390984	China Fuzhou
FJ1220	KC904272	China Fujian (Pigeon)
161/79/V	EU753359	China Jiangsu
R	EF585200	China Guangzhou
SY5	JQ808453	China Zhejiang
Du/CH/JS2013	KP721458	China Jiangshu
GD	KM017068	China Guangdong
CH2012	KF953535	China

frontiers

Supplementary Table 2 Fixed SNPs in the genome of attenuated and virulent strains.

Gene	5'UTR	VP0	VP3	VP1	2A1	2A2	2A3
Synonymous mutation	137,G-T<br>172,T-A<br>236,T-C<br>314/28,G-A<br>464,C-T<br>538,T-C	582,GTG-GTA<br>645,GTG-GTC	54,CAG-CAA<br>105,AAC-AAT<br>318,TTC-TTT<br>372,TTC-TTT<br>471,GTG-GTA<br>705,AAT-AAC	16/8,TTG-CTA<br>174,GTT-GTC<br>180,GAC-GAT<br>267,GCA-GCG<br>273,GTA-GTG<br>342,ATT-ATC<br>348,GCT-GCC<br>588,GGG-GGA	51,CCA-CCG<br>57,CCG-CCA	48,TGT-TGC<br>138,GAG-GAA<br>168,CCT-CCC<br>246,AAG-AAA<br>462,GCC-GCT	72,GTC-GTT 78,CAC-CAT<br>135,CAG-CAA 189,GCC-GCT<br>312,GAG-GAA
Missense mutation	None	164,CCA-CTA<br>(55,Pro-Leu)<br>487,ACT-GC/AT<br>(163,Thr-Ala/Asp)<br>502,GCA-ACA<br>(168,Ala-Thr)	None	7,TCC-ACC<br>(3,Ser-Thr) 613,GAA-AAA<br>(205,Glu-Lys)<br>650,AGA-AAA<br>(217,Arg-Lys)<br>700,AAT-GAT<br>(234,Asn-Asp)	None	280,GGC-AGC<br>(94,Gly-Ser)<br>424/6,CAC-TAT<br>(142,His-Tyr)	329,GCA-GTA(110,Ala-Val)
Nonsyn/Syn		3/2=1.5	0/6=0	4/9=0.44	0/2=0	3/5=0.6	1/5=0.2

Gene	3'UTR	2B	2C	3A	3B	3C	3D
Synonymous mutation	144A-G	126,CTT-CTC<br>183,TTA-CTA<br>243,CTG-CTA<br>324,ACT-ACC	159,CAT-CAC<br>246,AAC-AAT<br>528,GGA-GGG<br>672,GCT-GCC<br>684,CAA-CAG<br>690,AGA-AGG<br>738,TGT-TGC<br>792,ATC-ATT<br>843,GCC-GCT	33,ACA-ACT<br>82,84,TTA-CTC<br>189,CGA-CGG<br>231,ATT-ATC	45,GAT-GAC<br>79,CTA-TTA	33,AAC-AAT<br>270,ACA-ACG<br>291,GGC-GGT<br>486,CAC-CAT	96,CCC-CCT 144,AAG-AAA<br>252,TCT-TCC 327,GAC-GAT<br>402,GTT-GTC 552,GAC-GAT<br>849,TCT-TCC 858,ACC-ACT<br>861,ACT-ACC 912,GTC-GTA<br>1128,ACC-ACT
Missense mutation	None	None	112/4,GTG-ATA<br>(38,Val-Ile)<br>265,CGT-TGT<br>(89,Arg-Cys)<br>871,ACT-GCT<br>(291,Thr-Ala)	None	89,GAA-GGA<br>(30,Glu-Gly)	None	137,GGG-GAG(46,Gly-Glu)<br>265,TGC-AGC(89,Cys-Ser)<br>273,GAT/C-GAA(91,Asp-Glu)<br>1300,CTT-TTT(434,Leu-Phe)
Nonsyn/Syn		0/4=0	3/9=0.33	0/4=0	1/2=0.5	0/4=0	4/11=0.36

Supplementary Table 3 RSCU of Virulent strains and attenuated strains used in this study

Strains	Leu UUA	Leu CUA	Val GUA	Ser UCA	Thr ACA	Pro CCA	Ala GCA	Lys AAA	Gln CAA	Gly GGA	Arg AGA	Arg CGA	Glu GAA	IleA UA	Ser AGU	Asp GAU	Arg CGU
A66	0.67	0.64	0.57	1.85	1.67	2	1.42	1.16	1.09	1.07	1.87	0.68	1.17	0.62	0.94	1.27	0.45
C80	0.64	0.67	0.57	1.88	1.66	1.98	1.42	1.16	1.1	1.07	1.87	0.68	1.16	0.62	0.96	1.31	0.45
CH60	0.67	0.64	0.57	1.86	1.66	1.98	1.42	1.16	1.1	1.08	1.87	0.68	1.16	0.62	1.03	1.31	0.45
FC64	0.67	0.64	0.58	1.91	1.65	1.98	1.42	1.17	1.1	1.12	1.85	0.67	1.15	0.64	0.99	1.32	0.45
MY	0.67	0.64	0.57	1.85	1.66	1.98	1.42	1.17	1.1	1.08	1.92	0.68	1.16	0.62	1.06	1.3	0.45
3D	0.67	0.55	0.56	1.82	1.71	2.02	1.42	1.12	1.1	1.04	1.98	0.72	1.22	0.62	1.08	1.33	0.55
LSD/090830	0.75	0.51	0.5	1.78	1.64	1.96	1.4	1.11	1.04	1.09	1.96	0.71	1.2	0.64	1.12	1.27	0.44
C-XNH	0.58	0.77	0.46	1.78	1.64	1.91	1.45	1.1	1.07	1.03	1.98	0.61	1.19	0.64	1.18	1.34	0.5
FZ99	0.61	0.67	0.54	1.82	1.58	1.93	1.47	1.07	1.09	0.97	1.93	0.66	1.24	0.6	1.12	1.34	0.5
ZJ	0.58	0.64	0.57	1.92	1.65	1.96	1.38	1.1	1.04	0.95	1.96	0.65	1.25	0.6	1.08	1.33	0.49
NA	0.65	0.65	0.55	1.92	1.66	1.96	1.44	1.1	1.04	0.95	1.93	0.66	1.23	0.59	1.04	1.37	0.5

Strains	Gly GGU	Cys UGU	Asn AAU	Tyr UAU	His CAU	Ala GCU	Phe UUU	Leu CUU	Val GUU	Pro CCU	Thr ACU	IleA UU	Ser UCU	Gln CAG	Ala GCG	Arg CGG	Gly GGG
A66	1.01	1.33	1.37	1.47	1.18	1.51	1.35	1.38	1.35	1.26	1.5	1.69	1.89	0.91	0.09	0.68	0.91
C80	1.02	1.33	1.37	1.44	1.22	1.51	1.38	1.41	1.35	1.27	1.52	1.69	1.85	0.9	0.09	0.68	0.91
CH60	1	1.33	1.35	1.5	1.22	1.51	1.38	1.41	1.35	1.27	1.52	1.69	1.82	0.9	0.09	0.68	0.92
FC64	1.01	1.36	1.36	1.45	1.25	1.51	1.4	1.41	1.32	1.27	1.5	1.68	1.8	0.9	0.09	0.67	0.88
MY	1	1.33	1.35	1.53	1.22	1.48	1.4	1.41	1.35	1.27	1.52	1.71	1.81	0.9	0.12	0.68	0.92
3D	1.01	1.55	1.38	1.44	1.16	1.39	1.43	1.35	1.39	1.17	1.61	1.73	1.82	0.9	0.09	0.61	0.93
LSD/090830	1.09	1.48	1.35	1.46	1.35	1.49	1.41	1.44	1.41	1.26	1.56	1.88	1.74	0.96	0.18	0.6	0.85
C-XNH	1.05	1.43	1.4	1.53	1.18	1.39	1.35	1.35	1.46	1.17	1.52	1.75	1.71	0.93	0.09	0.66	0.95
FZ99	1.03	1.43	1.35	1.5	1.22	1.47	1.35	1.32	1.41	1.15	1.6	1.83	1.78	0.91	0.09	0.66	0.97
ZJ	0.95	1.55	1.39	1.5	1.18	1.47	1.4	1.49	1.39	1.26	1.6	1.83	1.85	0.96	0.15	0.65	1
NA	0.97	1.55	1.33	1.55	1.16	1.47	1.38	1.48	1.42	1.26	1.59	1.83	1.85	0.96	0.12	0.61	0.97

Strains	Glu GAG	Lys AAG	Thr ACG	Leu UUG	Ser UCG	Pro CCG	Val GUG	Leu CUG	Ala GCC	Asp GAC	Cys UGC	Arg CGC	Ser AGC	Arg AGG	Gly GGC	Tyr UAC	His CAC
A66	0.83	0.84	0.08	1.65	0.08	0.07	1.27	0.7	0.98	0.73	0.67	0.96	0.53	1.36	1.01	0.53	0.82
C80	0.84	0.84	0.07	1.62	0.08	0.07	1.27	0.73	0.98	0.69	0.67	0.96	0.54	1.36	0.99	0.56	0.78
CH60	0.84	0.84	0.07	1.65	0.08	0.07	1.27	0.7	0.98	0.69	0.67	0.96	0.53	1.36	1	0.5	0.78
FC64	0.85	0.83	0.08	1.65	0.08	0.07	1.29	0.7	0.98	0.68	0.64	1.01	0.54	1.35	0.99	0.55	0.75
MY	0.84	0.83	0.07	1.65	0.08	0.07	1.25	0.7	0.98	0.7	0.67	0.96	0.53	1.3	1	0.47	0.78
3D	0.78	0.88	0.08	1.68	0.12	0.07	1.27	0.77	1.1	0.67	0.45	0.94	0.39	1.21	1.01	0.56	0.84
LSD/090830	0.8	0.89	0.13	1.71	0.19	0.07	1.31	0.69	0.93	0.73	0.52	1.04	0.35	1.25	0.96	0.54	0.65
C-XNH	0.81	0.9	0.1	1.82	0.15	0.15	1.36	0.58	1.06	0.66	0.57	0.99	0.3	1.27	0.97	0.47	0.82
FZ99	0.76	0.93	0.1	1.78	0.15	0.15	1.33	0.67	0.96	0.66	0.57	0.99	0.35	1.27	1.03	0.5	0.78
ZJ	0.75	0.9	0.07	1.68	0.15	0.07	1.32	0.76	0.99	0.67	0.45	0.98	0.35	1.25	1.11	0.5	0.82
NA	0.77	0.9	0.07	1.69	0.12	0.07	1.34	0.71	0.97	0.63	0.45	0.99	0.38	1.32	1.11	0.45	0.84

Strains	Asn AAC	Phe UUC	Leu CUC	Ile AUC	Val GUC	Ser UCC	Pro CCC	Thr ACC
A66	0.63	0.65	0.95	0.69	0.81	0.72	0.67	0.75
C80	0.63	0.62	0.92	0.69	0.81	0.69	0.67	0.75
CH60	0.65	0.62	0.92	0.69	0.81	0.68	0.67	0.75
FC64	0.64	0.6	0.92	0.68	0.82	0.69	0.67	0.77
MY	0.65	0.6	0.92	0.67	0.83	0.68	0.67	0.75
3D	0.62	0.57	0.98	0.65	0.78	0.77	0.73	0.6
LSD/090830	0.65	0.59	0.9	0.48	0.78	0.81	0.7	0.68
C-XNH	0.6	0.65	0.89	0.61	0.72	0.87	0.77	0.75
FZ99	0.65	0.65	0.95	0.57	0.72	0.77	0.78	0.72
ZJ	0.61	0.6	0.85	0.57	0.72	0.65	0.7	0.67
NA	0.67	0.62	0.83	0.59	0.68	0.69	0.7	0.67

Supplementary Table 4 Codon usage bias index of virulent strains and chick embryo attenuated strains

Strains	T3s	C3s	A3s	G3s	CBI	Fop	Nc	GC3s	GC	Gravy	Aromo
MY	0.4333	0.233	0.363	0.2344	-0.097	0.365	51.61	0.36	0.428	-0.24344	0.095598
A66	0.4283	0.2375	0.3621	0.2353	-0.089	0.37	51.72	0.365	0.43	-0.24571	0.094709
FC64	0.4317	0.2339	0.3634	0.235	-0.099	0.364	51.67	0.361	0.428	-0.24335	0.096487
C80	0.4316	0.2347	0.362	0.236	-0.093	0.367	51.72	0.362	0.428	-0.24211	0.095598
CH60	0.4327	0.2336	0.3618	0.2359	-0.097	0.365	51.64	0.361	0.428	-0.24109	0.095598
03D	0.4362	0.2286	0.3617	0.2359	-0.104	0.36	51.1	0.358	0.428	-0.24486	0.094709
ZJ	0.444	0.2209	0.3555	0.2429	-0.102	0.361	50.71	0.356	0.427	-0.25153	0.094709
03D	0.4362	0.2286	0.3617	0.2359	-0.104	0.36	51.1	0.358	0.428	-0.24486	0.094709
ZJ	0.444	0.2209	0.3555	0.2429	-0.102	0.361	50.71	0.356	0.427	-0.25153	0.094709
NA	0.4439	0.2217	0.3573	0.2413	-0.101	0.362	50.62	0.355	0.427	-0.25576	0.094264
FZ99	0.4378	0.2278	0.3543	0.2443	-0.1	0.363	51.4	0.363	0.428	-0.24967	0.095153
C-XNH	0.4364	0.2282	0.3551	0.2441	-0.106	0.359	51.36	0.363	0.428	-0.25216	0.095598
Du/CH/LSD/090830	0.4417	0.222	0.3559	0.2426	-0.103	0.36	51.16	0.358	0.427	-0.24122	0.094709
HB02	0.4362	0.2281	0.3642	0.2341	-0.1	0.362	51.11	0.356	0.427	-0.25651	0.094709
FJ1220	0.4442	0.2227	0.3559	0.2395	-0.101	0.362	50.63	0.356	0.428	-0.24264	0.095153
GD	0.4438	0.2222	0.3555	0.2405	-0.104	0.36	50.6	0.356	0.429	-0.24193	0.094709
Du/CH/LGD/111238	0.447	0.2199	0.3569	0.2386	-0.101	0.362	50.33	0.353	0.427	-0.24896	0.095598
Du/CH/LGD/111239	0.4467	0.2198	0.3575	0.2379	-0.099	0.363	50.43	0.352	0.427	-0.24166	0.095598

frontiers

Supplementary Table 5 Comparative analysis of codon usage frequencies and tRNA copies in chickens and ducks.

tRNA	GluTTC	LysTTT	ArgTCT	GlyTCC	AlaAGC	IleAAT	ValAAC	ThrAGT	ArgACG	CysACA	SerACT
Attenuated-Codon	GluGAA	LysAAA	ArgAGA	GlyGGA	AlaGCU	IleAUU	ValGUU	ThrACU	ArgCGU	CysUGU	SerAGU
A66	74	78	33	40	51	74	52	60	8	36	25
C80	73	77	33	40	51	74	52	61	8	36	25
CH60	73	77	33	40	51	74	52	61	8	36	27
MY	73	78	34	40	50	74	52	61	8	36	28
FC64	72	77	33	42	51	74	50	60	8	36	26
Mean	73.00	77.40	33.20	40.40	50.80	74.00	51.60	60.60	8.00	36.00	26.20
Chicken-tRNA	9	6	4	7	24	7	7	4	8	None	None
Chicken-Codon	31.00	27.30	12.20	17.60	20.80	16.80	13.10	13.30	5.40	8.80	11.20
Virulent-Codon	GluGAA	LysAAA	ArgAGA	GlyGGA	AlaGCU	IleAUU	ValGUU	ThrACU	ArgCGU	CysUGU	SerAGU
ZJ	78	72	36	35	49	80	54	65	9	41	28
3D	76	74	36	39	47	75	55	64	10	41	28
duck/CH/LSD/090830	75	73	36	41	50	79	56	62	8	40	29
C-XNH	74	73	36	38	46	77	57	61	9	38	31
FZ99	77	71	35	36	49	80	55	65	9	38	29
NA	77	72	35	35	50	81	54	64	9	41	27
Mean	76.17	72.50	35.67	37.33	48.50	78.67	55.17	63.50	9.00	39.83	28.67
Duck-tRNA	7	3	2	7	11	6	8	6	9	None	None
Duck-Codon	27.40	25.80	10.90	16.40	21.10	15.20	10.70	13.20	5.40	8.10	10.40

tRNA<br>Attenuated-Codon	ValCAC<br>ValGUG	GlnCTG<br>GlnCAG	LeuCAA<br>LeuUUG	SerCGA<br>SerUCG	ArgCCU<br>ArgAGG	GluCTC<br>GluGAG	LysCTT<br>LysAAG
A66	49	44	54	2	24	53	56
C80	49	44	53	2	24	53	56
CH60	49	44	54	2	24	53	56
MY	48	44	54	2	23	53	55
FC64	49	44	54	2	24	53	55
Mean	48.80	44.00	53.80	2.00	23.80	53.00	55.60
Chicken-tRNA	7	5	3	2	None	7	6
Chicken-Codon	28.20	32.60	12.60	5.20	11.70	40.90	34.30
Virulent-Codon	ValGUG	GlnCAG	LeuUUG	SerUCG	ArgAGG	GluGAG	LysAAG
ZJ	51	47	55	4	23	47	59
3D	50	44	55	3	22	49	58
duck/CH/LSD/090830	52	47	57	5	23	50	58
C-XNH	53	46	59	4	23	50	60
FZ99	52	45	58	4	23	47	62
NA	51	48	55	3	24	48	59
Mean	51.50	46.17	56.50	3.83	23.00	48.50	59.33
Duck-tRNA	6	2	3	2	None	8	7
Duck-Codon	29.00	32.60	11.10	3.90	12.00	38.70	34.50

tRNA	ValGAC	SerGCT	IleGAT	ThrGGT	ProGGG	ArgGCG	CysGCA
Attenuated-Codon	ValGUC	SerAGC	IleAUC	ThrACC	ProCCC	ArgCGC	CysUGC
A66	31	14	30	30	18	17	18
C80	31	14	30	30	18	17	18
CH60	31	14	30	30	18	17	18
MY	32	14	29	30	18	17	18
FC64	31	14	30	31	18	18	17
Mean	31.20	14.00	29.80	30.20	18.00	17.20	17.80
Chicken-tRNA	1	7	None	None	None	None	11
Chicken-Codon	13.60	20.20	22.00	16.50	17.00	10.40	13.30
Virulent-Codon	ValGUC	SerAGC	IleAUC	ThrACC	ProCCC	ArgCGC	CysUGC
ZJ	28	9	25	27	19	18	12
3D	31	10	28	24	20	17	12
duck/CH/LSD/090830	31	9	20	27	19	19	14
C-XNH	28	8	27	30	21	18	15
FZ99	28	9	25	29	21	18	15
NA	26	10	26	27	19	18	12
Mean	28.67	9.17	25.17	27.33	19.83	18.00	13.33
Duck-tRNA	1	7	None	None	None	None	13
Duck-Codon	16.70	24.20	24.40	21.40	19.70	10.40	14.80
